# Incorporation of a Phosphino(pyridine) Subcomponent Enables the Formation of Cages with Homobimetallic and Heterobimetallic Vertices

**DOI:** 10.1021/jacs.2c02261

**Published:** 2022-05-05

**Authors:** John P. Carpenter, Tanya K. Ronson, Felix J. Rizzuto, Théophile Héliot, Peter Grice, Jonathan R. Nitschke

**Affiliations:** Department of Chemistry, University of Cambridge, Lensfield Road, Cambridge, CB2 1EW, United Kingdom

## Abstract

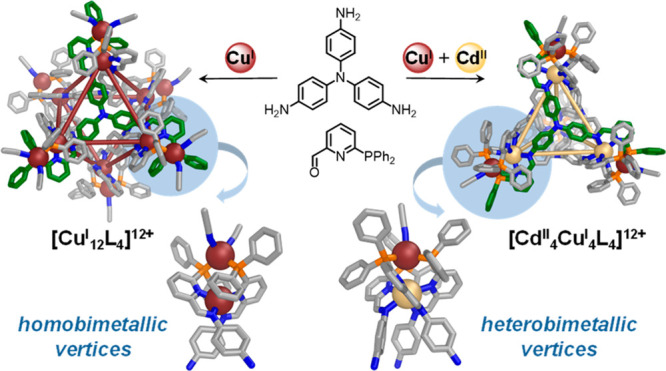

Biological systems employ multimetallic assemblies to achieve a range of functions. Here we demonstrate the preparation of metal–organic cages that contain either homobimetallic or heterobimetallic vertices. These vertices are constructed using 2-formyl-6-diphenylphosphinopyridine, which forms ligands that readily bridge between a pair of metal centers, thus enforcing the formation of bimetallic coordination motifs. Two pseudo-octahedral homometallic M^I^_12_L_4_ cages (M^I^ = Cu^I^ or Ag^I^) were prepared, with a head-to-head configuration of their vertices confirmed by X-ray crystallography and multinuclear NMR for Ag^I^. The phosphino-pyridine subcomponent also enabled the formation of a class of octanuclear Cd^II^_4_Cu^I^_4_L_4_ tetrahedral cages, representing an initial example of self-assembled cages containing well-defined heterobimetallic vertices.

Coordination-driven self-assembly provides a powerful tool for the preparation of intricate and functional architectures with relative synthetic ease.^1^ The combination of metal ions with well-defined stereoelectronic preferences and ligands that have a rigid arrangement of binding sites has enabled the rational design of polyhedral cage architectures including tetrahedra,^2^ cubes,^3^ octahedra,^4^ and higher-order structures.^5^ These cages have attracted considerable interest due to their ability to bind guests within well-defined inner cavities, within which the chemical reactivity and dynamics of guest molecules may be altered.^6^

Most metal–organic cages contain monometallic vertices, as the design principles for these vertices are relatively well-understood. Increased structural complexity and diversity are enabled by the presence of vertices formed from bimetallic units^7^ or more complex clusters.^8^ Such vertices can also increase the functional complexity, because multiple metal ions can bring about new reactivity.^9^ Heterometallic structures^10^ are challenging to synthesize in a controlled manner, requiring strategies that include the incorporation of preformed kinetically inert metal–organic building blocks,^11^ the use of a mixture of hard and soft ligands that bind different metals preferentially,^12^ or the use of ligands with different denticities.^13^

Recently we explored the use of 2-formyl-1,8-napthyridine to prepare cages incorporating disilver vertices.^14^ Herein we employ 2-formyl-6-diphenylphosphinopyridine **A**, a subcomponent containing both N and P donors with nonconverging coordination vectors, as a general method for the construction of metal–organic cages having either homobimetallic or heterobimetallic vertices. Subcomponent **A** was previously incorporated into a dicopper(I) motif,^15^ which was integrated into extended architectures when flexible dianilines were used in combination with rigid carboxylate templates. We reasoned that the combination of **A** with a more rigid, tritopic aniline would enable the synthesis of more complex metal–organic cages, where the dicopper(I) motif would bring together two aniline residues at the vertices of the cage, without requiring carboxylate templation.

The reaction of **A** (12 equiv), tris(4-aminophenyl)amine **B** (4 equiv), and [Cu^I^(MeCN)_4_](OTf) (12 equiv, ^–^OTf = trifluoromethanesulfonate, triflate)^16^ led to the formation of Cu^I^_12_L_4_ cage **1** (Figure 1a), the composition of which was confirmed by ESI-MS. The ^1^H NMR spectrum of **1** indicated the formation of a high-symmetry product in solution, with the ligand in an environment having 3-fold symmetry. ^1^H DOSY NMR further confirmed that the aromatic signals corresponded to a single species (Figure 1b).

**Figure 1:**
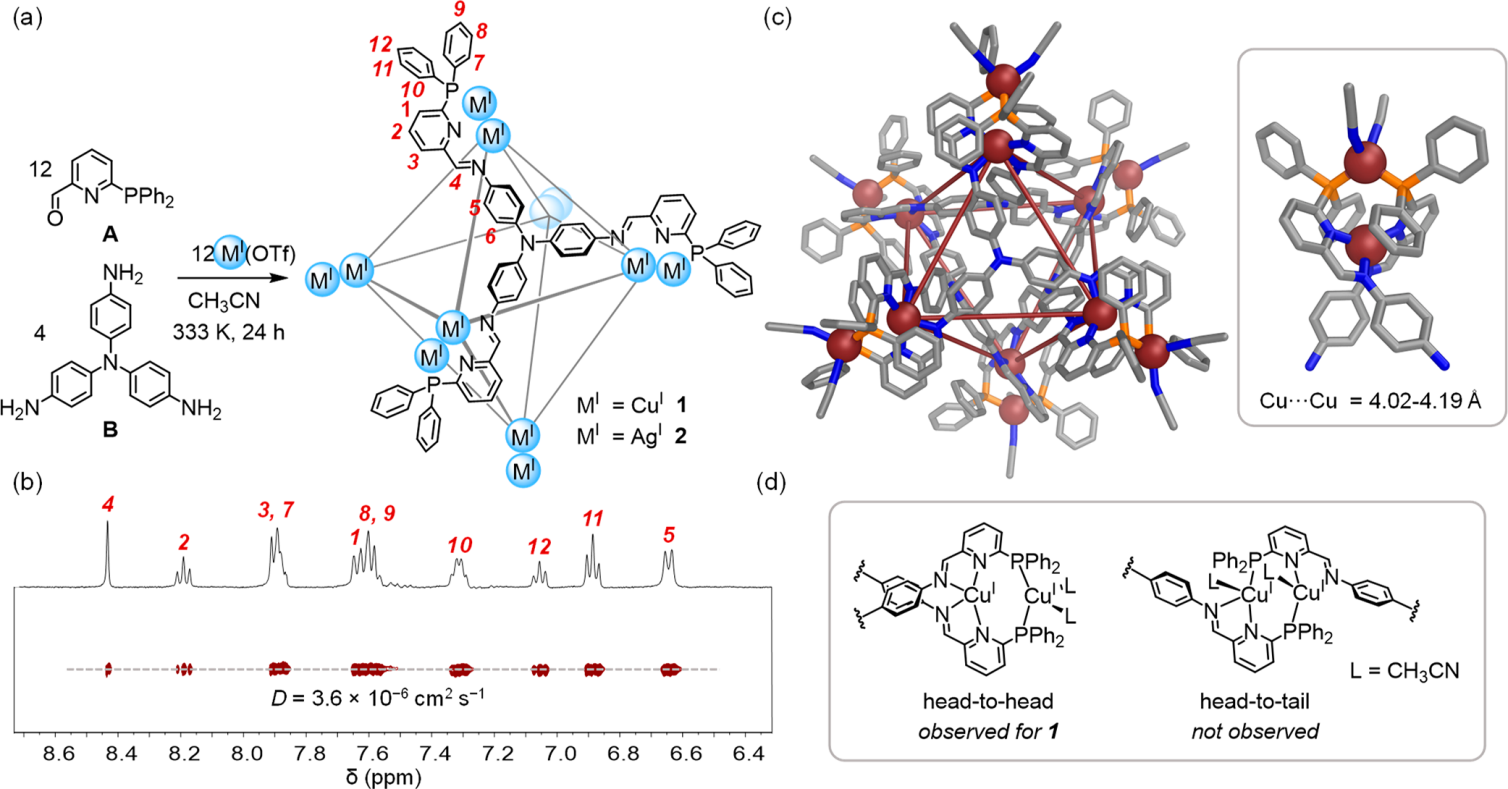
(a) Subcomponent self-assembly of M^I^_12_L_4_ cages **1** and **2**. Externally coordinated acetonitrile molecules are omitted for clarity. (b) ^1^H and DOSY NMR spectra of **1**. The signal for *H*_6_ is not observed at 298 K (see Supporting Information Section 1.2).^19^ (c) Crystal structure of **1** with inset showing one of its dicopper(I) vertices. Disorder, anions, solvent of crystallization, and hydrogen atoms are omitted for clarity. (d) Illustration of the head-to-head vertex geometry observed for **1** and the alternative head-to-tail arrangement previously observed in analogous dinuclear Cu^I^ complexes.^15^

The crystal structure of **1** revealed a pseudo-octahedral geometry, with a pair of Cu^I^ ions occupying each vertex (Figure 1c). Four faces of the octahedron are occupied by tritopic ligands, while the remaining faces are vacant.^4^ Each dimetallic vertex has the same *P* or *M* helical twist, with the assembly expressing approximate *T* point symmetry, consistent with the solution NMR spectra. Both cage enantiomers were observed in the crystal.^17^

The bimetallic vertices display a head-to-head configuration, rather than adopting the head-to-tail arrangement observed in other structures incorporating **A**(15) and related dicopper(I) complexes^18^ (Figure 1b and d). The internal Cu^I^ ion of each vertex is thus chelated by two pyridyl-imine units, and the outer Cu^I^ ions are coordinated by two phosphine donors with a further two external acetonitrile molecules completing their tetrahedral coordination spheres. We infer that this arrangement is more favorable than a counterfactual structure with head-to-tail vertices, where the additional acetonitrile ligands would be left inside the cavity to engender steric crowding (see Supporting Information Section 3).

The coordination environments of the inner Cu^I^ ions are distorted from a regular tetrahedral geometry, with angles of 66.2–70.4° between the two pyridyl-imine chelate planes and N–Cu^I^–N angles in the range 80.3–139.5°. The outer Cu^I^ ions display a more regular tetrahedral geometry, with angles of 97.9–116.9° between ligands. The metal centers of each vertex are separated by 4.02–4.18 Å (average = 4.10 Å), which is much greater than twice the copper(I) van der Waals radius of 1.40 Å,^20^ indicating the absence of Cu^I^···Cu^I^ interactions.

The inner Cu^I^ ions form a regular octahedral framework with an average distance of 12.0 Å along the edges and 16.9 Å between antipodal Cu^I^ ions. The cavity of **1** encapsulates a single acetonitrile molecule in the solid state. Its volume was calculated to be 90 Å^3^ using Molovol.^21^

We reasoned that silver(I) might also form pseudo-octahedral assemblies analogous to **1**, as Ag^I^ and Cu^I^ have similar coordination preferences.^22^ Furthermore, ^109^Ag NMR spectroscopy^23^ provides a complementary means to characterize coordination complexes incorporating diamagnetic Ag^I^ in solution.^24^ Silver(I) complex **2** was thus formed by treating triamine **B** (4 equiv) with **A** (12 equiv) and Ag^I^OTf (12 equiv) (Figure 1a). Its Ag^I^_12_L_4_ composition was confirmed by ESI-MS, and its ^1^H NMR spectrum (Figure 2c) was again consistent with a high-symmetry structure in solution.

**Figure 2:**
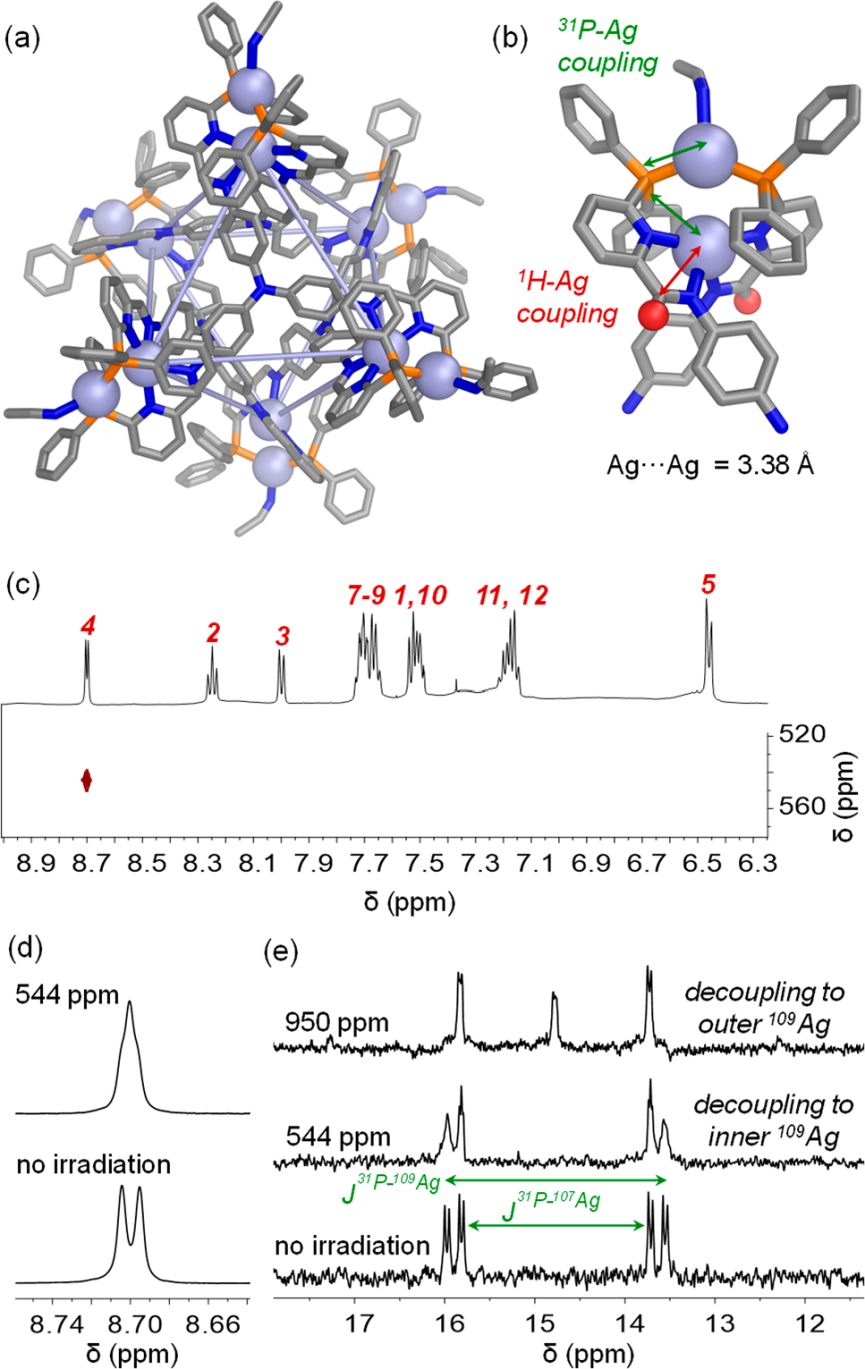
(a) Crystal structure of **2**. Disorder, anions, solvent of crystallization, and hydrogen atoms are omitted for clarity. (b) View of one of the disilver vertices of **2**, with the imine hydrogens shown as red spheres. The observed ^1^H–Ag and ^31^P–Ag couplings are highlighted by red and green arrows, respectively. (c) ^1^H–^109^Ag HMBC of **2**, revealing a correlation between the imine resonance and the inner silver ions, which resonate at 544 ppm. (d) Coalescence of the imine signal in the ^1^H NMR spectrum of **2** upon irradiation of ^109^Ag at 544 ppm. (e) ^31^P NMR spectra of **2** before and after irradiation of ^109^Ag at 544 or 950 ppm, resulting in decoupling to the inner and outer ^109^Ag, respectively.

The crystal structure of **2** confirmed the presence of a pseudo-octahedral assembly (Figure 2a), analogous to **1**, this time with crystallographic *T*-symmetry. The metal–metal separation at each disilver(I) vertex was found to be 3.38 Å, significantly shorter than the average metal–metal distance of 4.10 Å observed for **1** and slightly greater than twice the van der Waals radius of Ag^I^ (1.66 Å).^20^ The inner Ag^I^ ions form a perfect octahedron with 12.2 Å edges and a distance of 17.3 Å between opposing vertices. The cavity of 69 Å^3^ (calculated with Molovol^21^) is slightly smaller than that of **1**, reflecting a more compressed structure.

The inner Ag^I^ ions, once more coordinated by two pyridyl-imine units, are even more distorted from regular tetrahedral geometry (62.6° between pyridyl-imine chelate planes and N–Ag^I^–N angles of 71.1–157.8°) relative to the inner Cu^I^ ions of **1**, consistent with the greater flexibility of the coordination sphere of silver(I).^25^ The outer Ag^I^ ion of each vertex is coordinated by a single acetonitrile molecule in an approximately trigonal planar coordination geometry (Figure 2b). The coordinated acetonitriles were not observed by ^1^H NMR, presumably due to rapid exchange with CD_3_CN.

The solution structure of **2** was further probed through multinuclear NMR experiments (Figure 2c–e), which confirmed the presence of two distinct Ag^I^ environments, corresponding to the inner and outer silver ions at each vertex. These data indicate that the solution structure mirrors the solid-state one. The imine signal in the ^1^H NMR spectrum of **2** split into a doublet (Figure 2d), in contrast to the singlet observed for **1**. In the case of **2**, coupling arises between the imine proton and the nearby internal Ag^I^ ion with a ^109^Ag chemical shift of 544 pm, as determined from a ^1^H–^109^Ag HMBC spectrum (Figure 2c).^26^

The ^31^P NMR spectra of **2** (Figure 2e) showed complex splitting patterns, consistent with coupling between the phosphine and both unique Ag^I^ ions. A major coupling was observed to the external Ag^I^ ions, with further fine splitting resulting from longer-range coupling to the internal Ag^I^ ion, which partially collapsed upon irradiation of the inner ^109^Ag resonance at 544 ppm. Stimulation of ^109^Ag over a broad window in approximately 50 ppm increments (Figure S25) allowed identification of a resonance at ca. 950 ppm, corresponding to the outer Ag^I^ ions.

Because structures **1** and **2** possess two distinct coordination environments, we hypothesized that subcomponent **A** might also be capable of stabilizing assemblies with heterobimetallic vertices. We initially investigated whether Cu^I^ and Ag^I^ could be selectively incorporated into the two distinct binding sites at the vertices of the pseudo-octahedral framework shared by **1** and **2**. However, the reaction of trianiline **B** (4 equiv) and **A** (12 equiv) with equimolar amounts of [Cu^I^(MeCN)_4_](OTf) and Ag^I^OTf (6 equiv each) led to the formation of a distribution of Cu^I^_*x*_Ag^I^_(12–*x*)_L_4_ pseudo-octahedral species (Figure S26). We infer that the similarity in coordinative preferences between Cu^I^ and Ag^I^ led to the formation of these mixed-metal species.

We hypothesized that a metal ion with different coordinative preferences, such as cadmium(II), would lead to discrimination between the different binding sites when combined with copper(I). The self-assembly of triamine **B** (4 equiv) and **A** (12 equiv) with [Cu^I^(MeCN)_4_](ClO_4_) (4 equiv) and Cd^II^(ClO_4_)_2_ (4 equiv) gave rise to a new product (**3**), which displayed a single ^1^H NMR signal for each type of ligand proton (Figure 3a).^27^ ESI-MS revealed a Cd^II^_4_Cu^I^_4_L_4_ composition, distinct from pseudo-octahedral assemblies **1** and **2**.

**Figure 3:**
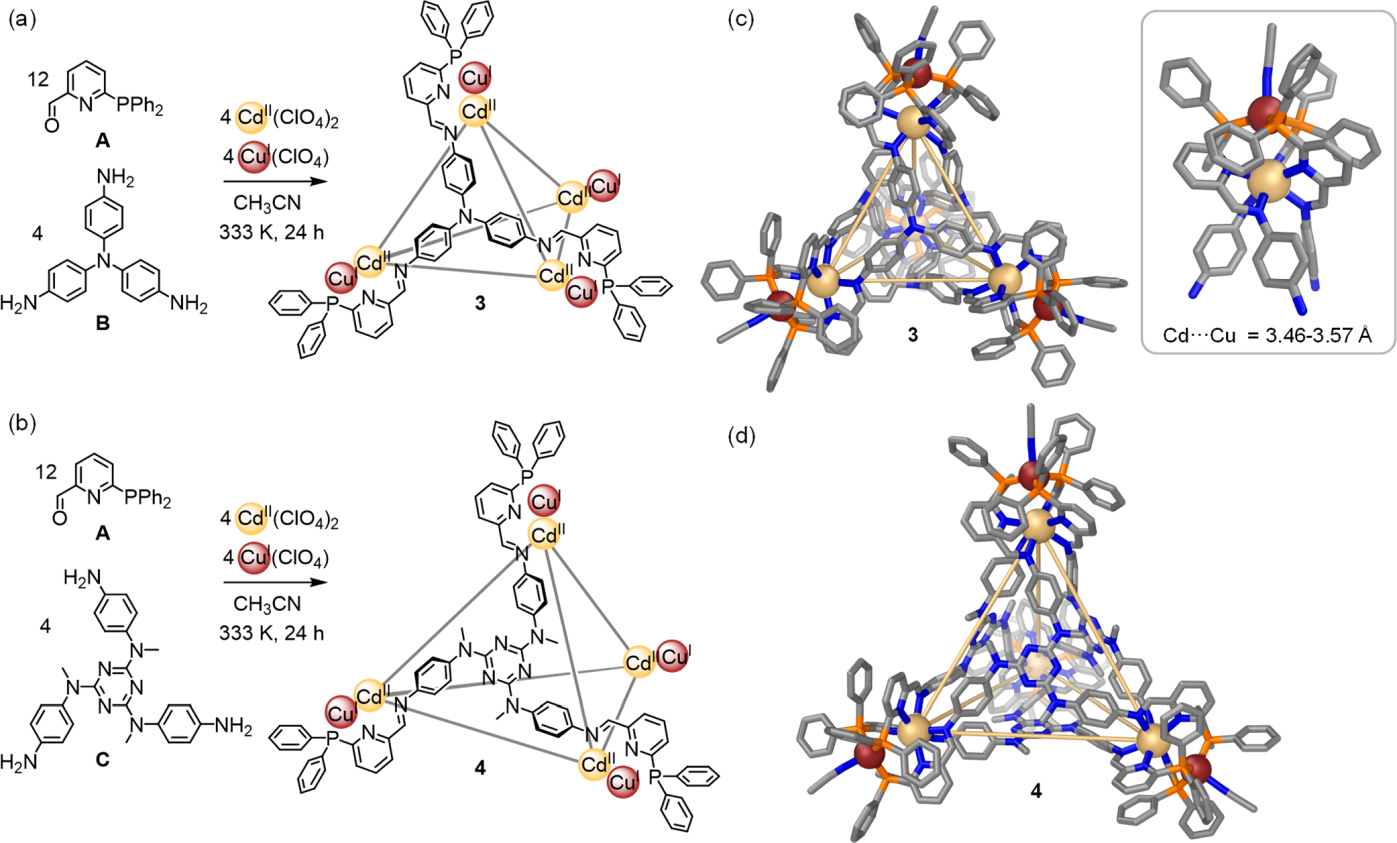
Subcomponent self-assembly of Cd^II^_4_Cu^I^_4_L_4_ cages (a) **3** and (b) **4**. Externally coordinated acetonitrile molecules are omitted for clarity. (c) Crystal structure of **3** with inset showing one Cd^II^Cu^I^ vertex. (d) Crystal structure of **4**. Disorder, anions, solvent of crystallization, and hydrogen atoms are omitted for clarity.

Single-crystal X-ray analysis confirmed the face-capped tetrahedral structure of **3** (Figure 3c). The heterobimetallic vertices of **3** each consist of an inner Cd^II^ and an outer Cu^I^, separated by distances of 3.47–3.57 Å (average 3.52 Å), greater than the sum of the van der Waals radii of the two ions (2.98 Å).^20^ This vertex geometry enables aromatic stacking to occur between a phosphorus-bound phenyl ring from each ligand and the pyridine of a neighboring ligand, with distances of 3.1–3.4 Å between stacked rings (Figure 3c, inset). Such stacking was not observed in the homobimetallic vertices of **1** and **2**.

The Cd^II^ ions bring together three pyridyl-imine ligands at each vertex. The resulting coordination geometry is flattened from a regular octahedral arrangement, with N–Cd^II^–N angles of 71.5–112.0° between *cis*-coordinated nitrogen donors. The Cu^I^ ions are coordinated by a phosphine donor from each ligand, with a single acetonitrile molecule completing the tetrahedral coordination sphere.

Coordination of Cd^II^ to the pyridyl-imine donors within **3** allows them to adopt their preferred six-coordinate configuration, leaving the phosphine donors free to bind Cu^I^ in an approximately tetrahedral configuration. Although both metal ions are classed as soft acids, the lower charge of Cu^I^ renders it softer than Cd^II^, and thus with a greater propensity to coordinate to the softer phosphine donors.^28^

The structure of **3** evokes previously reported M^II^_4_L_4_ tetrahedra,^2a,29^ with all octahedral Cd^II^ ions within each cage sharing the same Δ or Λ stereochemistry, and the face-capping ligands also adopting a propeller-like helical arrangement. The Cd^II^ ions are separated by an average distance of 12.6 Å. A cavity volume of 51 Å^3^ was calculated using Molovol,^21^ within the range observed for analogous tetrahedral cages assembled from **B**, 2-formylpyridine, and Fe^II^ or Co^II^ (31 and 63 Å^3^ respectively, calculated using the same method).^2a,29^ The central nitrogen atoms of each ligand are slightly pyramidalized to point outward, with C–N–C angles ranging from 115.1° to 118.1° (average 117.3°). This observation contrasts with the structures of **1** and **2**, where the central nitrogen atoms are nearly planar, with average C–N–C angles of 119° and 120°, respectively.

To investigate the generality of this approach for forming heterometallic cages, we also prepared a larger tetrahedral cage based on triamine **C**, which was shown to produce M^II^_4_L_4_ tetrahedra with rich host–guest chemistry.^2b^ Treatment of subcomponents **C** (4 equiv) and **A** (12 equiv) with [Cu^I^(MeCN)_4_](ClO_4_) (4 equiv) and Cd^II^(ClO_4_)_2_ (4 equiv) yielded Cd^II^_4_Cu^I^_4_L_4_ structure **4** (Figure 3b), as confirmed by ESI-MS. ^1^H NMR spectra were again consistent with a *T*-symmetric structure in solution.

The crystal structure of **4** confirmed the formation of a face-capped tetrahedral cage with heterobimetallic Cd^II^Cu^I^ vertices, similar to those of **3** (Figure 3d). The internal Cd^II^ cations are separated from one another by an average distance of 16.3 Å, greater than in **3**, and the 240 Å^3^ cavity of **4** is also correspondingly larger, calculated using Molovol.^21^ Future work will compare the guest encapsulation abilities of this cavity with that of the analogous M^II^_4_L_4_ tetrahedron.

Subcomponent **A** represents a rare example of a building block that can generate either homobimetallic or heterobimetallic coordination motifs, resulting in two structurally distinct families of coordination cages. The two chemically distinct coordination environments formed from the previously unreported head-to-head arrangement of **A** have enabled access to cages with heterobimetallic vertices for the first time. Future work will investigate whether the labile coordination sites of the cages, occupied by acetonitrile molecules in the solid state, could enable further functionalization of the cage exterior, to allow tuning of their solubility,^30^ the attachment of fluorescent tags for biomedical applications,^31^ or the chirality of the cages to be controlled.^32^ Explorations may also be fruitful of the mutual influences of the two vertex metal ions, bound at well-defined distances from each other, on the electrochemical properties of the cages and their host–guest properties. Future studies will also seek to exploit the potential photophysical properties^33^ of the copper(I)-based cages reported herein for sensing or optoelectronic applications.^34^

## Supporting Information Available

Detailed descriptions of synthetic procedures; characterization of new compounds; spectroscopic data (PDF)

## Accession Codes

CCDC 2153619–2153622 and 2163435 contain the supplementary crystallographic data for this paper. These data can be obtained free of charge via www.ccdc.cam.ac.uk/data_request/cif, or by emailing data_request@ccdc.cam.ac.uk, or by contacting The Cambridge Crystallographic Data Centre, 12 Union Road, Cambridge CB2 1EZ, UK; fax: +44 1223 336033.
