# Supplementary material for: Incorporation of a Phosphino(pyridine) Subcomponent Enables the Formation of Cages with Homobimetallic and Heterobimetallic Vertices
Source: J Am Chem Soc. 2022 May 5;144(19):8467–73. doi: 10.1021/jacs.2c02261 (PMC9121369; doi:10.1021/jacs.2c02261)
Supplement: Supplementary file 1 — ja2c02261_si_001.pdf [file ja2c02261_si_001.pdf]

Supporting Information for:

Incorporation of a phosphino(pyridine) subcomponent enables the formation of cages with homobimetallic and heterobimetallic vertices

John P. Carpenter‡, Tanya K. Ronson‡, Felix J. Rizzuto, Théophile Héliot, Peter Grice, and Jonathan R. Nitschke*

Department of Chemistry, University of Cambridge, Lensfield Road, Cambridge, CB2 1EW, United Kingdom.

Table of Contents

1. Experimental procedures 2

1.1. General 2

1.2. Synthesis and characterization of cage **1** 3

1.3. Synthesis and characterization of cage **2** 12

1.4. Reaction of cages **1** and **2** to form a library of mixed-metal cages 25

1.5. Synthesis and characterization of cage **3** 26

1.6. Synthesis and characterization of cage **4** 34

2. X-ray crystallography 43

3. Molecular modelling 55

4. Volume calculations 56

5. References 57

1. Experimental procedures

1.1. General

Unless otherwise specified, all starting materials were purchased from commercial sources and used as supplied. 2-Formyl-6-diphenylphosphinopyridine[1] (**A**) and N2,N4,N6-tris(4-aminophenyl)-N2,N4,N6-trimethyl-1,3,5-triazine-2,4,6-triamine[2] (**C**) were prepared according to literature procedures.

NMR spectra were recorded using a 400 MHz Avance III HD Smart Probe (routine ^{1}H NMR experiments, DOSY), DCH 500 MHz dual cryoprobe (high-resolution ^{13}C and 2D experiments), DPX S5 500 MHz BB ATM (variable temperature NMR, multinuclear NMR) and 500 MHz TCI-ATM cryo (^{1}H NMR of host-guest species) NMR spectrometers. Chemical shifts (δ) for ^{1}H NMR spectra are reported in parts per million (ppm) and are reported relative to the solvent residual peak. DOSY experiments were performed on a Bruker DRX-400 spectrometer. Maximum gradient strength was 6.57 G/cmA. The standard Bruker pulse program, ledbpgp2s, employing a stimulated echo and longitudinal eddy-current delay (LED) using bipolar gradient pulses for diffusion using 2 spoil gradients was utilized. Rectangular gradients were used with a total duration of 1.5 ms. Gradient recovery delays were 1200 µs. Diffusion times were 50 ms. Individual rows of the S4 quasi-2D diffusion databases were phased and baseline corrected.

Low-resolution electrospray ionization mass spectra (ESI-MS) were obtained on a Micromass Quattro LC mass spectrometer (cone voltage 5-20 V; desolvation temp. 313 K; ionization temp. 313 K) infused from a Harvard Syringe Pump at a rate of 10 μL per minute. High-resolution ESI mass spectra were obtained by the EPSRC UK National Mass Spectrometry Facility at Swansea University using a Thermofisher LTQ Orbitrap XL, on a Waters LCT Premier Mass Spectrometer featuring a Z spray source with electrospray ionization and modular LockSpray interface or on a Waters Synapt G2-Si instrument.

1.2. Synthesis and characterization of cage 1

Cage **1** was prepared as both the triflate and tetrafluoroborate salts.

Triflate salt:

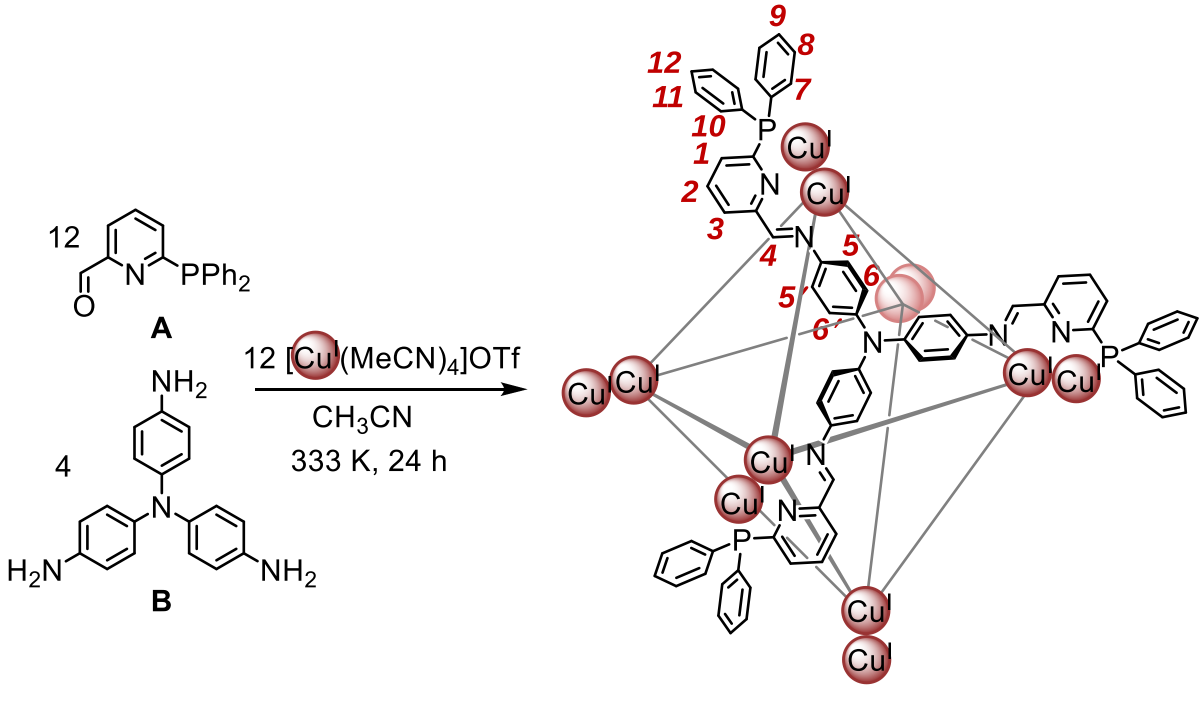

Scheme S1: Subcomponent self-assembly of **1**·12OTf.

Tris(4-aminophenyl)amine **B** (7.74 mg, 27 µmol, 4 equiv.), tetrakis(acetonitrile)copper(I) triflate (30.35 mg, 81 µmol, 12 equiv.) and 2-formyl-6-diphenylphosphinopyridine **A** (23.30 mg, 80 µmol, 12 equiv.) were stirred in CH_3CN (5 mL) at 333 K for 12 h in a sealed vessel under a nitrogen atmosphere, yielding a dark red solution. The reaction mixture was then filtered through a glass fibre filter (0.7 µm pore size) and the solution was concentrated under a stream of N_2 to a volume of 1 mL. Addition of diethyl ether (5 mL) resulted in the precipitation of a dark red powder. The suspension was then centrifuged (10 min, 3000 RPM) and the eluent decanted. Further diethyl ether (5 mL) was added, the powder was resuspended by sonication, centrifuged again and the eluent decanted. The residue was then dried *in vacuo* to afford the solid product as a fine dark red powder (51 mg, 83%).

^{1}H NMR (400 MHz; 298 K; CD_3CN): δ 8.42 (s, 12H, H_4), 8.18 (t, J = 7.9 Hz, 12H, H_2), 7.89 (m, 12H, H_3), 7.89-7.84 (m, 24H, H_7), 7.63 (m, 12H, H_1), 7.62 – 7.54 (m, 36H, H_8 & H_9), 7.30 (m, 12H, H_{10}), 7.04 (t, J = 7.5 Hz, 12H, H_{12}), 6.87 (t, J = 7.6 Hz, 24H, H_{11}), 6.63 (d, J = 8.4 Hz, 24H, H_5 & $H_{5'}$).

Although the signal(s) for the phenyl protons $H_6/H_{6'}$ of **B** were not observed at 298 K due to their intermediate rate of rotation on the NMR timescale, they were resolved when spectra were measured at 238 K, enabling all signals to be assigned (see below). Signals for the bound acetonitrile ligands (which were observed in the solid state) could not be identified in the ^{1}H NMR spectrum due to rapid exchange with CD_3CN.

^{1}H NMR (500 MHz; 223 K; CD_3CN): δ 8.34 (s, 12H, H_4), 8.15 (t, J = 7.8 Hz, 12H, H_2), 7.89 (m, 24H, H_7), 7.81 (d, J = 7.8 Hz, 12H, H_3), 7.63 (d, J = 7.9 Hz, 12H, H_1), 7.61 – 7.52 (m, 36H, H_8 & H_9), 7.12 (d, J = 8.7 Hz, 12H, H_6), 6.99 (t, J = 7.6 Hz, 12H, H_{12}), 6.82 (t, J = 7.6 Hz, 24H, H_{11}), 6.65 (d, J = 8.7 Hz, 12H, H_5), 6.64-6.57 (m, 24H, $H_{5'}$ & $H_{6'}$).

^{31}P NMR (162 MHz, CD_3CN): δ 7.52 (br s).

The limited solubility of **1**·12OTf prevented acquisition of a ^{13}C NMR spectrum within a reasonable timescale. The signals of protonated carbons could be observed in the ^{1}H-^{13}C HSQC spectrum (see Figure S4).

ESI-MS: m/z = 724.7 $[Cu_{12}L_4(OTf)_4]^{8+}$, 849.7 $[Cu_{12}L_4(OTf)_5]^{7+}$, 1016.3 $[Cu_{12}L_4(OTf)_6]^{6+}$, 1249.6 $[Cu_{12}L_4(OTf)_7]^{5+}$, 1599.1 $[Cu_{12}L_4(OTf)_8]^{4+}$.

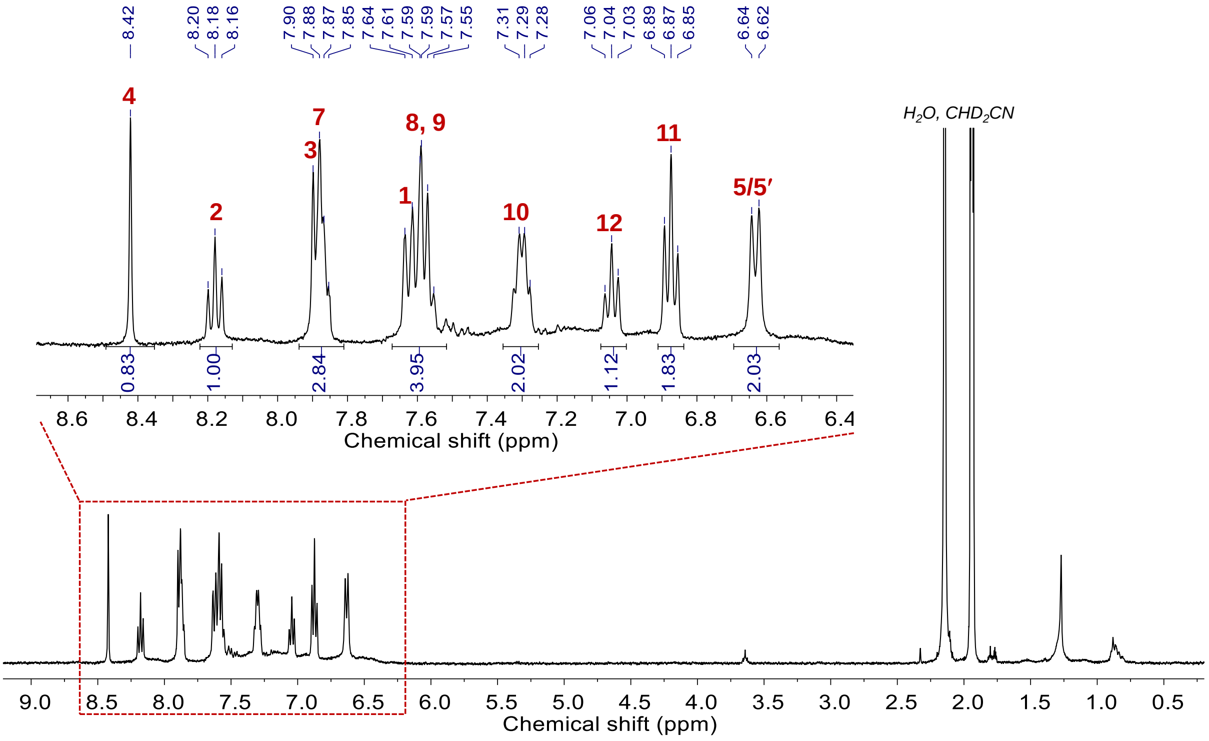

Figure S1: ^{1}H NMR spectrum (500 MHz, 298 K, CD_3CN) of **1**·12OTf with inset showing the aromatic region. The numbering scheme for the proton assignments is shown in Scheme S1. The signal for $H_6/H_{6'}$ was not observed due to dynamic processes occurring in solution.

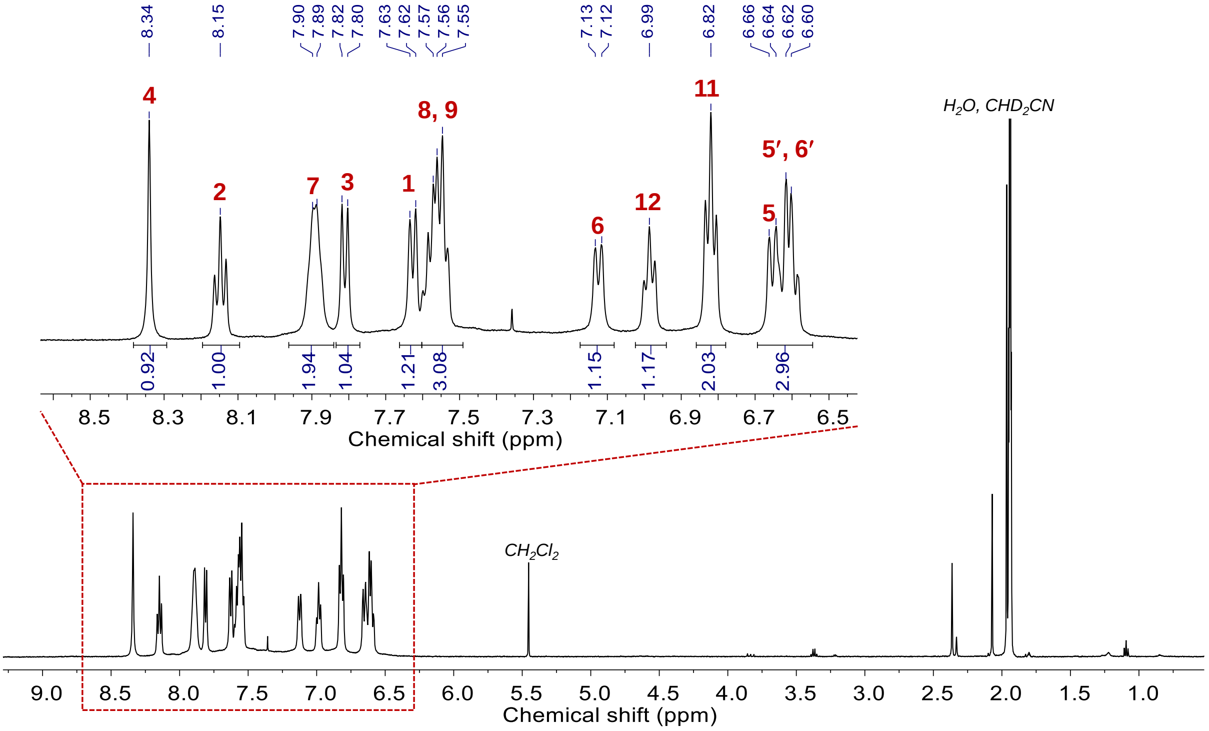

Figure S2: ^{1}H NMR spectrum (500 MHz, 233 K, CD_3CN) of **1**·12OTf with inset showing the aromatic region. The numbering scheme for the proton assignments is shown in Scheme S1. The signal for H_{10} is not observed due to dynamic processes occurring in solution.

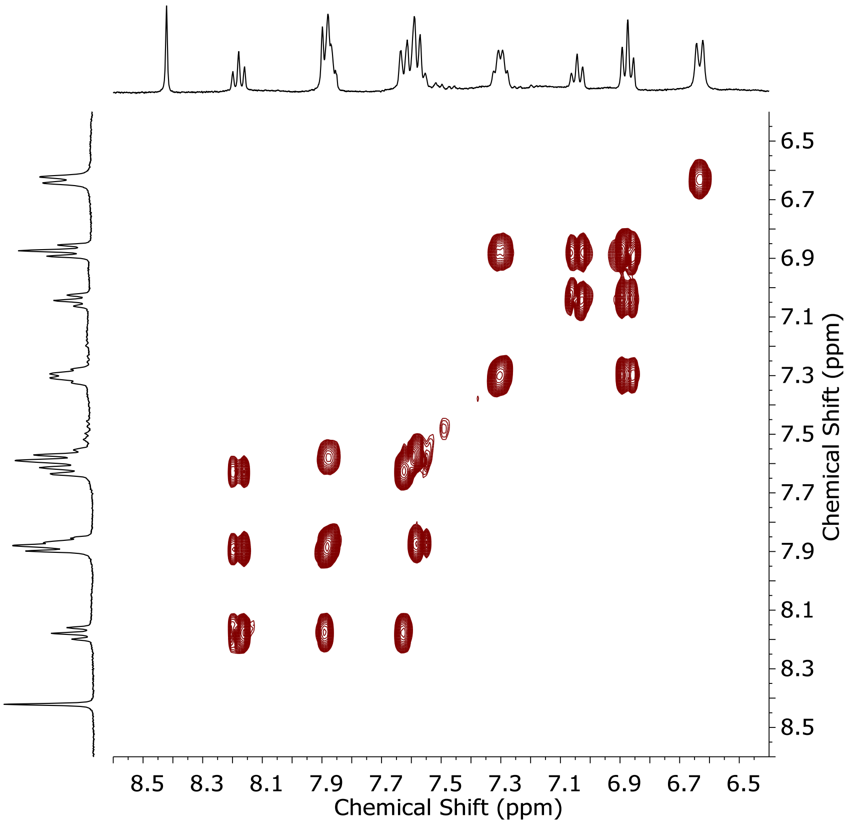

Figure S3: Aromatic region of the ^{1}H-^{1}H COSY spectrum (500 MHz, 298 K, CD_3CN) of **1**·12OTf.

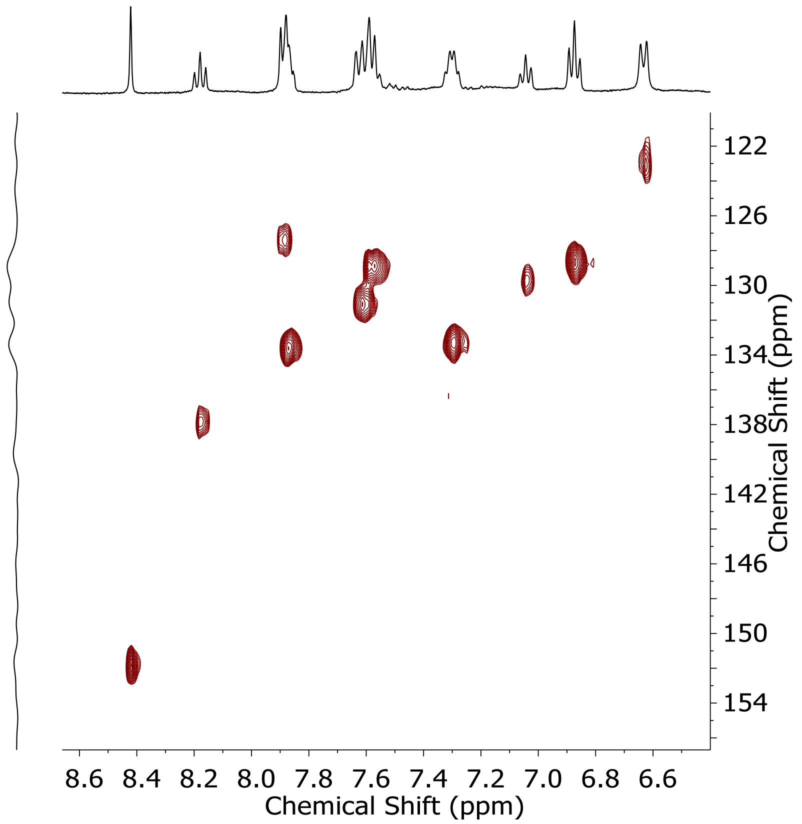

Figure S4: Aromatic region of the ^{1}H-^{13}C HSQC spectrum (500 MHz, 298 K, CD_3CN) of **1**·12OTf.

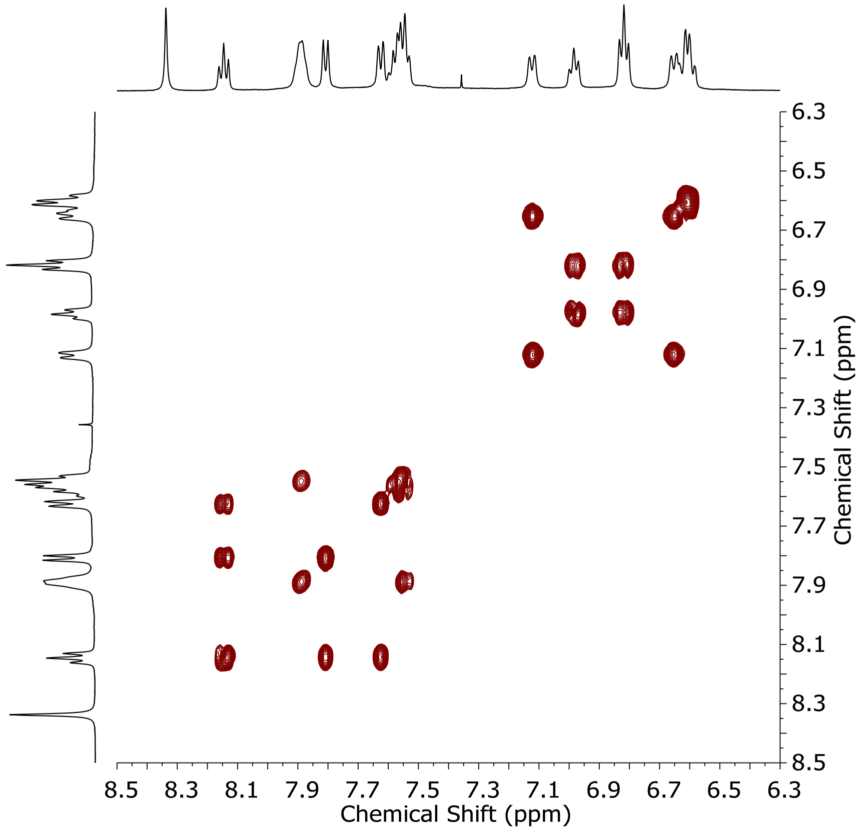

Figure S5: Aromatic region of the ^{1}H-^{1}H COSY spectrum (500 MHz, 233 K, CD_3CN) of **1**·12OTf.

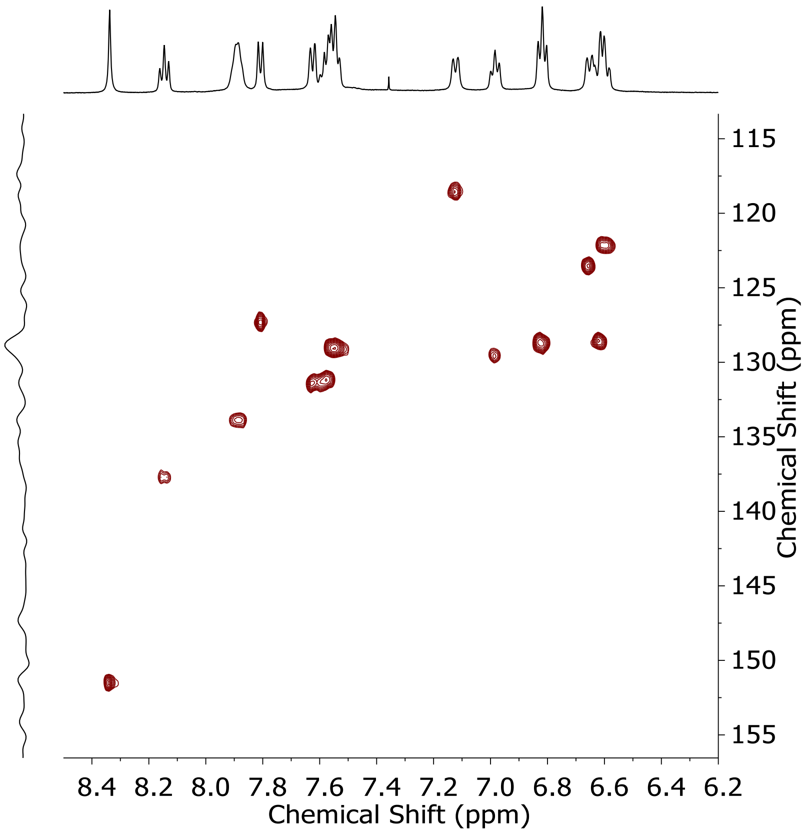

Figure S6: Aromatic region of the ^{1}H-^{13}C HSQC spectrum (500 MHz, 233 K, CD_3CN) of **1**·12OTf.

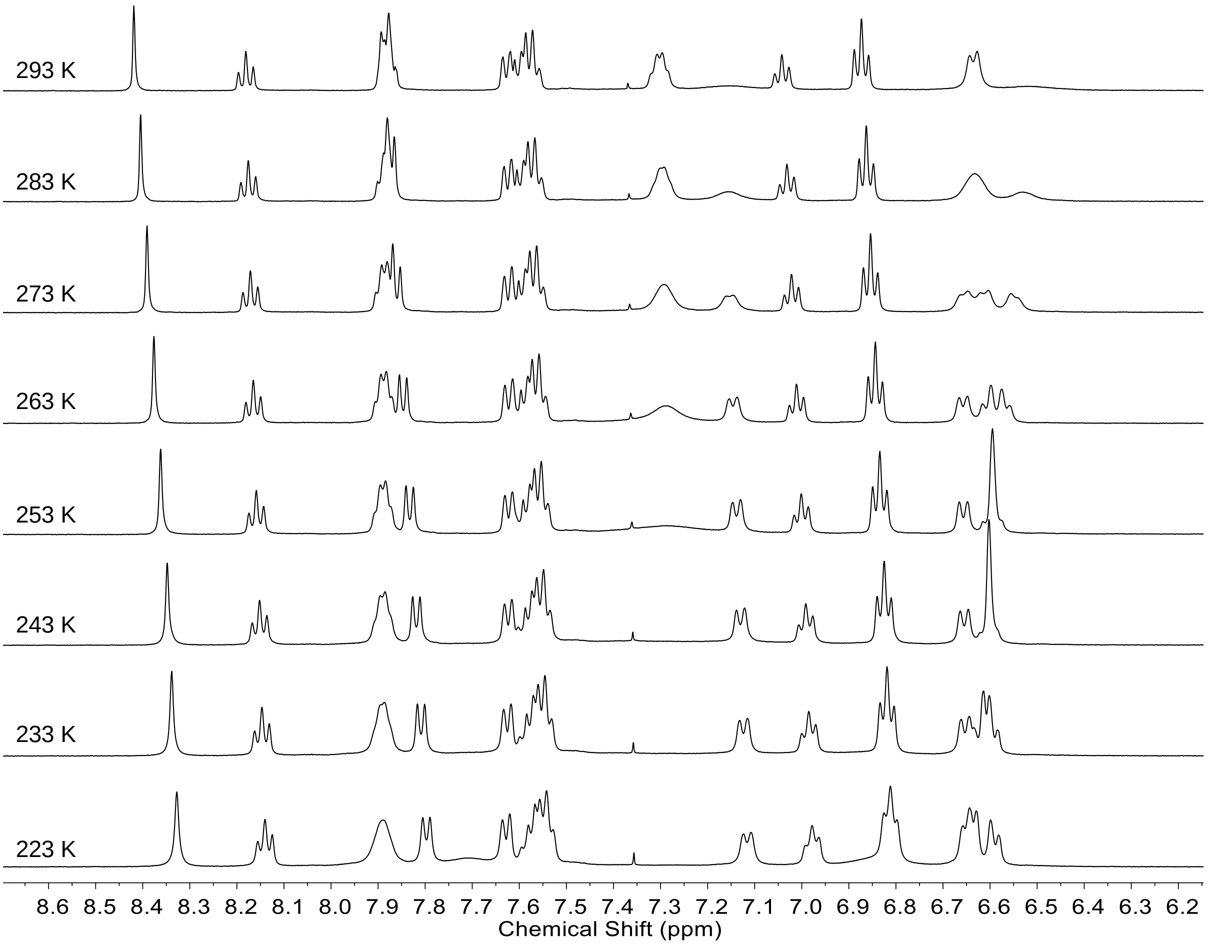

Figure S7: VT-NMR stack plot (500 MHz, CD_3CN) showing the effect of temperature on the ^{1}H NMR spectrum of cage **1**·12OTf.

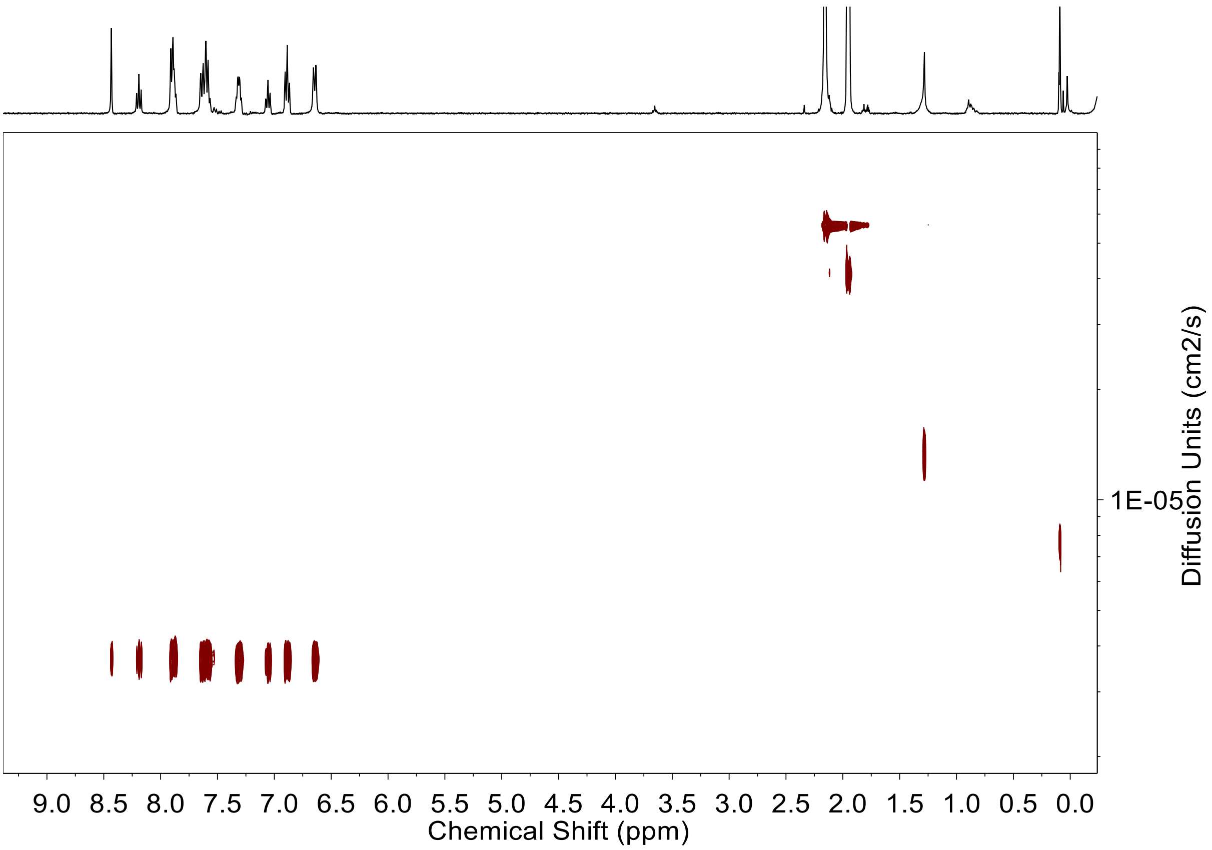

Figure S8: ^{1}H DOSY spectrum (400 MHz, 298 K, CD_3CN) of **1**·12OTf. The diffusion coefficient was measured to be $(3.6 \pm 0.1) \times 10^{-6}$ cm^2 s^{-1}.

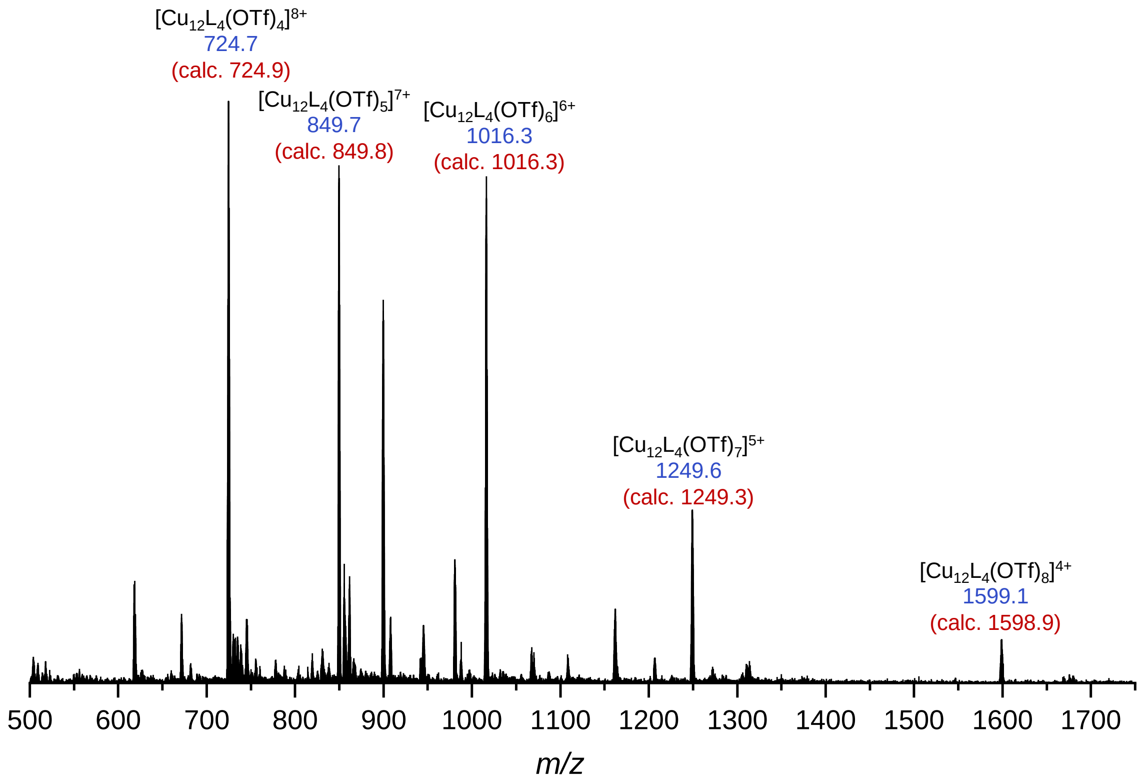

Figure S9: Low-resolution ESI-mass spectrum of **1**·12OTf. Note that the weakly coordinated acetonitrile ligands were not observed under the MS conditions, presumably due to their loss during the ionization process.

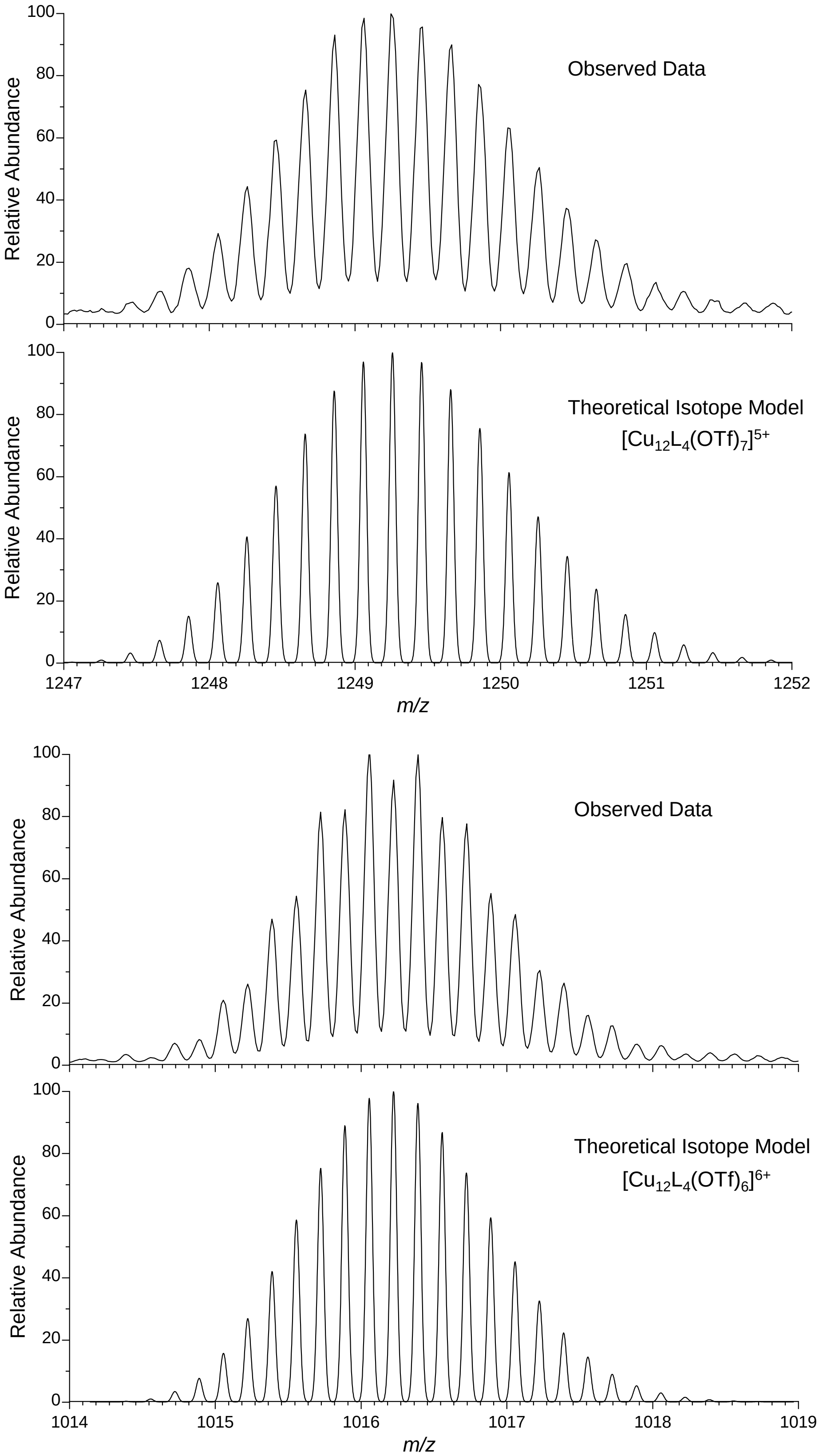

Figure S10: High-resolution ESI-mass spectrometry analysis of **1**·12OTf showing the observed (top) and theoretical (bottom) isotope patterns the +5 and +6 peaks.

Tetrafluoroborate salt:

The tetrafluoroborate salt of **1** was prepared *via* an identical procedure in the case of the triflate salt, except that tetrakis(acetonitrile)copper(I) tetrafluoroborate was used as the metal source. All spectral data were very similar to those obtained for **1**·12OTf.

1.3. Synthesis and characterization of cage 2

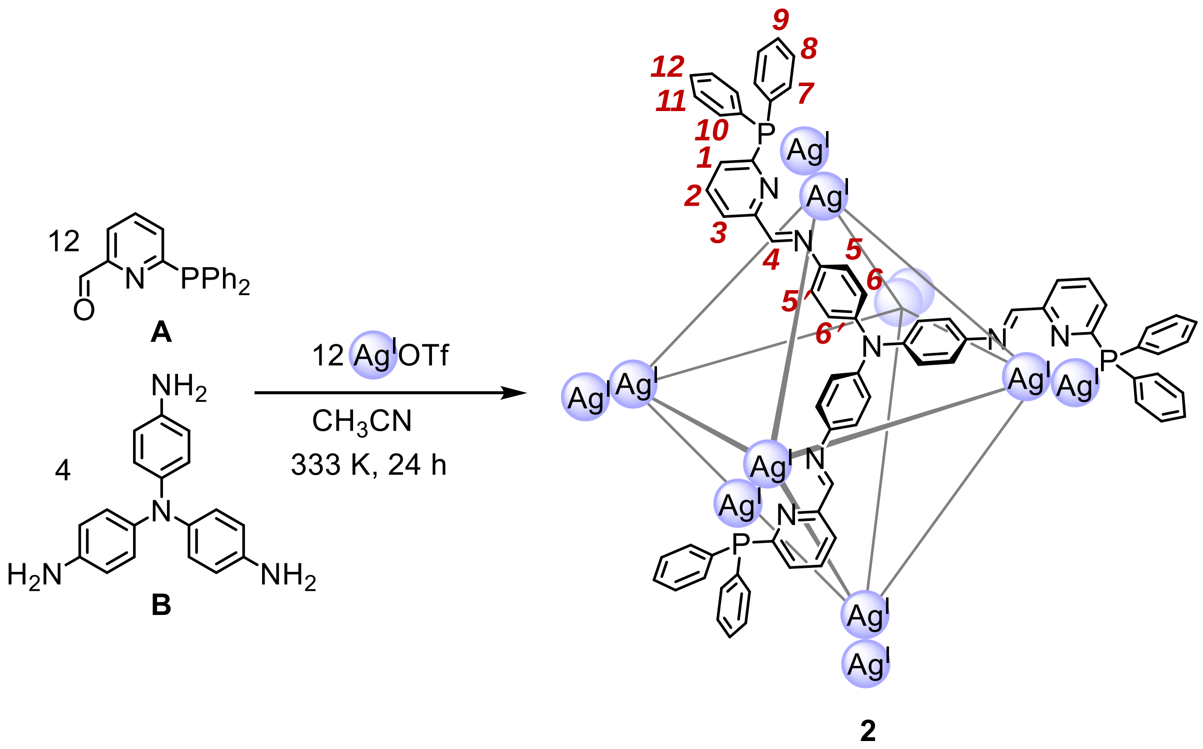

Scheme S2: Subcomponent self-assembly of **2**·12OTf.

Tris(4-aminophenyl)amine **B** (7.76 mg, 27 µmol, 4 equiv.), silver(I) triflate (20.6 mg, 82 µmol, 12 equiv.) and 2-formyl-6-diphenylphosphinopyridine **A** (23.2 mg, 79 µmol, 12 equiv.) were stirred in CD_3CN (5 mL) at 333 K for 12 h in a sealed vessel under a nitrogen atmosphere and in the dark, yielding a light red solution. The reaction mixture was then filtered through a glass fibre filter (0.7 µm pore size) and the solution was concentrated under a stream of N_2 to a volume of 1 mL. Addition of diethyl ether (4 mL) resulted in the precipitation of a dark powder. The suspension was then centrifuged (10 min, 3000 RPM) and the eluent decanted. Further diethyl ether (5 mL) was added, the powder was resuspended by sonication, centrifuged again and the eluent decanted. The residue was then dried *in vacuo* to afford the solid product as a fine bright red powder (45 mg, 89%).

^{1}H NMR (500 MHz; 298 K; CD_3CN): δ 8.70 (d, $J^{1H\text{-}Ag}$ = 4.6 Hz, 12H, H_4), 8.25 (t, 7.8 Hz, 12H, H_2), 8.00 (d, J = 7.8 Hz, 12H, H_3), 7.76 – 7.61 (m, 60H, H_7, H_8 & H_9), 7.57 – 7.46 (m, 36H, H_1 & H_{10}), 7.23-

7.14 (m, 36H, H_{11} & H_{12}), 6.46 (m, 24H, H_5 & $H_{5'}$). Signals for the bound acetonitrile ligands (which were observed in the solid state) could not be identified in the ^{1}H NMR spectrum due to rapid exchange with CD_3CN.

^{1}H NMR (500 MHz; 233 K; CD_3CN): δ 8.63 (d, $J^{1H\text{-}Ag}$ = 4.5 Hz, 12H, H_4), 8.22 (t, 7.8 Hz, 12H, H_2), 7.91 (d, J = 7.8 Hz, 12H, H_3), 7.77 – 7.57 (m, 60H, H_7, H_8 & H_9), 7.51 – 7.44 (m, 36H, H_1 & H_{10}), 7.18-7.10 (m, 48H, H_6, H_{11} & H_{12}), 6.46 (br s, 12H, H_5), 6.41 (br s, 12H, $H_{5'}$), 5.77 (br s, 12H, $H_{6'}$).

^{13}C NMR (126 MHz, CD_3CN): δ 154.1 (imine), 152.8 (m), 147.8, 141.7, 136.8 (m), 134.9 (m), 133.3, 133.0, 132.2 (m), 131.1-130.8 (multiple overlapping signals), 130.1, 128.6 (m), 124.5, 122.2 (q, $^{1}J_{CF}$ = 320.9 Hz, ($CF_3SO_3^{-}$). Some of the expected peaks are not detected due to a combination of low intensity, coupling to ^{31}P and signal overlap.

^{31}P NMR (162 MHz, CD_3CN): δ 14.78 (multiplet). See Figure S24 for further details.

ESI-MS: m/z = 791.5 $[Ag_{12}L_4(OTf)_4]^{8+}$, 925.7 $[Ag_{12}L_4(OTf)_5]^{7+}$, 1104.8 $[Ag_{12}L_4(OTf)_6]^{6+}$, 1355.6 $[Ag_{12}L_4(OTf)_7]^{5+}$, 1732.0 $[Ag_{12}L_4(OTf)_8]^{4+}$.

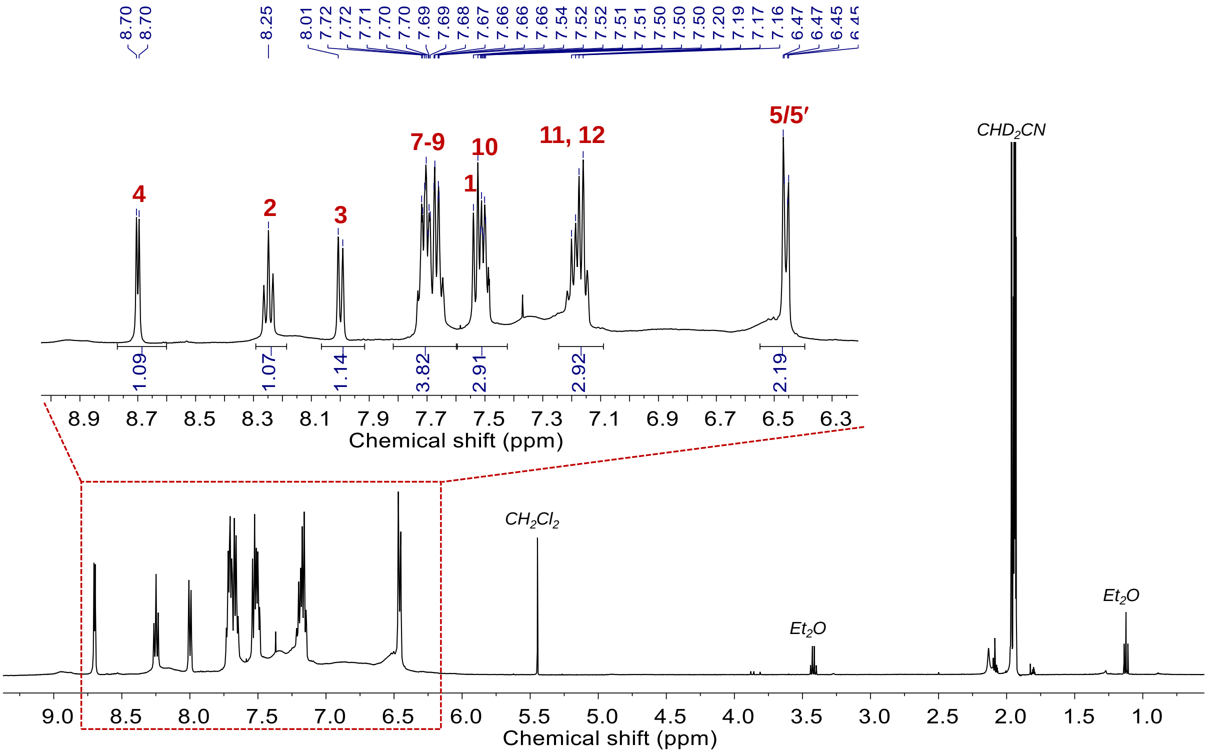

Figure S11: ^{1}H NMR spectrum (500 MHz, 298 K, CD_3CN) of **2**·12OTf with inset showing the aromatic region. The numbering scheme for the proton assignments is shown in Scheme S2. The signal for H_6/$H_{6'}$ is not observed due to dynamic processes occurring in solution.

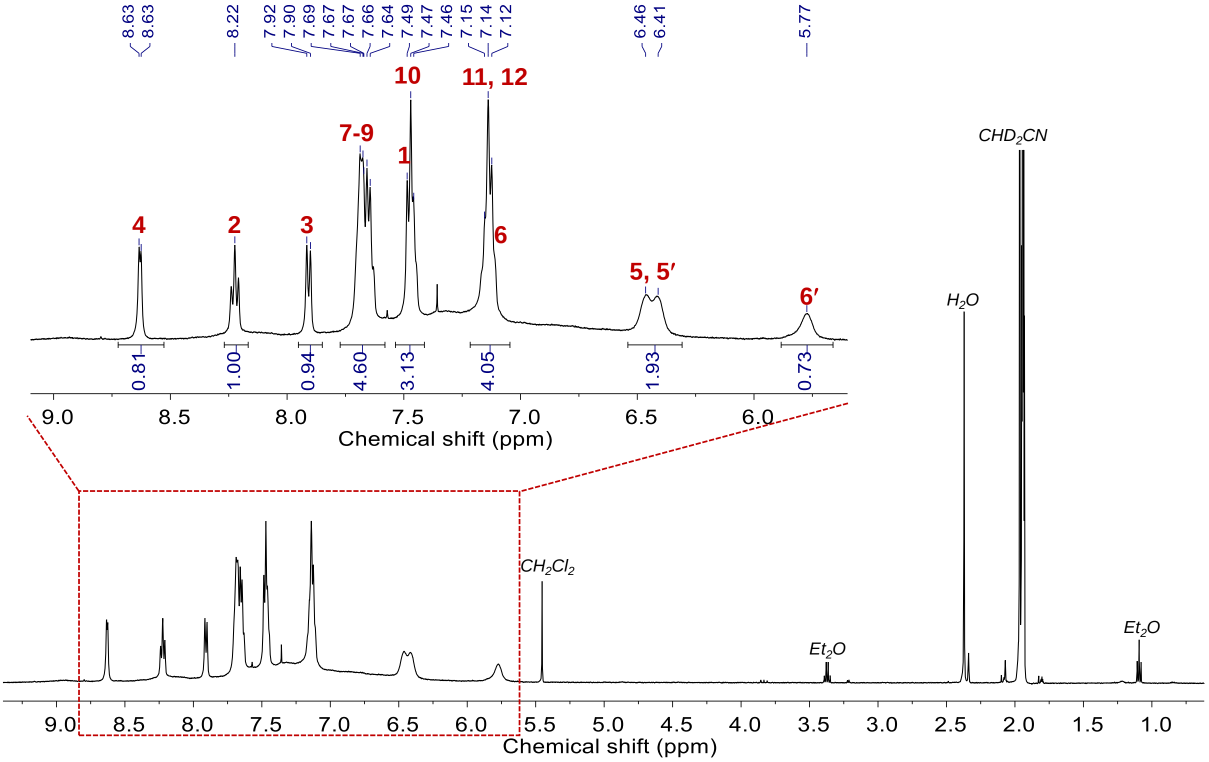

Figure S12: ^{1}H NMR spectrum (500 MHz, 233 K, CD_3CN) of **2**·12OTf with inset showing the aromatic region. The numbering scheme for the proton assignments is shown in Scheme S2.

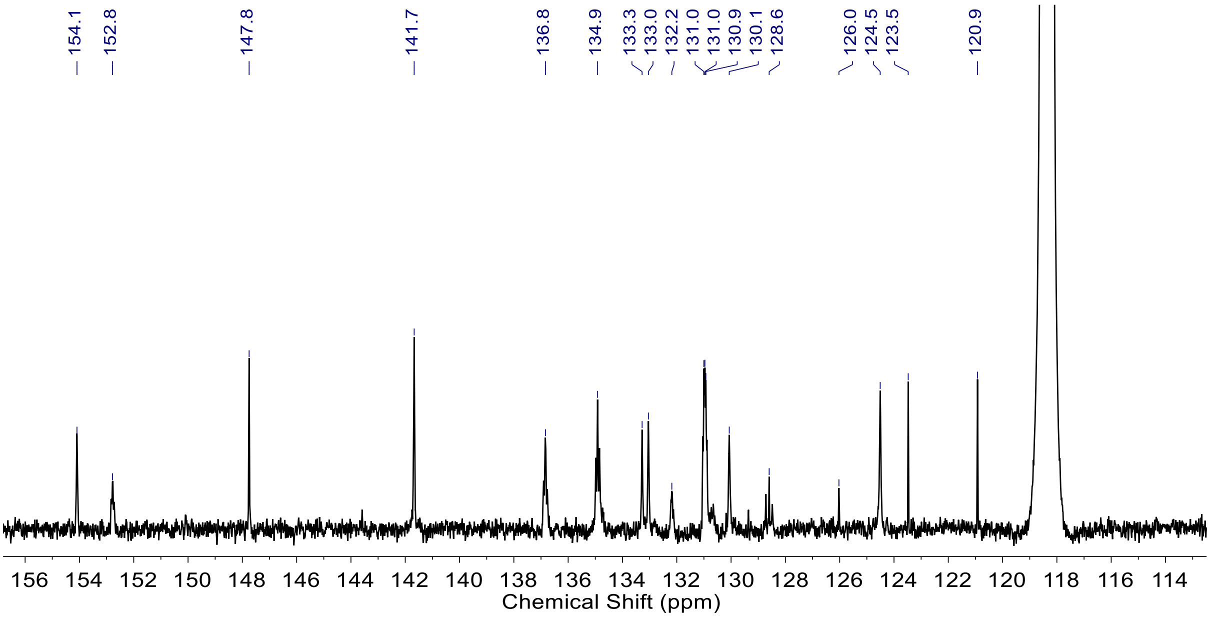

Figure S13: ^{13}C NMR spectrum (126 MHz, 298 K, CD_3CN) of **2**·12OTf.

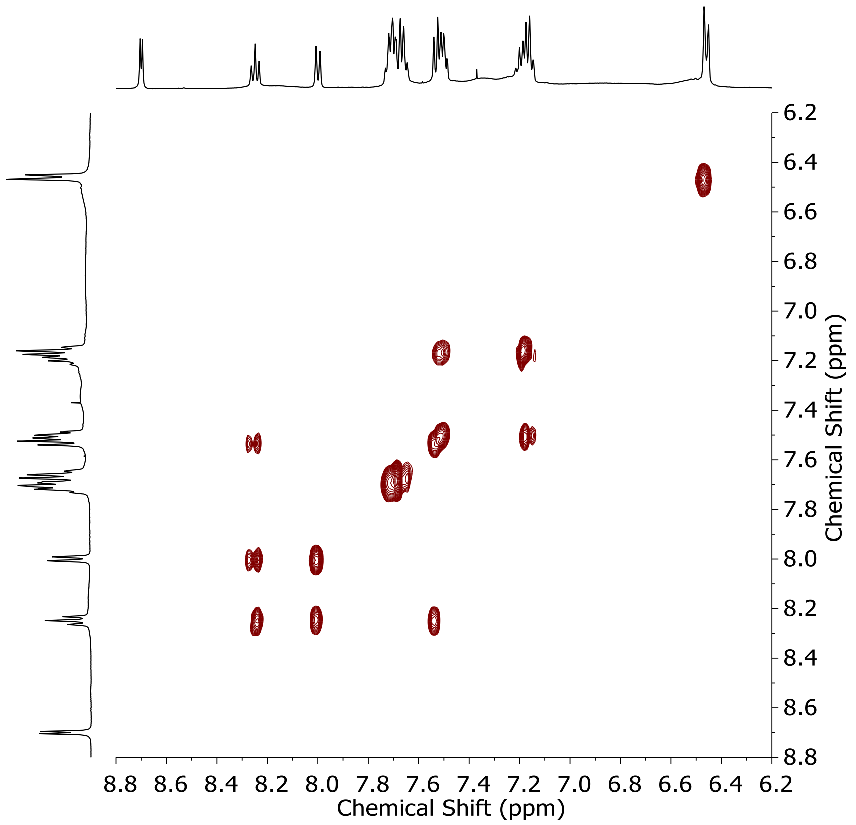

Figure S14: Aromatic region of the ^{1}H-^{1}H COSY spectrum (500 MHz, 298 K, CD_3CN) of **2**·12OTf.

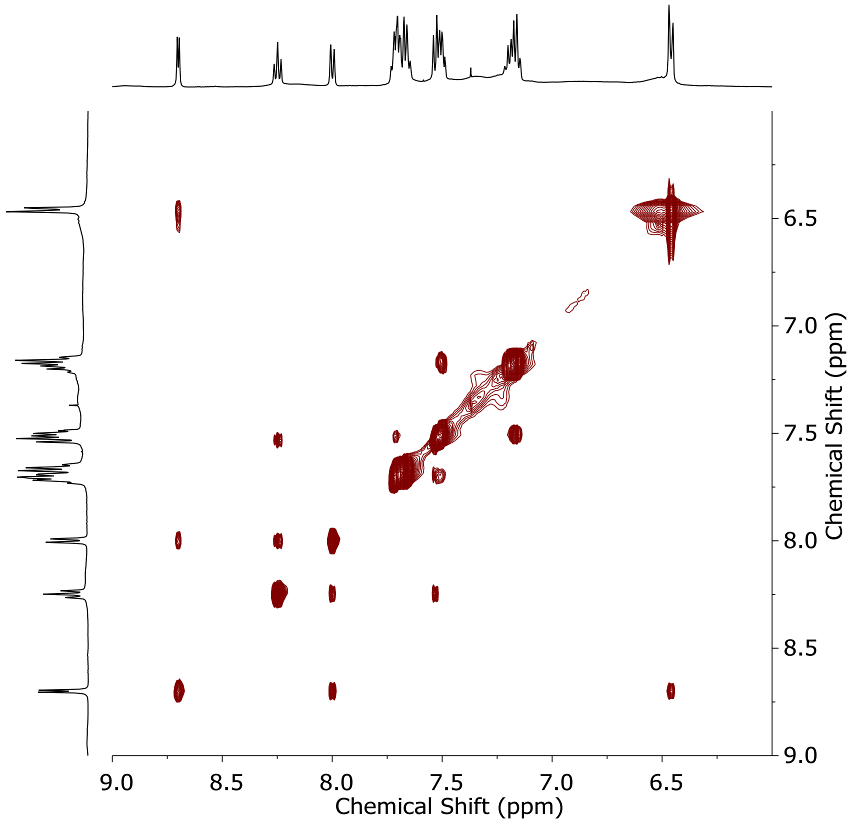

Figure S15: Aromatic region of the ^{1}H-^{1}H NOESY spectrum (500 MHz, 298 K, CD_3CN) of **2**·12OTf.

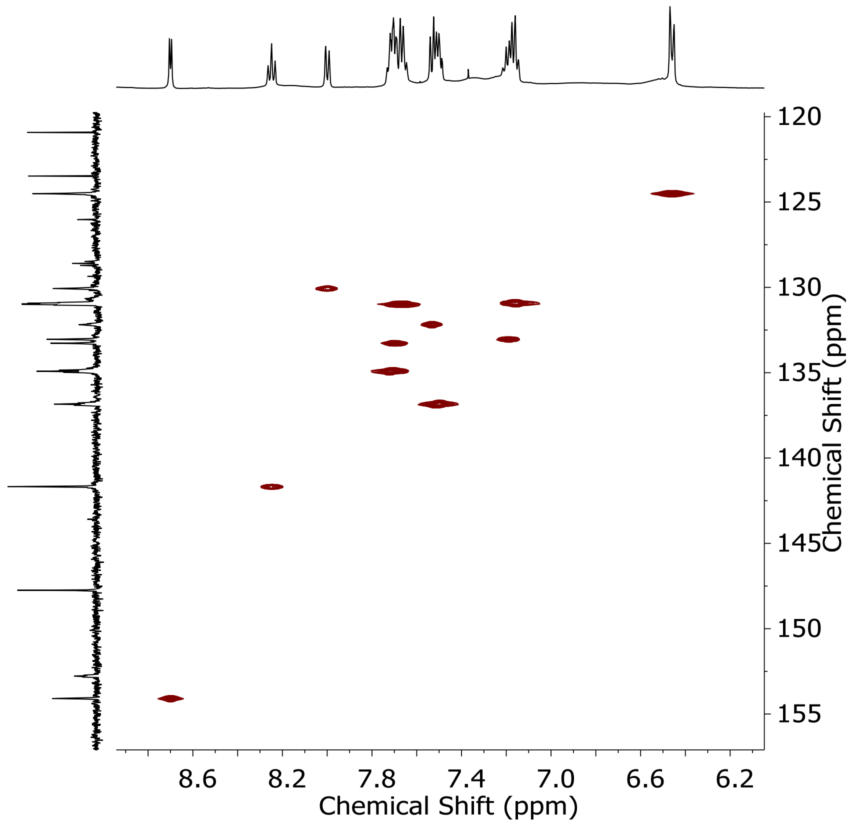

Figure S16: Aromatic region of the ¹H-¹³C HSQC spectrum (500 MHz, 298 K, CD_3CN) of **2**·12OTf.

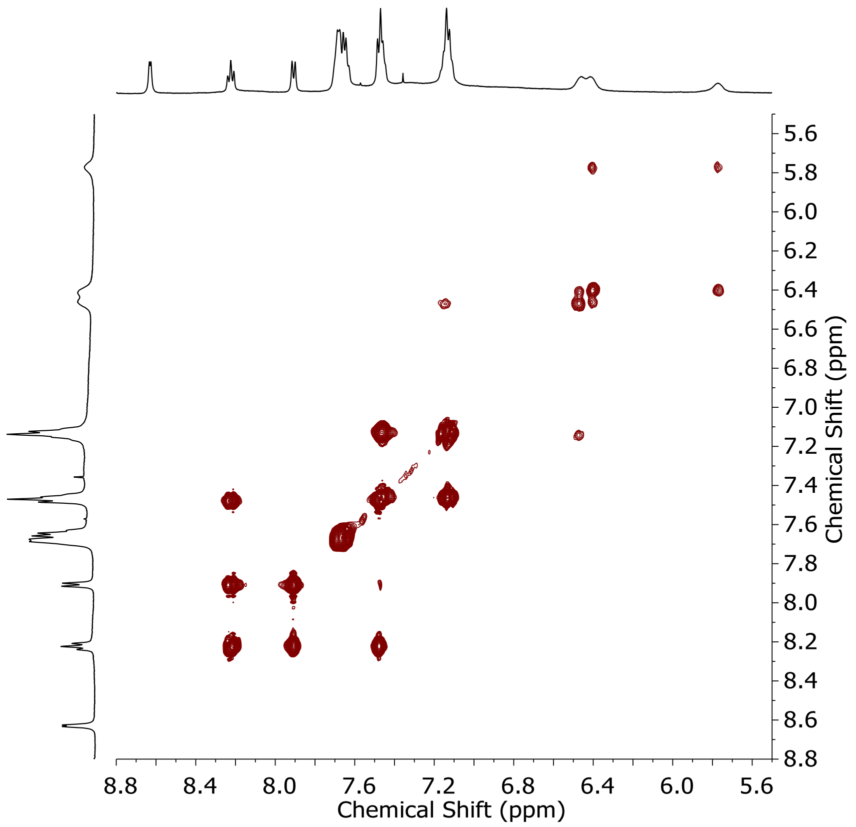

Figure S17: Aromatic region of the ¹H-¹H COSY spectrum (500 MHz, 233 K, CD_3CN) of **2**·12OTf.

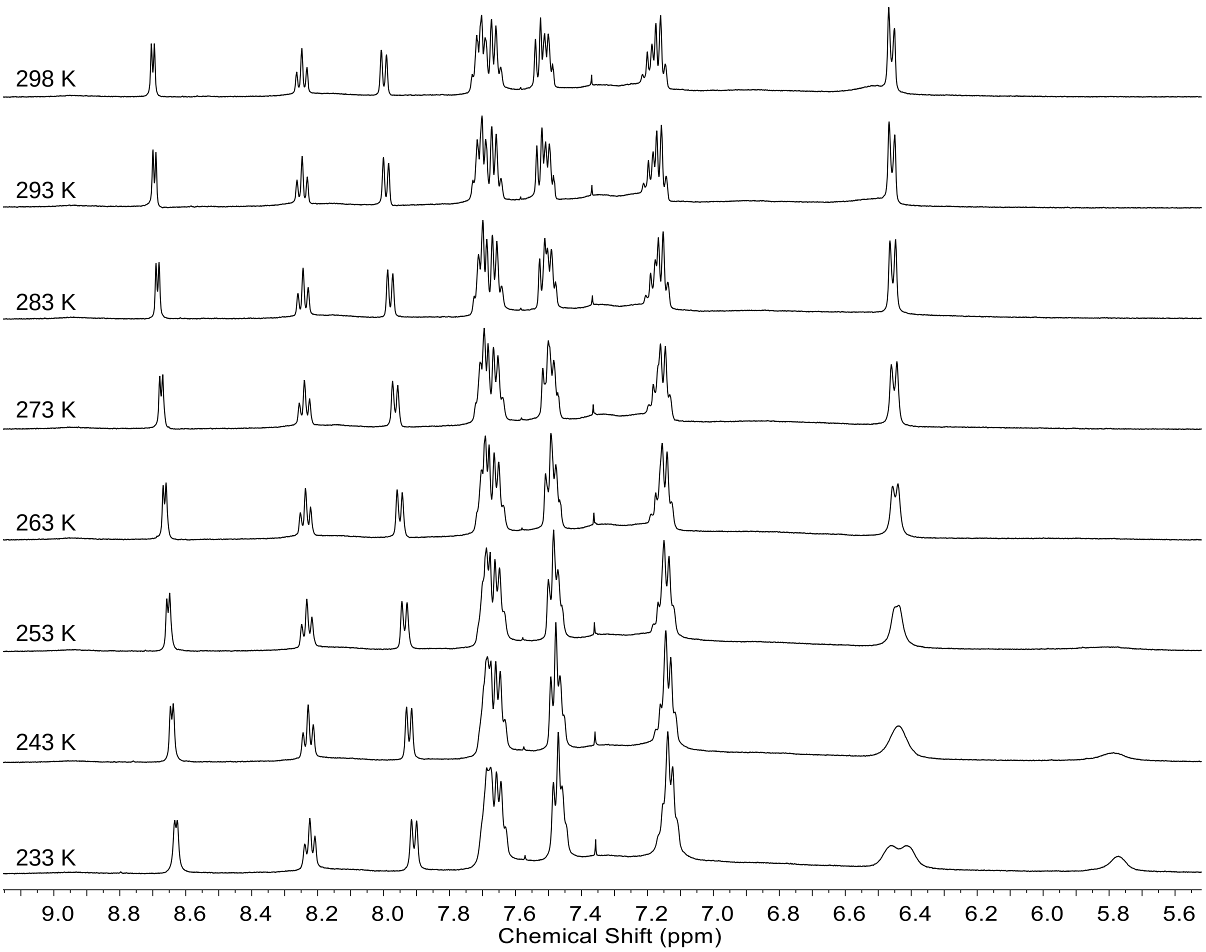

Figure S18: VT-NMR stack plot (500 MHz, CD_3CN) showing the effect of temperature on the ^1H NMR spectrum of cage **2**·12OTf.

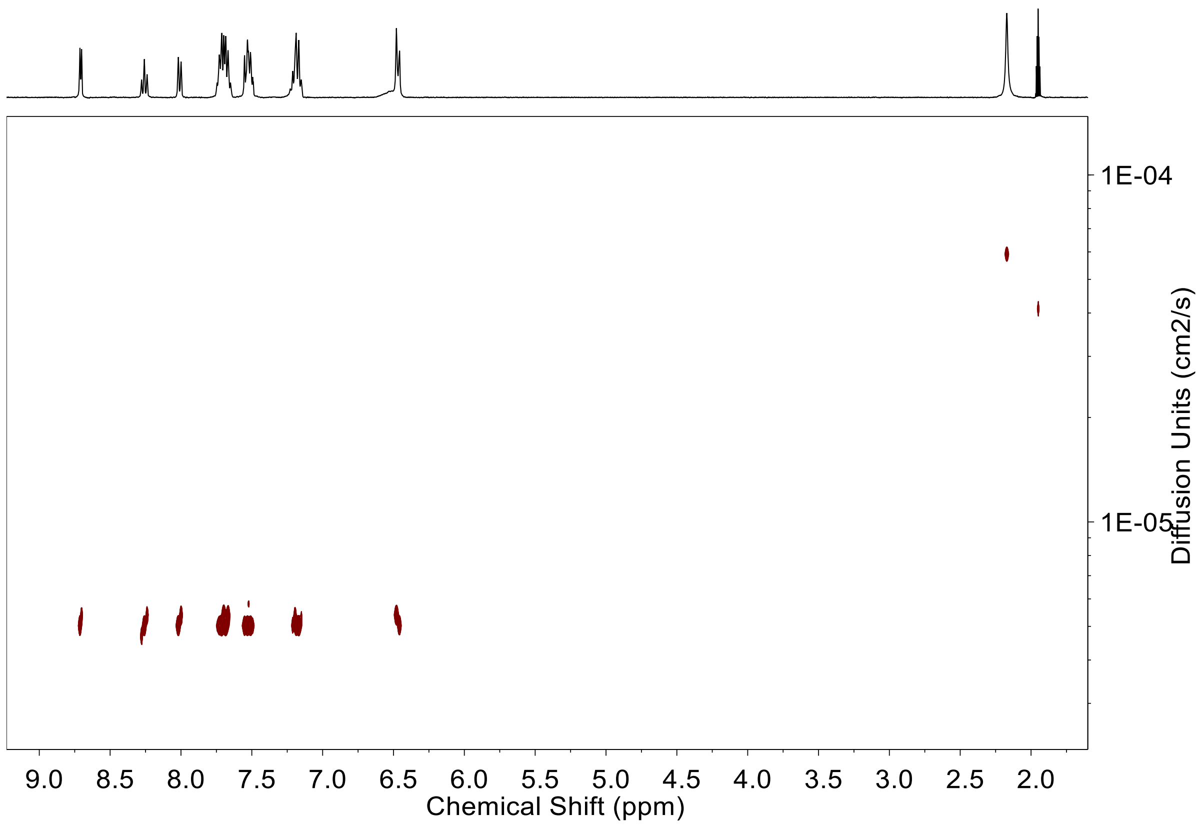

Figure S19: ^{1}H DOSY spectrum (400 MHz, 298 K, CD_3CN) of **2**·12OTf. The diffusion coefficient was measured to be (5.0 ± 0.1) × 10^{-6} cm^2 s^{-1}.

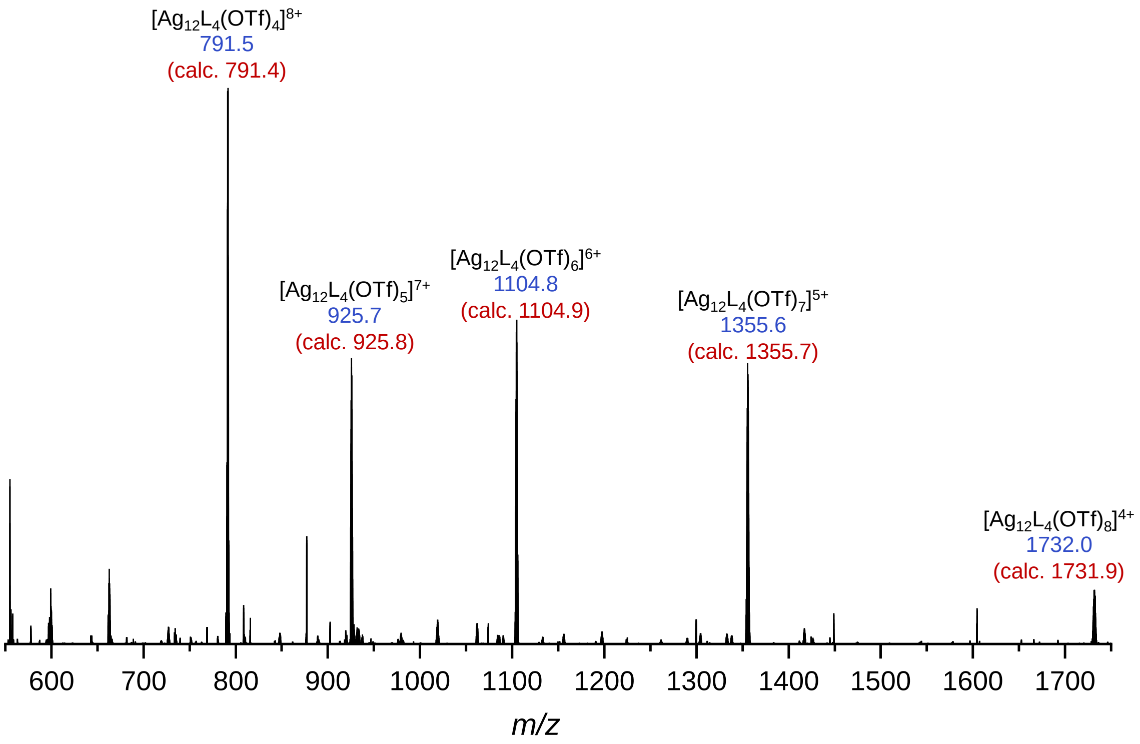

Figure S20: ESI-mass spectrum of **2**·12OTf. Note that the weakly coordinated acetonitrile ligands were not observed under the MS conditions, presumably due to their loss during the ionization process.

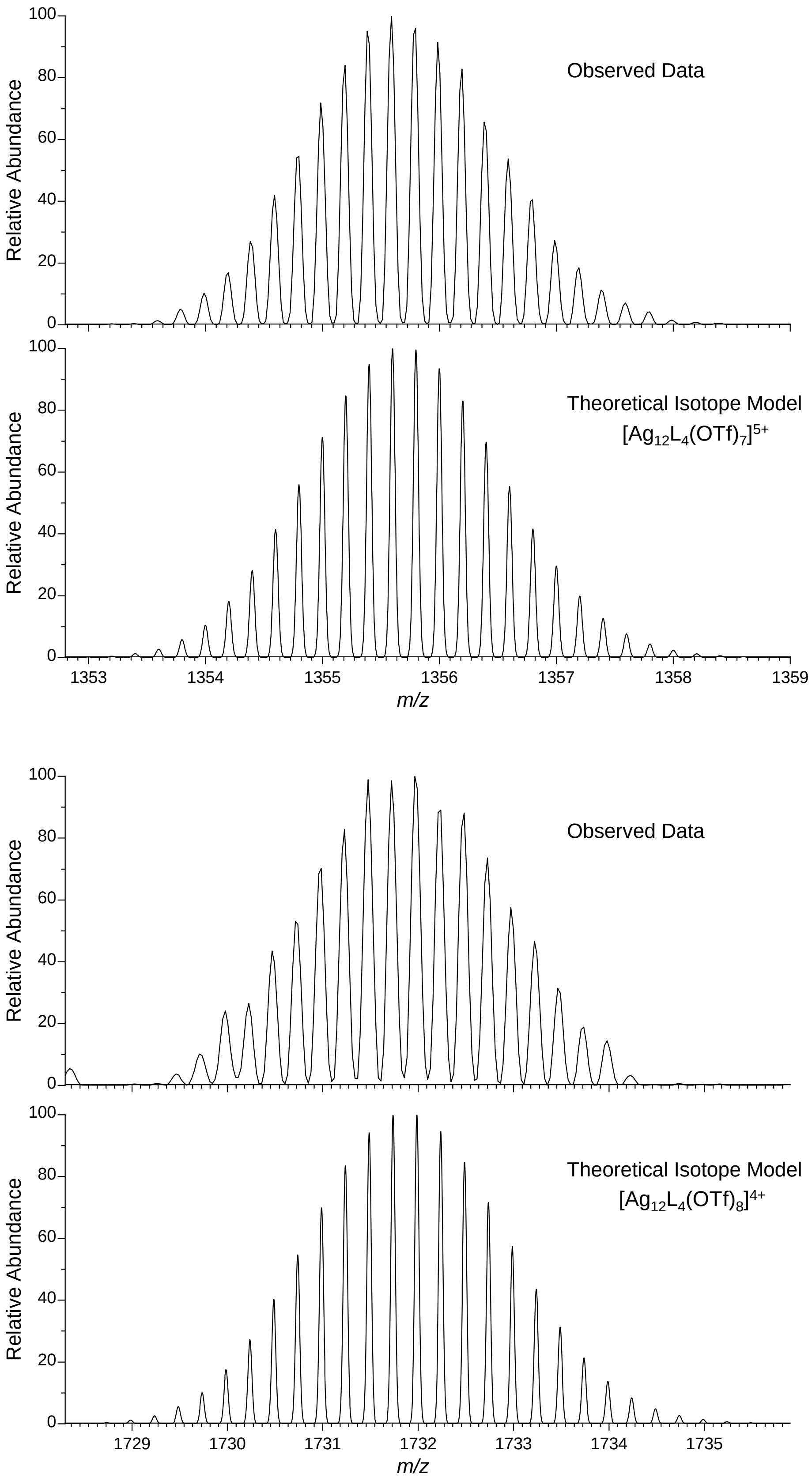

Figure S21: High-resolution ESI-mass spectrometry analysis of **2**·12OTf showing the observed (top) and theoretical (bottom) isotope patterns for the +5 and +4 peaks.

Multinuclear NMR experiments:

Silver(I) is NMR active providing another means to characterize the coordination environment of **2** in solution. Silver mainly exists as two isotopes, ^{107}Ag and ^{109}Ag (approximately 51.8% and 48.2%, respectively), which are both spin ½ and therefore NMR active, of which ^{109}Ag is usually preferred for probing the coordination environment of silver complexes due to its slightly higher sensitivity. If the solution structure of **2** reflects the solid-state structure we would expect two distinct resonances to be observed in the ^{109}Ag NMR spectrum, corresponding to the inner and outer silver ions at each vertex. However, the limited sensitivity of this isotope combined with the limited solubility of **2** prevented direct acquisition of the ^{109}Ag NMR spectrum within a reasonable timescale. To circumvent this limitation, a series of ^{1}H and ^{31}P NMR experiments were devised to investigate the effects of the coupling between the protons and phosphorus atoms of **2** and its Ag^{I} cations.

The imine signal in the ^{1}H NMR spectrum of **2** is split into a doublet, arising from coupling to the nearby Ag^{I} cation (Figure S23, bottom). The ^{1}H-^{109}Ag HMBC spectrum (Figure S22) reveals coupling between this proton signal and a Ag^{I} species with a chemical shift of 544 ppm, which we infer to correspond to the internal Ag^{I} ions of the cage. Irradiation of ^{109}Ag at 544 ppm results in coalescence of the imine signal in the ^{1}H NMR spectrum of **2** (Figure S23, top), further supporting this assignment. Several couplings are observed in the ^{31}P NMR spectrum of **2**, again consistent with coupling to Ag^{I} (Figure S24, bottom). The splitting pattern is too complex to be entirely explained by coupling with just the most external Ag^{I} ion with further fine splitting of the signals observed. We infer that this fine splitting in the ^{31}P NMR spectrum results from longer range coupling between the internal Ag^{I} ion and the phosphorus. Irradiation of ^{109}Ag at 544 ppm resulted in significant changes in the acquired ^{31}P NMR spectrum, with partial collapse of the fine coupling to ^{109}Ag (Figure S24, top). Coupling resulting from ^{107}Ag is still present.

The relative insensitivity of both ^{109}Ag and ^{31}P prevented further analysis by HMBC. Instead, ^{109}Ag was excited over a relatively broad window in approximately 50 ppm increments from 300–1000 ppm (Figure S25), which allowed identification of a second ^{109}Ag species at ca. 950 ppm. Following irradiation at 950 ppm, one of the major doublets collapses but retains its fine coupling to the internal Ag^{I} ions.

These results, when taken together with the ^{1}H-^{109}Ag HMBC experiment, provide strong evidence that the structure occurring in solution has two distinct Ag^{I} ions. The solution structure of **2** is thus fully consistent with its solid-state structure. A hypothetical structure based on bimetallic vertices with a head-to-tail arrangement (see Section 3 below), which would also be of high symmetry by ^{1}H NMR, can be ruled out as the two metal centers comprising each vertex would also be chemically equivalent in this case.

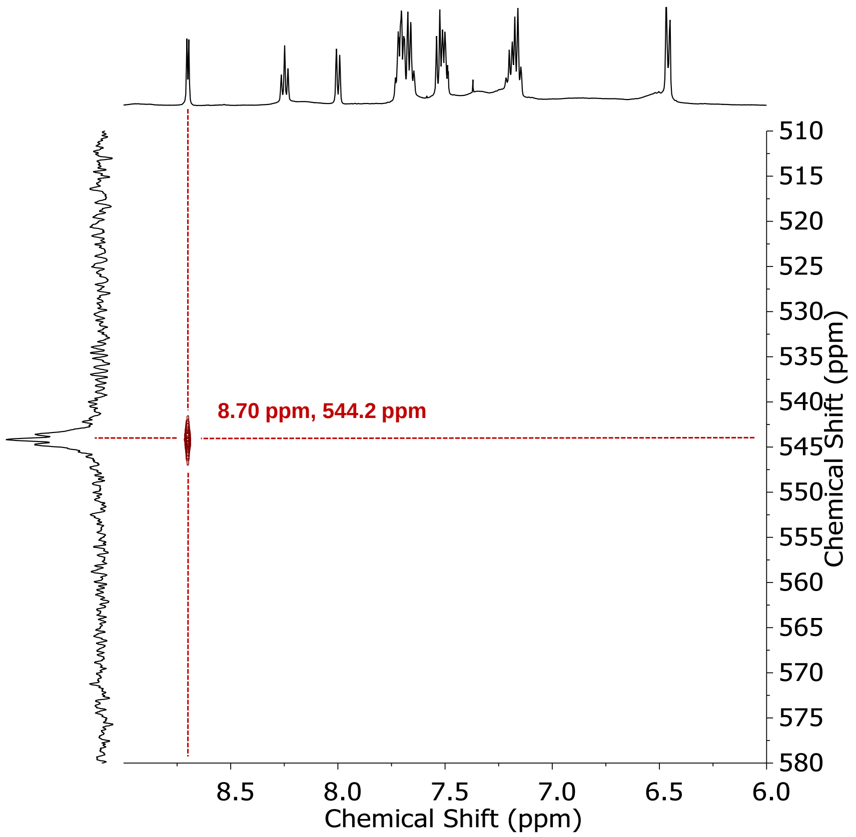

Figure S22: ^{1}H–^{109}Ag HMBC of **2**, revealing a strong correlation between the imine resonance and the inner silver center, resonating at 544.2 ppm.

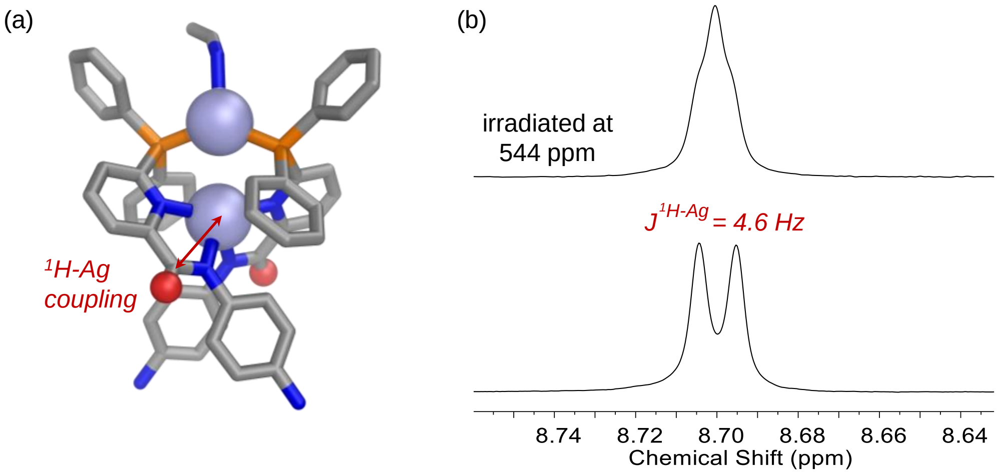

Figure S23: (a) Magnification of one of the disilver vertices of **2** with the imine protons highlighted as red spheres. The observed ^{1}H-Ag coupling is highlighted by a red arrow. (b) Coalescence of the imine signal in the ^{1}H NMR spectrum (500 MHz, 298 K, CD_3CN) of **2**·12OTf upon irradiation of ^{109}Ag at 544 ppm.

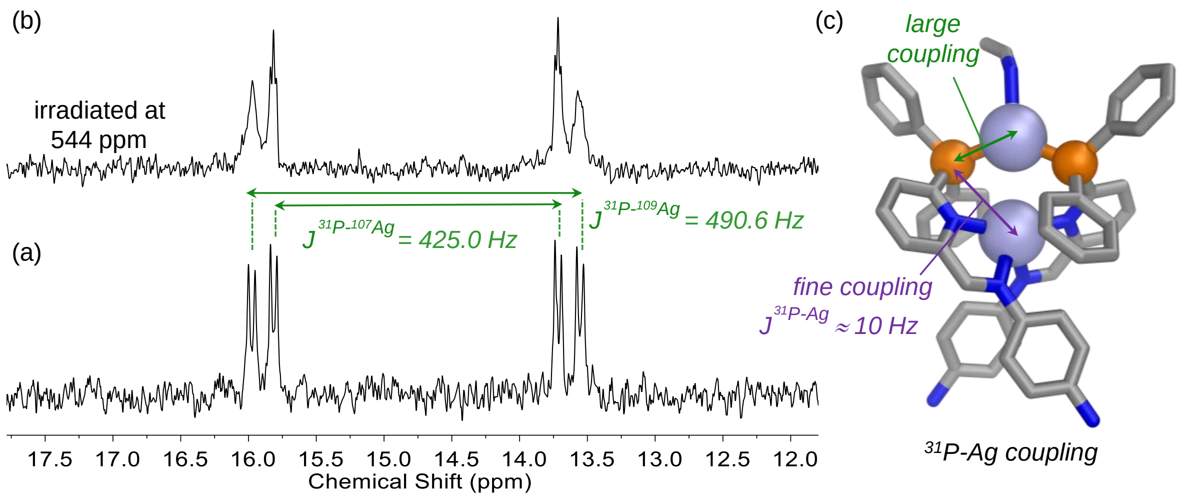

Figure S24: (a) ^{31}P NMR spectrum (162 MHz, CD_3CN) of **2**·12OTf. The signals arising from coupling to the directly bonded outer silver center (as both ^{107}Ag and ^{109}Ag) are labelled. Each peak is further split by fine coupling (≈10Hz) to the more distant inner silver center. (b) Irradiation of ^{109}Ag at 544 ppm partially eliminates the fine coupling to ^{109}Ag. (c) Magnification of one of the disilver vertices of **2** with the phosphines highlighted as orange spheres. The observed ^{31}P-Ag couplings are highlighted by green and purple arrows for the large coupling to the outer silver center and the fine coupling to the inner silver center respectively.

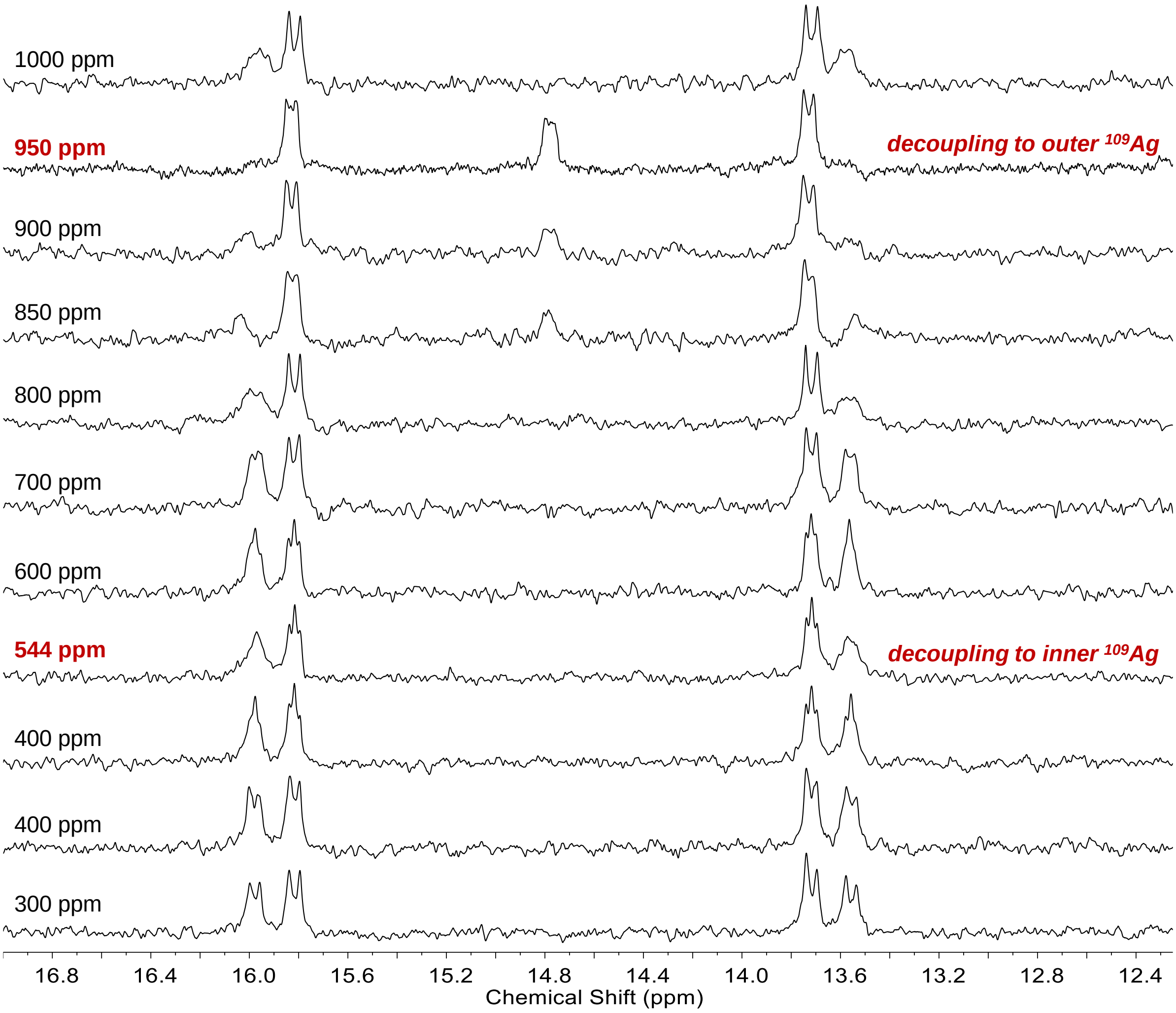

Figure S25: ^{31}P NMR spectra (162 MHz, CD_3CN) of **2**·12OTf, each with irradiation of ^{109}Ag at a different chemical shift. Collapse of the larger coupling with irradiation of ^{109}Ag at 544 nm and of the fine coupling with irradiation at 950 ppm are highlighted.

1.4. Reaction of cages 1 and 2 to form a library of mixed-metal cages

A mixture of **1**·12OTf (2.50 mg, 0.33 μmol) and **2**·12OTf (2.67 mg, 0.33 μmol) was dissolved in CD_3CN (0.5 mL) and equilibrated at room temperature for 3 days. The reaction mixture was analysed by ESI-MS (Figure S26) which was consistent with the formation of a library of $[Cu_xAg_{(12-x)}L_4](OTf)_{12}$ cages. A similar library of cages was obtained when tris(4-aminophenyl)amine (4 equiv.) and 2-formyl-6-diphenylphosphinopyridine (12 equiv.) were treated with equimolar amounts of copper(I) and silver(I) triflate (6 equiv. each).

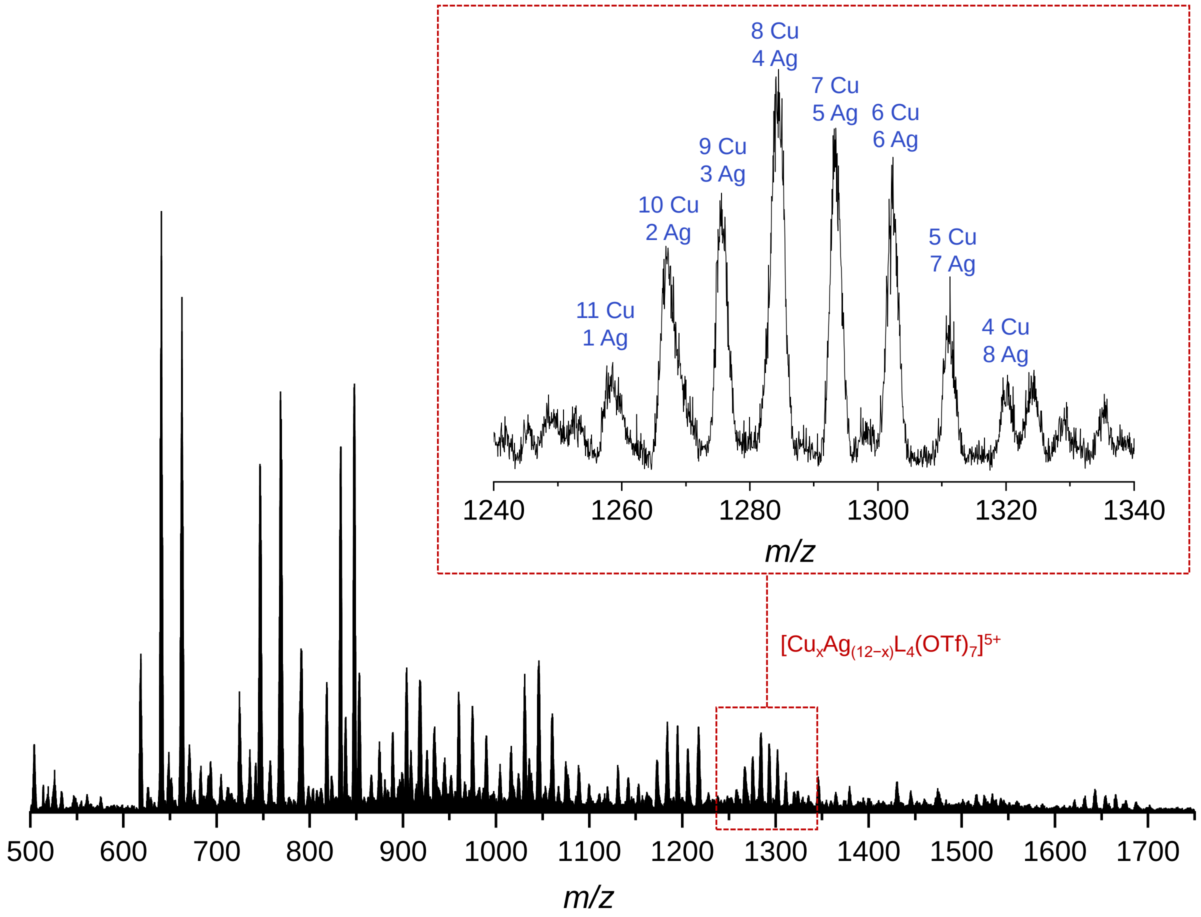

Figure S26: Low resolution ESI-mass spectrum of a 1:1 mixture of **1**·12OTf and **2**·12OTf showing an expansion of the +5 peaks.

1.5. Synthesis and characterization of cage 3

Cage **3** was prepared as both the perchlorate and triflate salts.

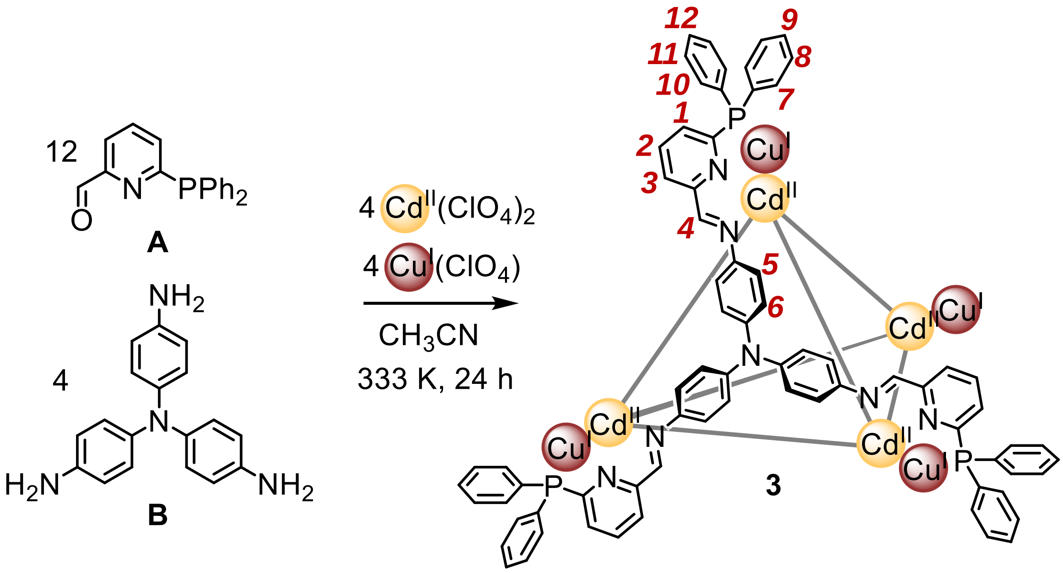

Scheme S3: Subcomponent self-assembly of **3**·$12ClO_4$.

Perchlorate salt:

Tris(4-aminophenyl)amine **B** (3.87 mg, 13.3 µmol, 4 equiv.), tetrakis(acetonitrile)copper(I) perchlorate (4.36 mg, 13.3 µmol, 4 equiv.), cadmium(II) perchlorate hexahydrate (5.59 mg, 13.3 µmol, 4 equiv.) and 2-formyl-6-diphenylphosphinopyridine **A** (11.65 mg, 40.2 µmol, 12 equiv.) were combined in CH_3CN (3 mL). The solution was degassed by three evacuation/nitrogen fill cycles and then heated at 333 K for 12 h in a sealed vessel under a nitrogen atmosphere, yielding a red-brown solution. The reaction mixture was then filtered through a plug of glass fibre filter paper and concentrated under a stream of N_2 to a volume of 1 mL. Addition of diethyl ether (4 mL) resulted in the precipitation of a dark red powder. The suspension was then centrifuged (10 min, 3000 RPM) and the eluent decanted. Further diethyl ether (4 mL) was added and the powder was then resuspended by sonication, centrifuged again and the eluent decanted. The residue was then dried *in vacuo* to afford the solid product as a fine dark red powder in approximately quantitative yield.

^{1}H NMR (500 MHz; 298 K; CD_3CN): δ 8.38 (s + d, $J^{1H\text{-}113Cd}$ = 31.5 Hz, 12H, H_4), 8.14 (t, J = 7.8 Hz, 12H, H_2), 7.95 (d, J = 7.8 Hz, 12H, H_3), 7.70 (t, J = 7.5 Hz, 12H, H_9), 7.62 (t, J = 7.7 Hz, 24H, H_8), 7.40-7.28 (m, 60H, H_1, H_7 & H_{12}), 7.03 (t, J = 7.6 Hz, 24H, H_{11}), 7.69 (m, 24H, H_{10}), 6.69 (d, J = 8.8 Hz, 24H, H_6), 5.63 (d, J = 8.8 Hz, 24H, H_5). Signals for the bound acetonitrile ligands (which were observed in the solid state) could not be identified in the ^{1}H NMR spectrum due to rapid exchange with CD_3CN.

^{13}C NMR (126 MHz, CD_3CN): δ 168.7 (imine), 158.5 (m), 150.5 (m), 149.9, 146.3, 145.0, 144.9, 137.4, 135.9, 134.0-133.0 (multiple overlapping signals), 131.8, 131.4, 127.5, 124.6. Some of the expected peaks are not detected due to a combination of low intensity, coupling to ^{31}P and signal overlap.

^{31}P NMR (162 MHz, CD_3CN): δ 9.68 (br s).

ESI-MS: *m/z* = 606.2 $[Cd_4Cu_4L_4(ClO_4)_3]^{9+}$, 957.0 $[Cd_4Cu_4L_4(ClO_4)_6]^{6+}$, 1168.1 $[Cd_4Cu_4L_4(ClO_4)_7]^{5+}$, 1485.4 $[Cd_4Cu_4L_4(ClO_4)_8]^{4+}$.

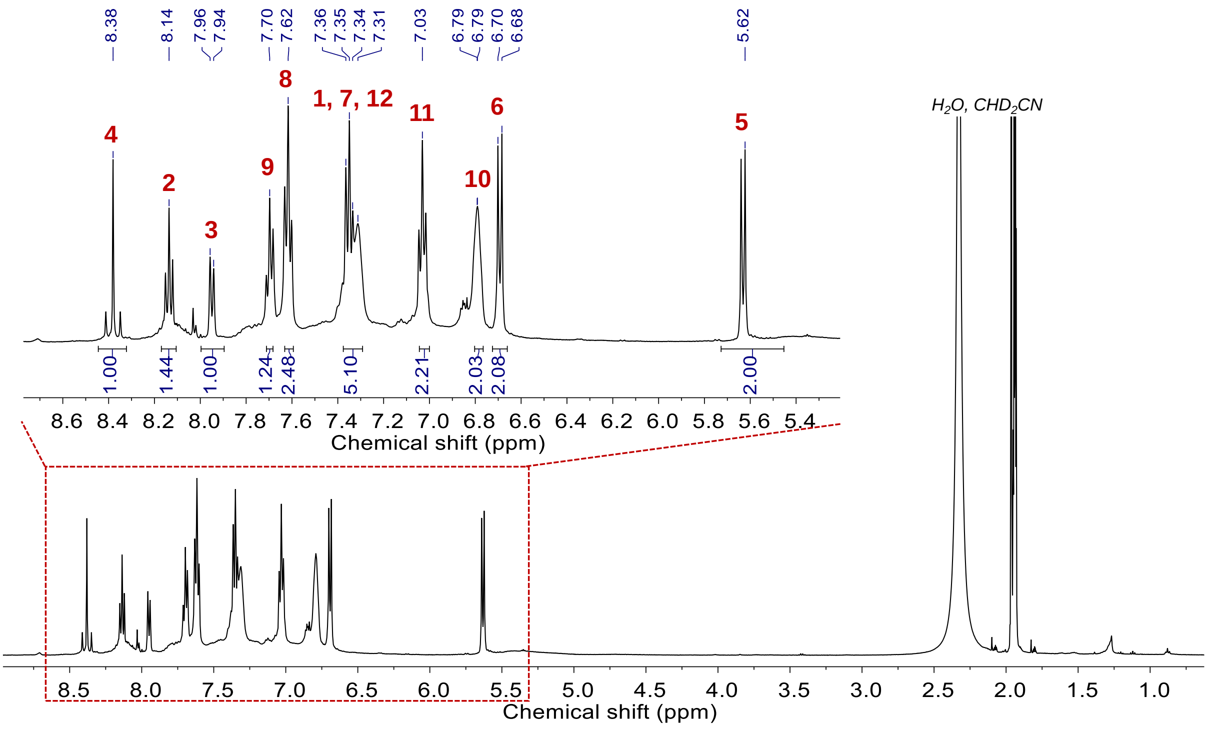

Figure S27: ^1H NMR spectrum (500 MHz, 298 K, CD_3CN) of **3**·$12ClO_4$ with inset showing the aromatic region. The numbering scheme for the proton assignments is shown in Scheme S3.

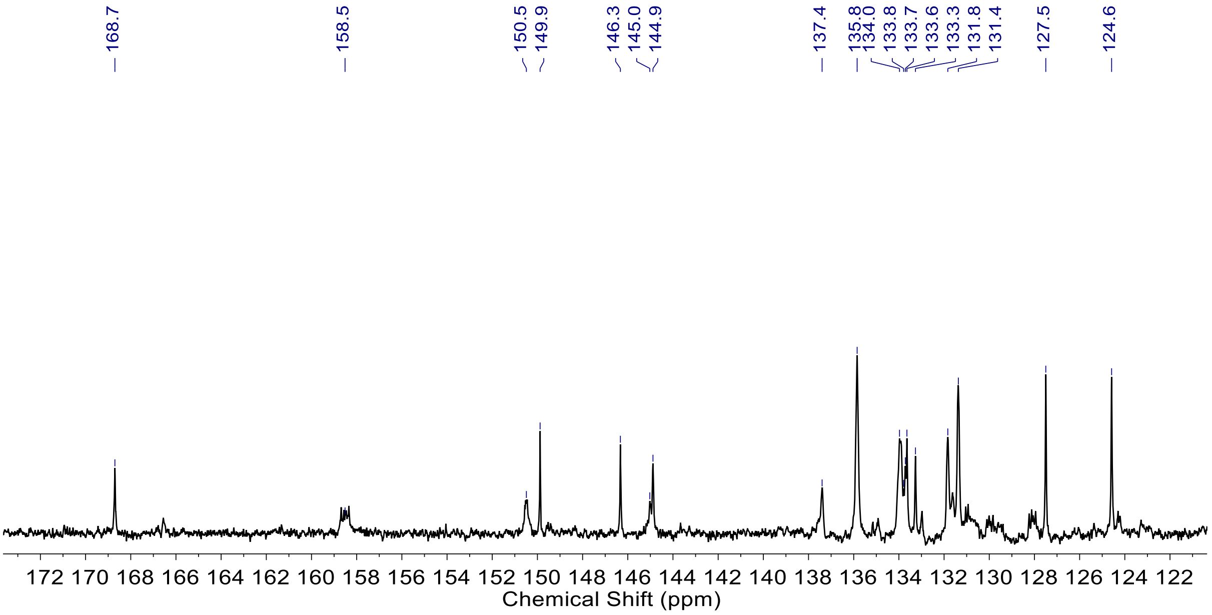

Figure S28: ^{13}C NMR spectrum (500 MHz, 298 K, CD_3CN) of **3**·$12ClO_4$.

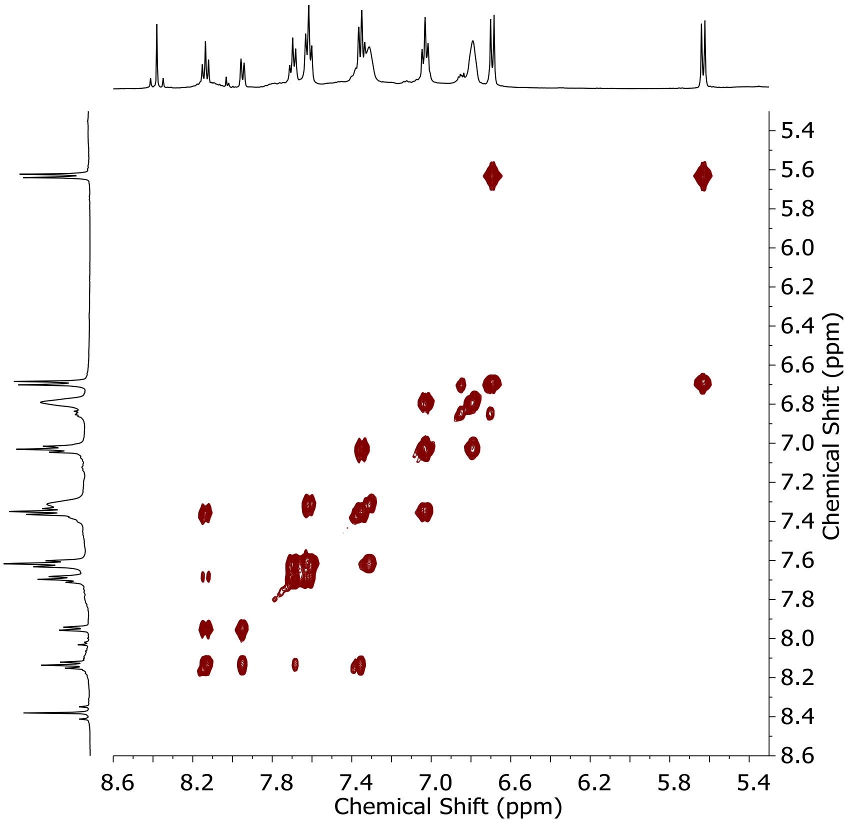

Figure S29: Aromatic region of the ^1H-^1H COSY spectrum (500 MHz, 298 K, CD_3CN) of **3**·$12ClO_4$.

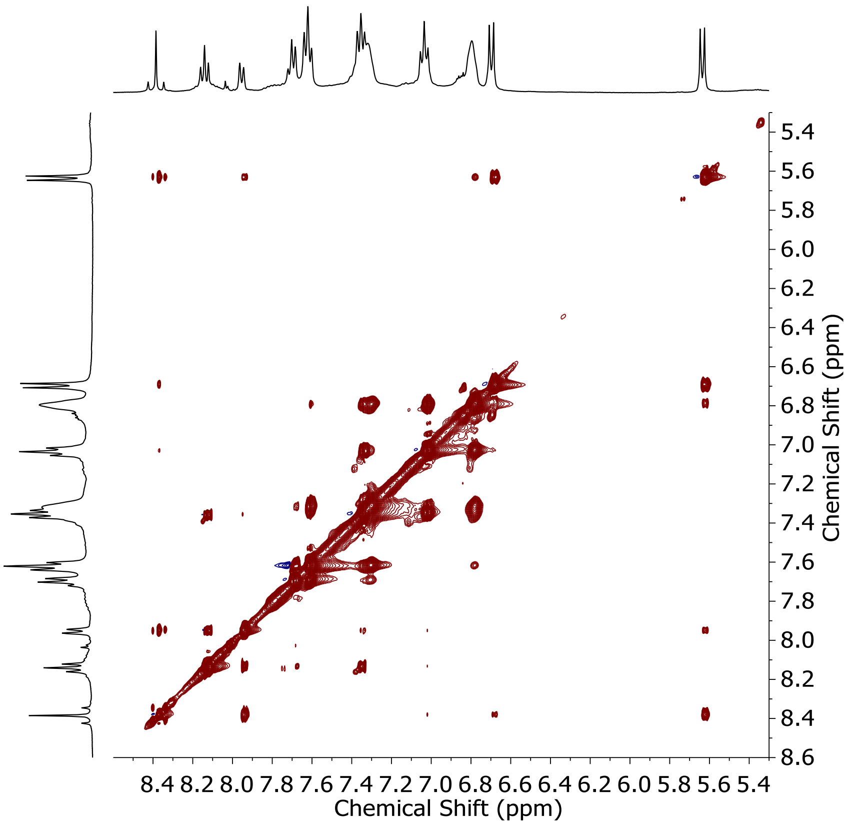

Figure S30: Aromatic region of the ^{1}H-^{1}H NOESY spectrum (500 MHz, 298 K, CD_3CN) of **3**·$12ClO_4$.

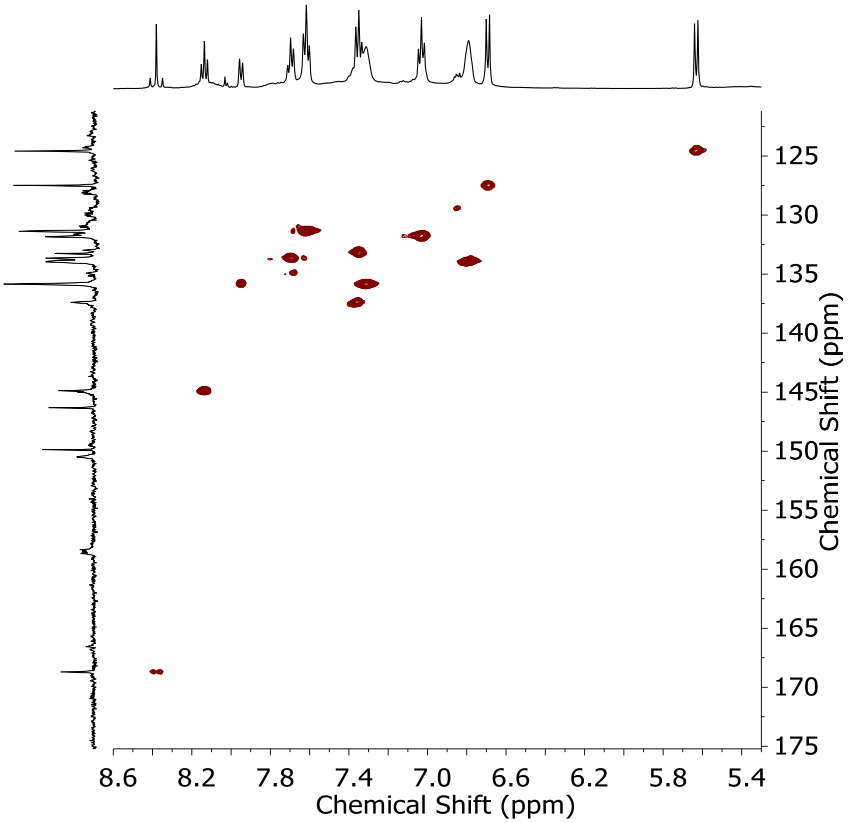

Figure S31: Aromatic region of the ^{1}H-^{13}C HSQC spectrum (500 MHz, 298 K, CD_3CN) of **3**·$12ClO_4$.

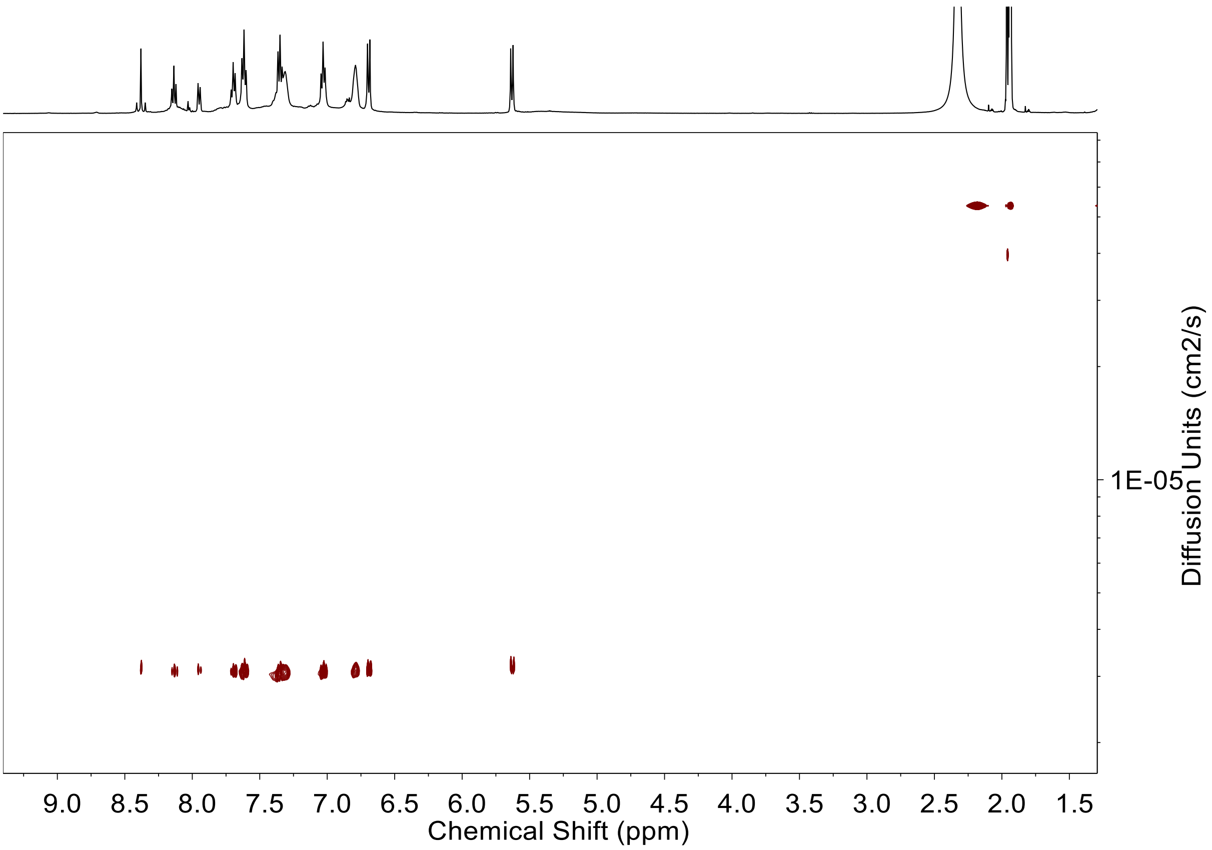

Figure S32: ^{1}H DOSY spectrum (400 MHz, 298 K, CD_3CN) of **3**·$12ClO_4$. The diffusion coefficient was measured to be (3.1 ± 0.1) × 10^{-6} cm^2 s^{-1}.

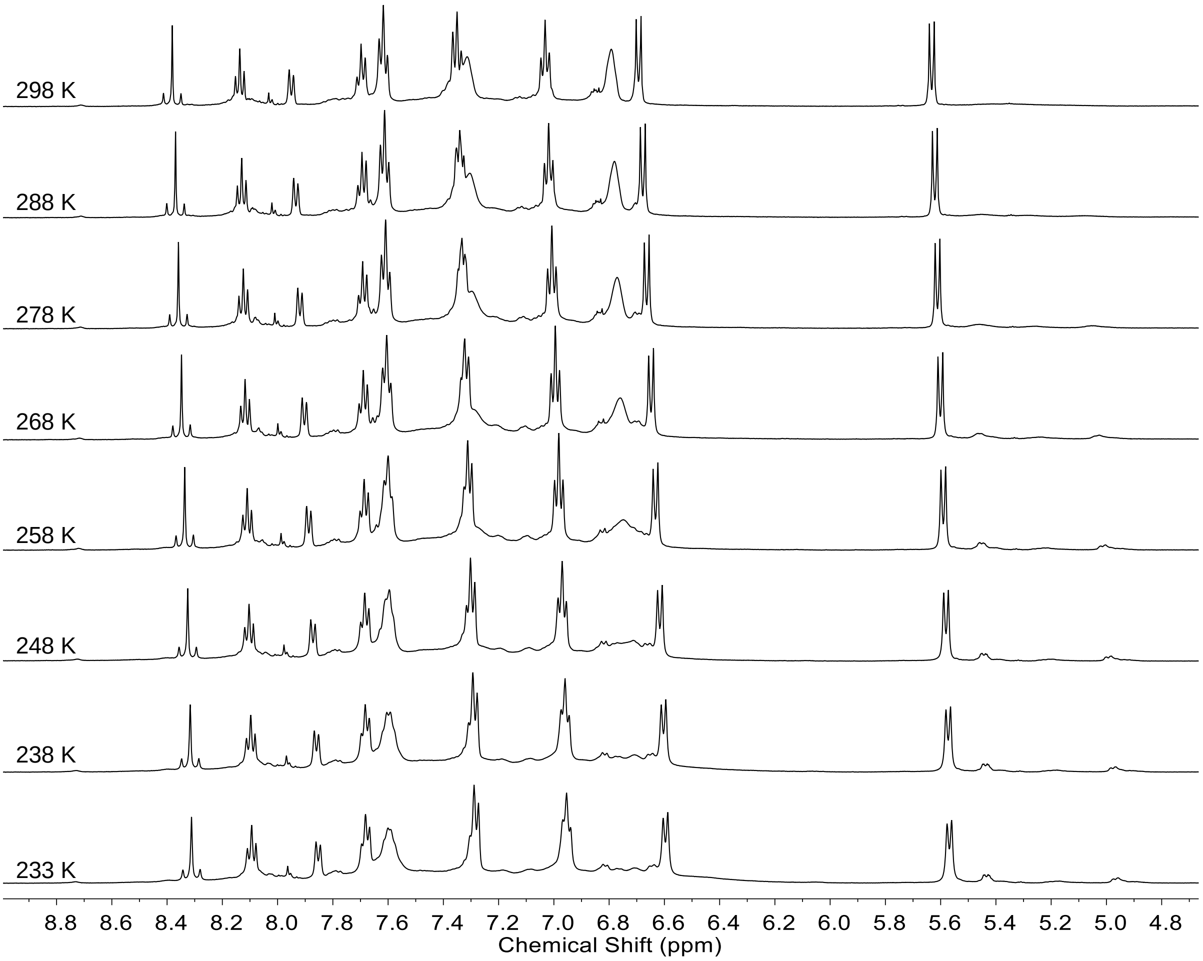

Figure S33: VT-NMR stack plot (500 MHz, CD_3CN) showing the effect of temperature on the ^1H NMR spectrum of cage **3**·$12ClO_4$.

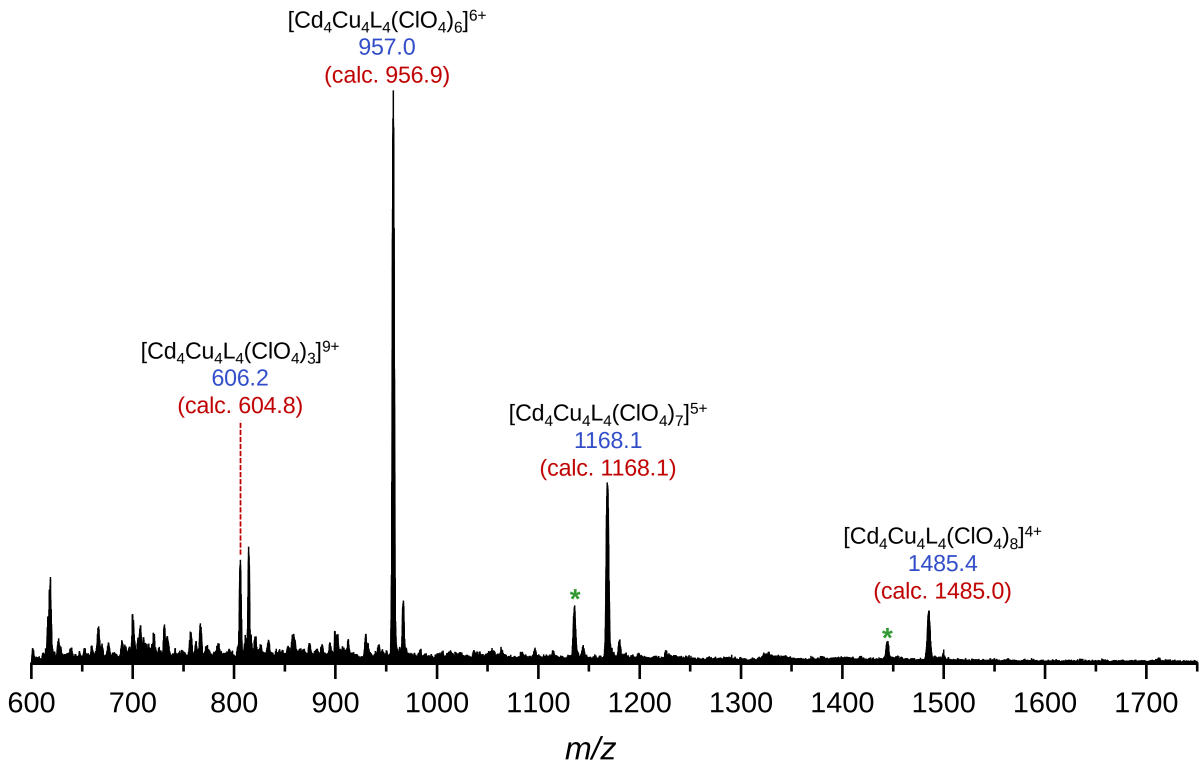

Figure S34: Low resolution ESI-mass spectrum of **3**·12ClO4. The green asterisks indicate signals corresponding to **3**·12ClO4 with the loss of one Cu[I] cation. Note that the weakly coordinated acetonitrile ligands were not observed under the MS conditions, presumably due to their loss during the ionization process.

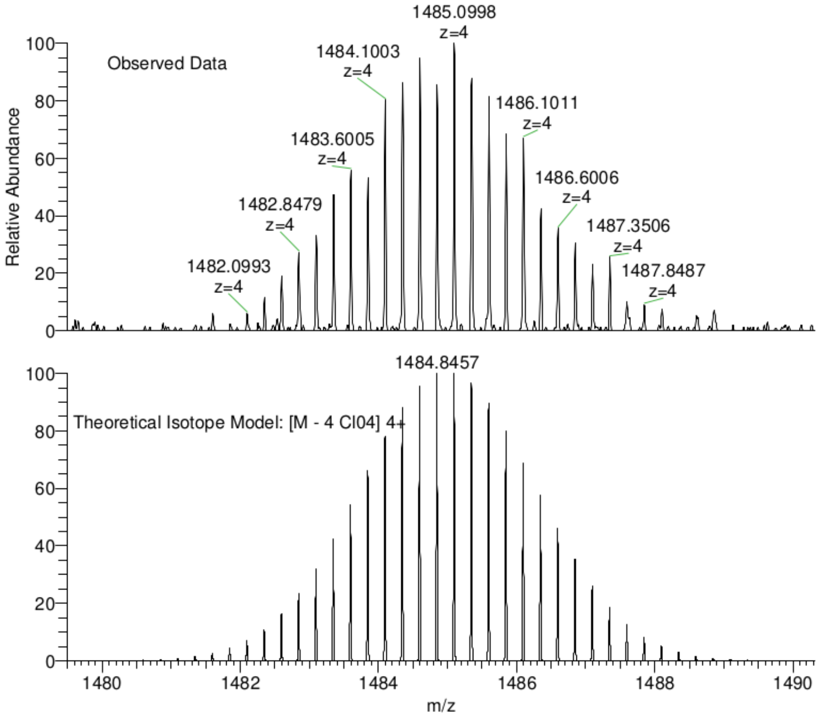

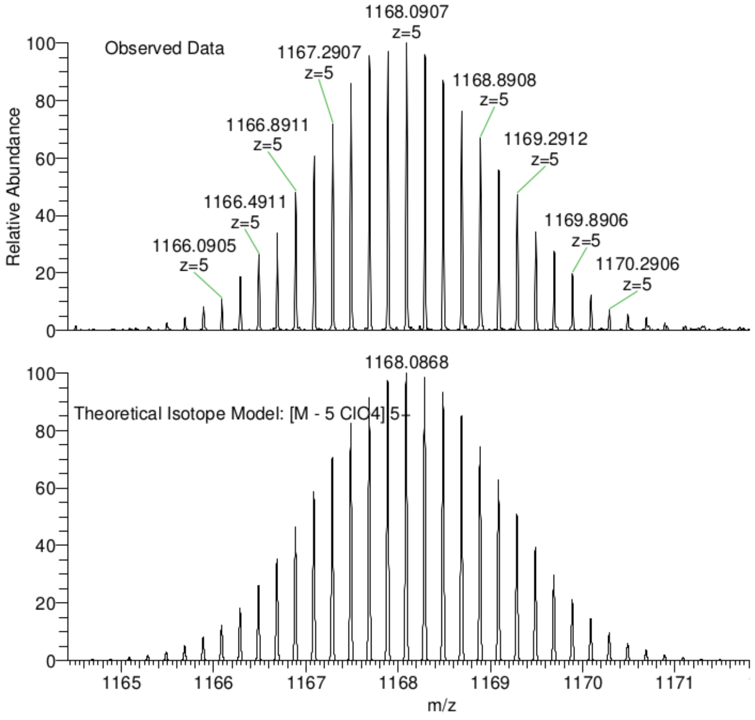

Figure S35: High-resolution ESI-mass spectrometry analysis of **3**·12ClO_4 showing the observed (top) and theoretical (bottom) isotope patterns the +4 and +5 peaks.

Triflate salt:

The triflate salt of **3** was prepared via an identical procedure to the perchlorate salt except that tetrakis(acetonitrile)copper(I) triflate and cadmium(II) triflate were used as the metal salts. All spectral data were very similar to **3**·12ClO$_4$. The triflate salt was used to prepare single crystals of **3** (see Section 2 below).

1.6. Synthesis and characterization of cage 4

Cage **4** was prepared as both the perchlorate and triflate salts.

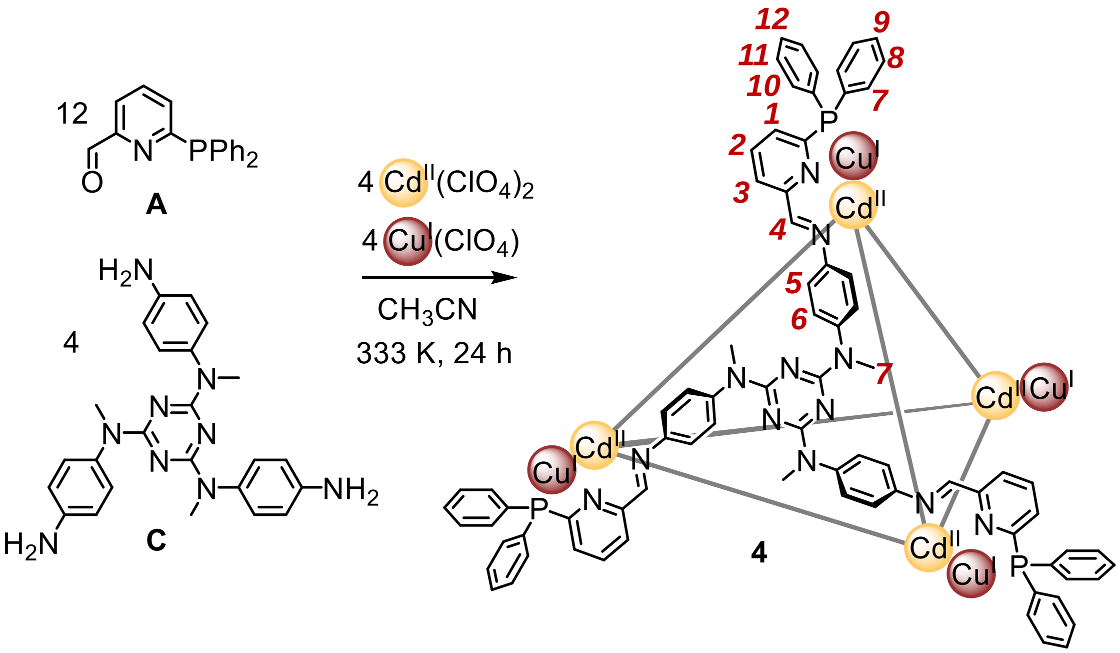

Scheme S4: Subcomponent self-assembly of **4**·12ClO$_4$.

Perchlorate salt:

N2,N4,N6-tris(4-aminophenyl)-N2,N4,N6-trimethyl-1,3,5-triazine-2,4,6-triamine **C** (5.89 mg, 13.4 µmol, 4 equiv.), tetrakis(acetonitrile)copper(I) perchlorate (4.36 mg, 13.3 µmol, 4 equiv.), cadmium(II) perchlorate hexahydrate (5.59 mg, 13.3 µmol, 4 equiv.) and 2-formyl-6-diphenylphosphinopyridine **A** (11.65 mg, 40.2 µmol, 12 equiv.) were combined in CH$_3$CN (3 mL). The solution was degassed by three evacuation/nitrogen fill cycles and then heated at 333 K for 12 h in a sealed vessel under a nitrogen atmosphere, yielding a red-brown solution. The reaction mixture was then filtered through a plug of glass fibre filter paper and concentrated under a stream of N$_2$ to a volume of 1 mL. Addition of diethyl ether (4 mL) resulted in the precipitation of a dark red powder. The suspension was then centrifuged (10 min, 3000 RPM) and the eluent decanted. Further diethyl ether (4 mL) was added and the powder

was then resuspended by sonication, centrifuged again and the eluent decanted. The residue was then dried *in vacuo* to afford the solid product as fine dark brown powder (23 mg, 97%).

^{1}H NMR (500 MHz; 298 K; CD_3CN): δ 8.25 (t, 7.8 Hz, 12H, H_2), 8.21 (s + d, $J^{1H\text{-}113Cd}$ = 33.1 Hz, 12H, H_4), 7.88 (d, J = 7.8 Hz, 12H, H_3), 7.73 (m, 12H, H_{10}), 7.65 (t, J = 7.6 Hz, 12H, H_9), 7.44 (d, J = 8.0 Hz, 12H, H_1), 7.42 – 7.28 (m, 60H, H_{13}, H_8 & H_6), 7.10 (t, J = 7.6 Hz, 24H, H_{12}), 6.85 (br s, 24H, H_{11}), 5.03 (br s, 24H, H_5), 3.39 (s, 36H, H_7). Signals for the bound acetonitrile ligands (which were observed in the solid state) could not be identified in the ^{1}H NMR spectrum due to rapid exchange with CD_3CN.

^{13}C NMR (126 MHz, CD_3CN): δ 166.3, 165.4, 157.9 (m), 149.9 (m), 144.7, 144.6, 137.1, 135.5, 134.6, 133.7, 133.3, 132.7, 131.3, 131.0, 129.2 (m), 127.5 (m), 122.0, 38.0. Some of the expected peaks are not detected due to a combination of low intensity, coupling to ^{31}P and signal overlap.

^{31}P NMR (162 MHz, CD_3CN): δ 10.27 (br s).

ESI-MS: m/z = 672.3 $[Cd_4Cu_4L_4(ClO_4)_3]^{9+}$, 768.5 $[Cd_4Cu_4L_4(ClO_4)_4]^{8+}$, 892.5 $[Cd_4Cu_4L_4(ClO_4)_5]^{7+}$, 1057.9 $[Cd_4Cu_4L_4(ClO_4)_6]^{6+}$, 1289.2 $[Cd_4Cu_4L_4(ClO_4)_7]^{5+}$, 1636.6 $[Cd_4Cu_4L_4(ClO_4)_8]^{4+}$.

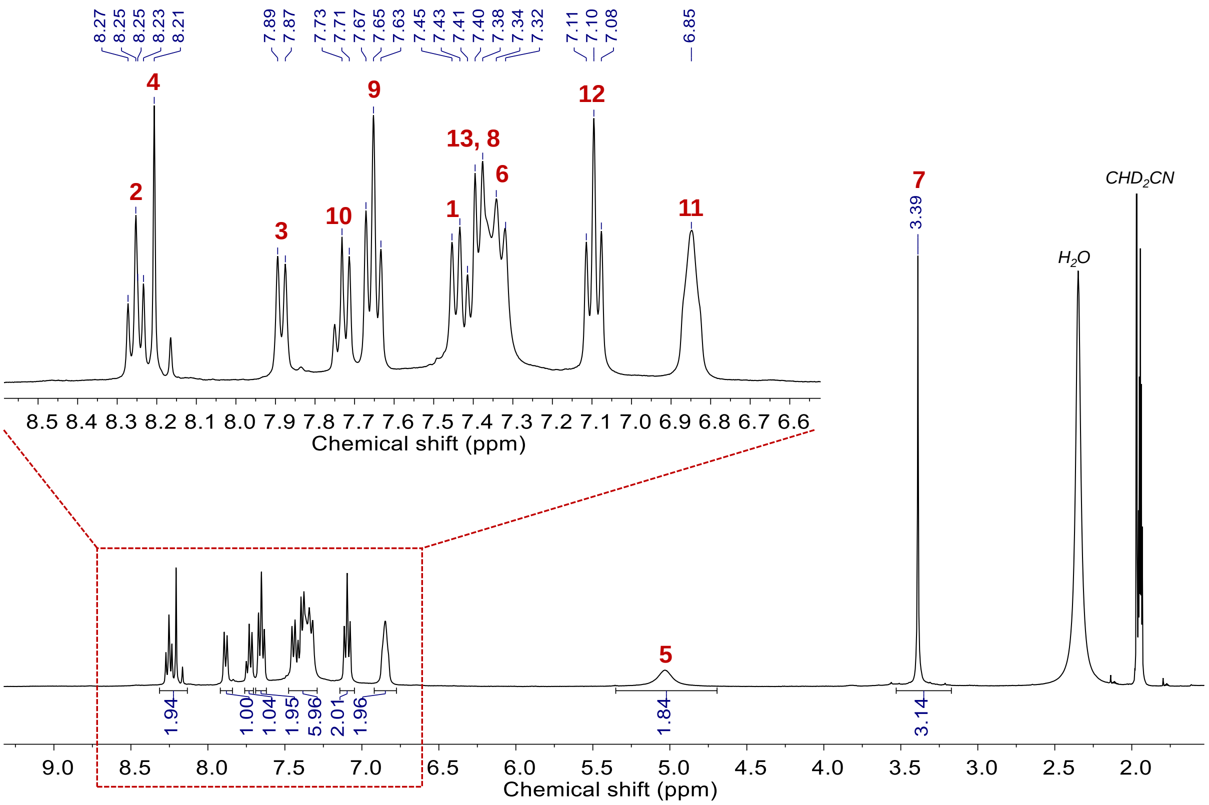

Figure S36: ^{1}H NMR spectrum (500 MHz, 298 K, CD_3CN) of **4**·12ClO_4 with inset showing the aromatic region. The numbering scheme for the proton assignments is shown in Scheme S4.

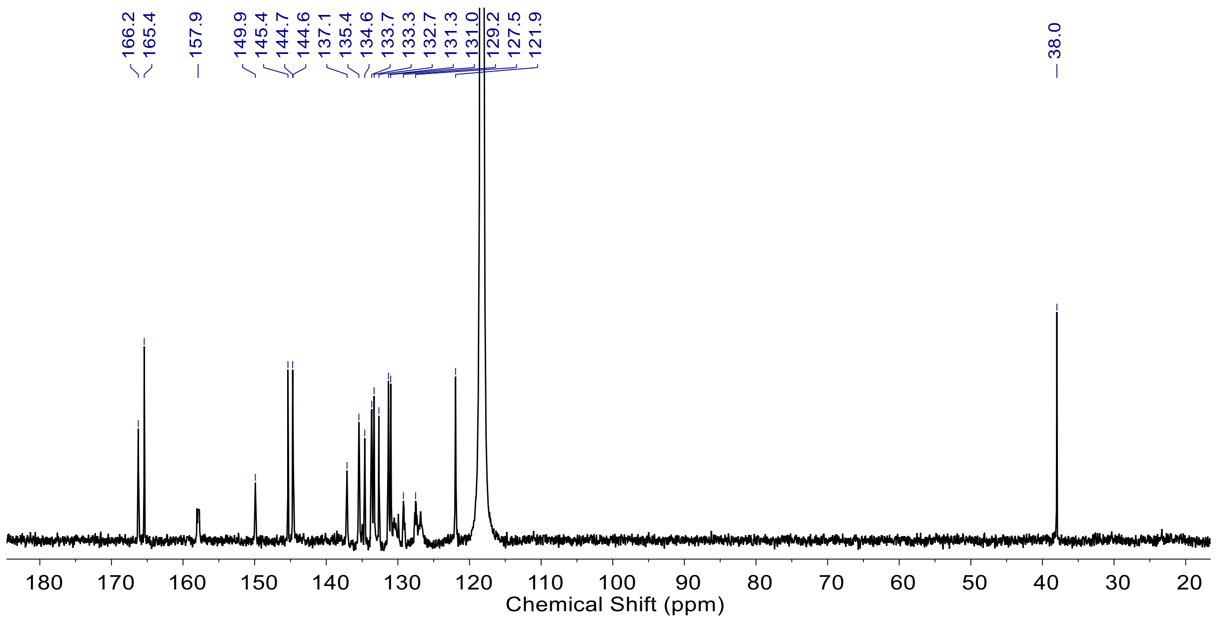

Figure S37: ^{13}C NMR spectrum (126 MHz, 298 K, CD_3CN) of **4**·$12ClO_4$.

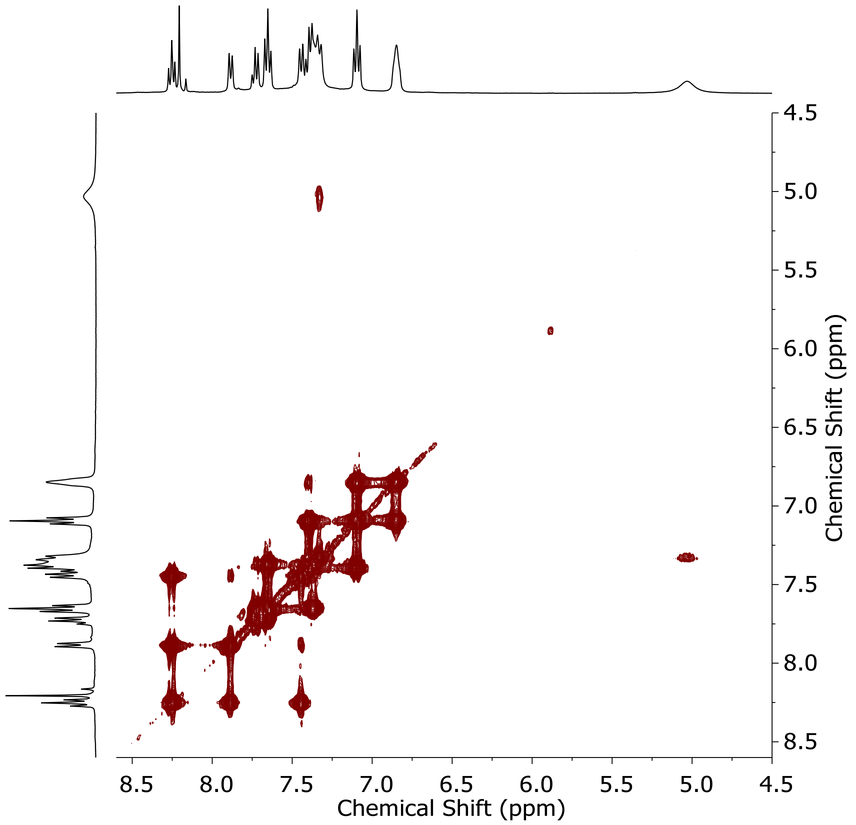

Figure S38: Aromatic region of the ^{1}H-^{1}H COSY spectrum (500 MHz, 298 K, CD_3CN) of **4**·$12ClO_4$.

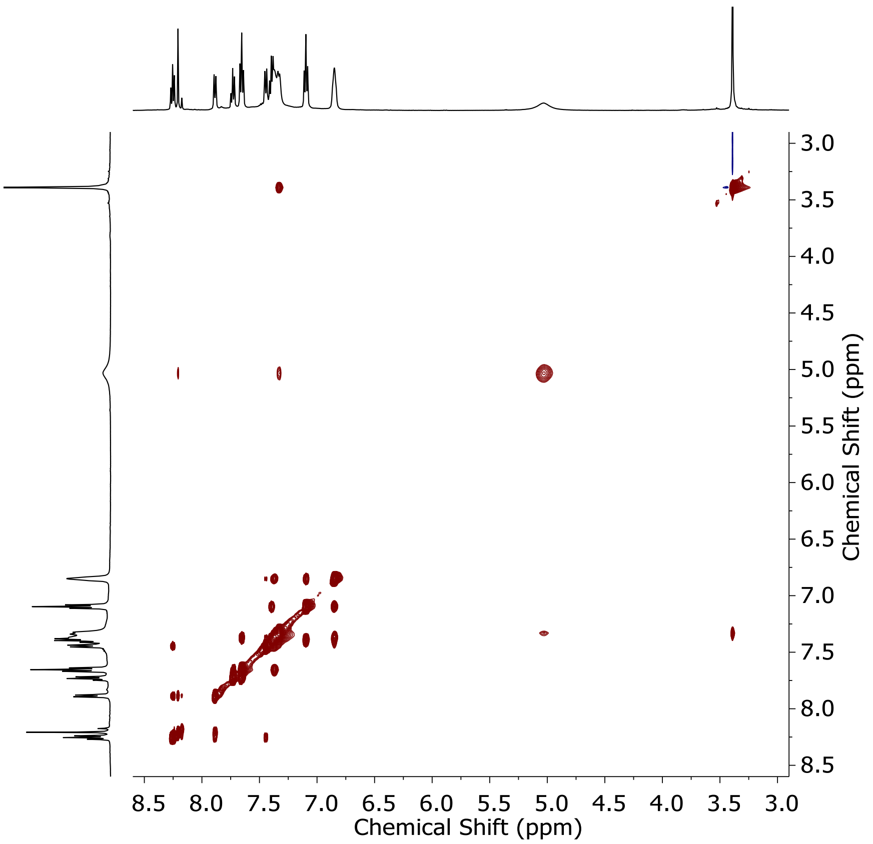

Figure S39: Aromatic region of the ^{1}H-^{1}H NOESY spectrum (500 MHz, 298 K, CD_3CN) of **4**·$12ClO_4$.

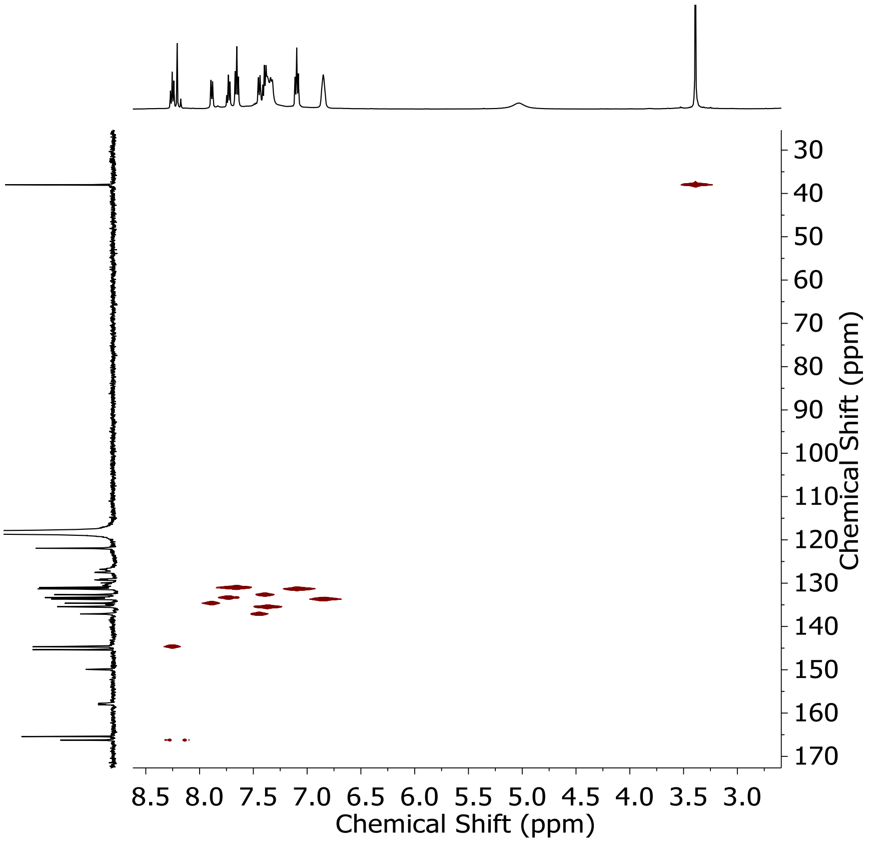

Figure S40: Aromatic region of the ^{1}H-^{13}C HSQC spectrum (500 MHz, 298 K, CD_3CN) of **4**·$12ClO_4$.

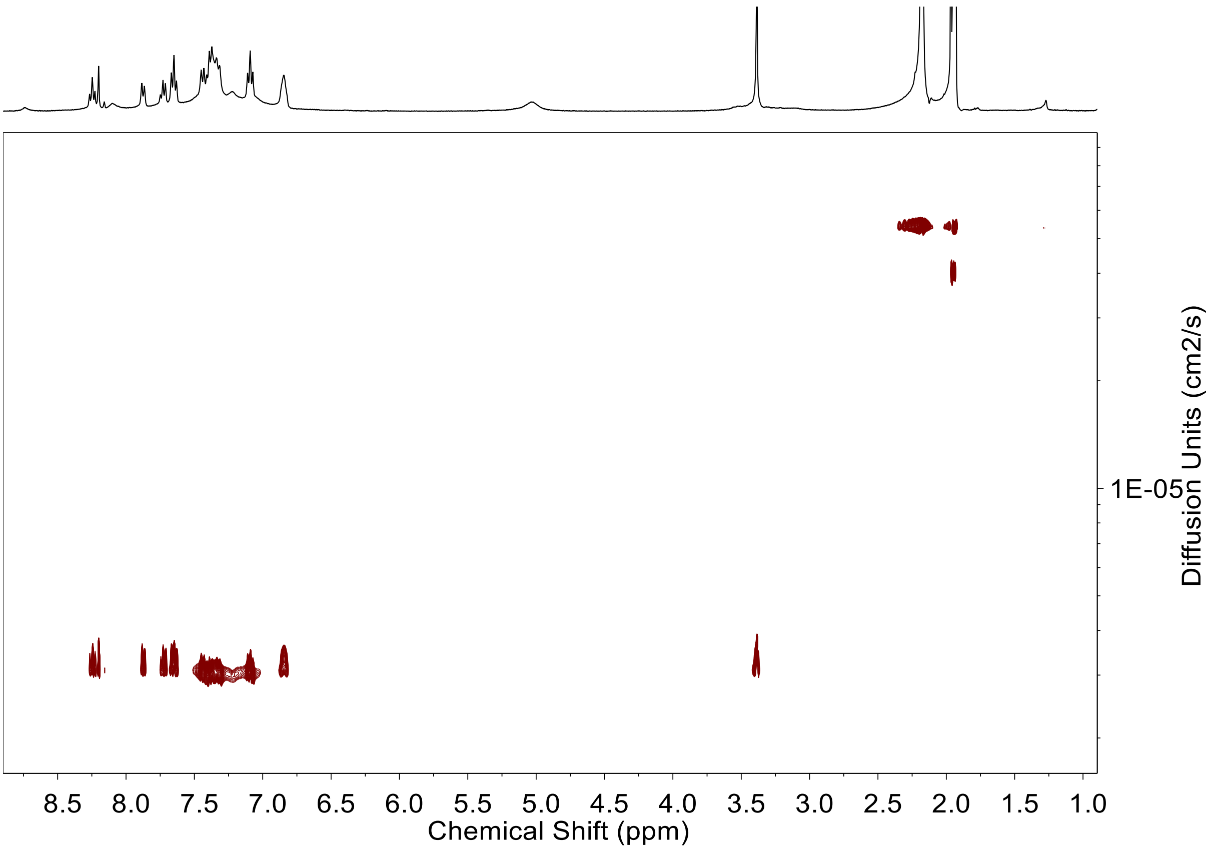

Figure S41: ^{1}H DOSY spectrum (400 MHz, 298 K, CD_3CN) of **4**·$12ClO_4$. The diffusion coefficient was measured to be $(3.3 \pm 0.1) \times 10^{-6}$ cm^2 s^{-1}.

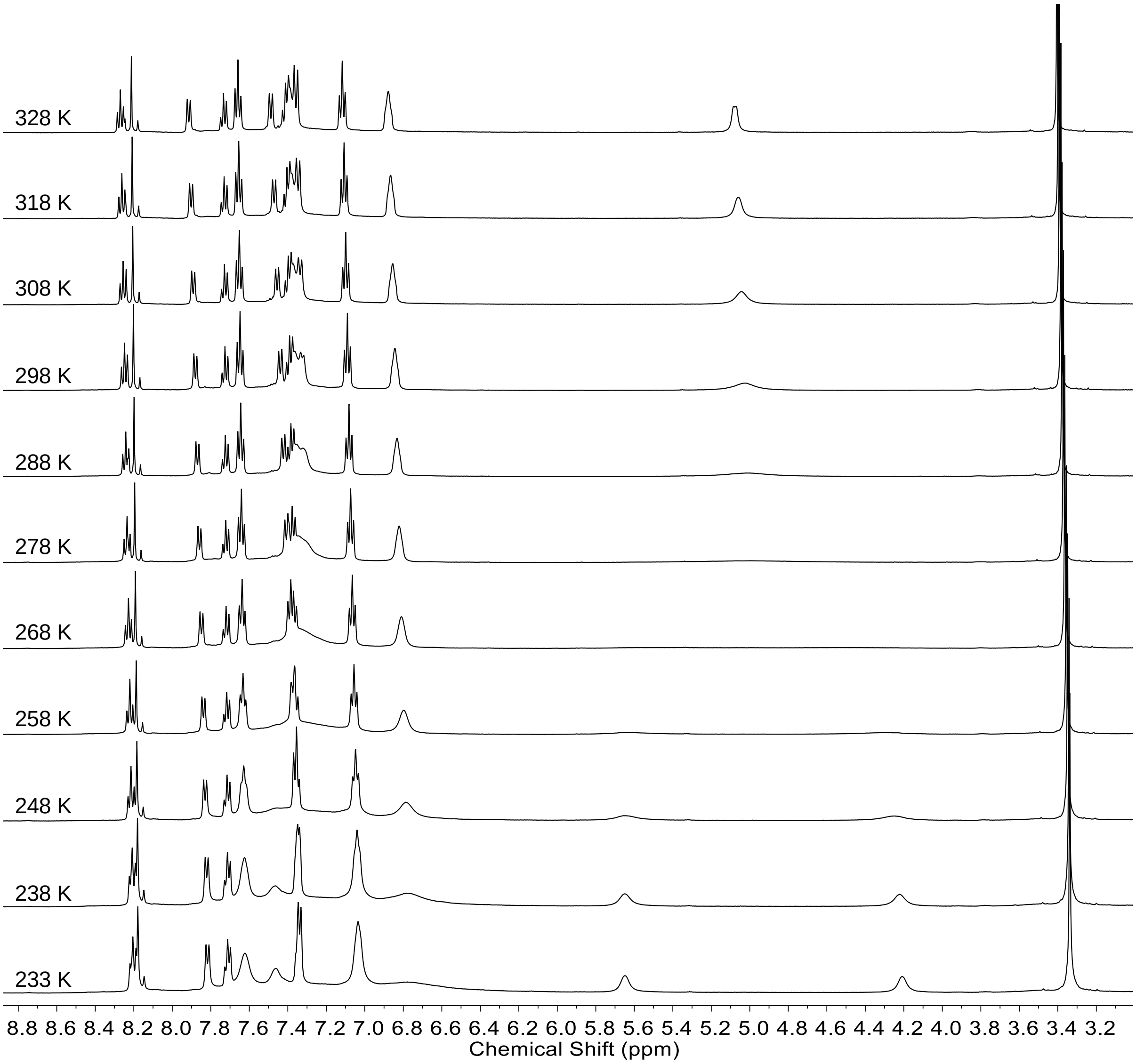

Figure S42: VT-NMR stack plot (500 MHz, CD_3CN) showing the effect of temperature on cage **4**·$12ClO_4$.

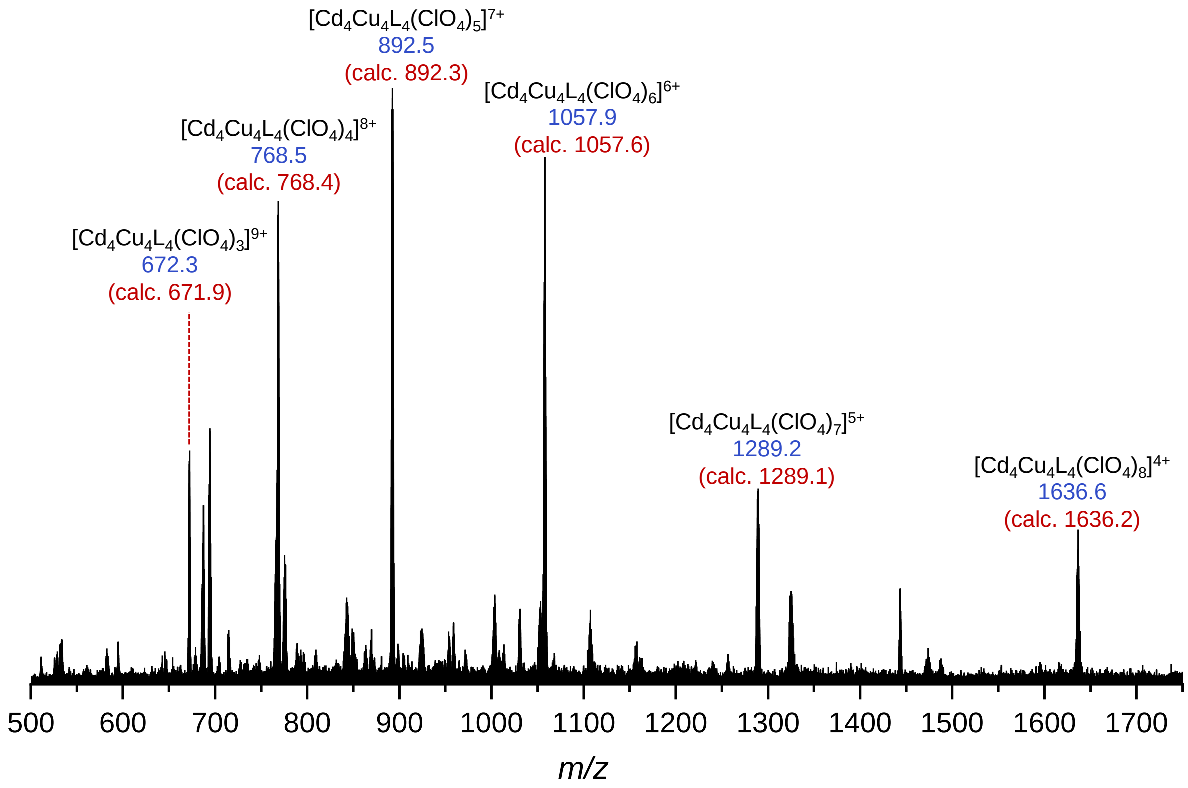

Figure S43: Low resolution ESI-mass spectrum of **4**·12ClO$_4$. Note that the weakly coordinated acetonitrile ligands were not observed under the MS conditions, presumably due to their loss during the ionization process.

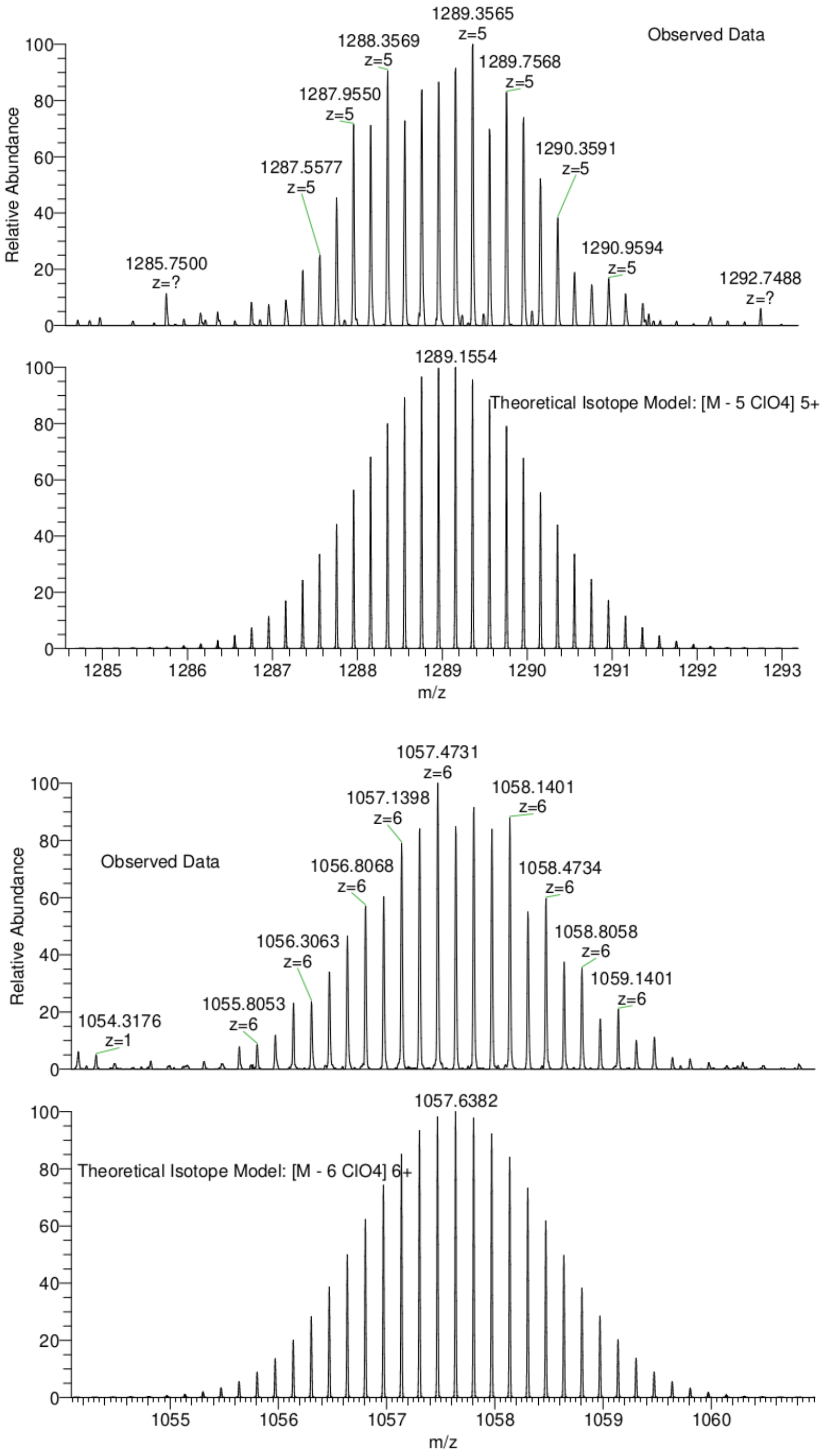

Figure S44: High-resolution ESI-mass spectrometry analysis of **4**·12ClO_4 showing the observed (top) and theoretical (bottom) isotope patterns the +5 and +6 peaks.

Triflate salt:

The triflate salt of **4** was prepared via an identical procedure to the perchlorate salt except that tetrakis(acetonitrile)copper(I) triflate and cadmium(II) triflate were used as the metal salts. All spectral data were very similar to **4**·$12ClO_4$. The triflate salt was used to prepare single crystals of **3** (see Section 2 below).

2. X-ray crystallography

Data for all structures except for the perchlorate salt of **1** were collected at Beamline I19 of Diamond Light Source employing silicon double crystal monochromated synchrotron radiation (0.6889 Å) with ω and ψ scans at 100(2) K.[3] Data integration and reduction were undertaken with Xia2[4] and multi-scan empirical absorption corrections were applied to the data using the AIMLESS[5] tool in the CCP4 suite.[6] Data for the perchlorate salt of **1** were collected using a Bruker D8 VENTURE equipped with high-brilliance IµS Cu-Kα radiation (1.54178 Å), with ω and ψ scans at 180(2) K. Data integration and reduction were undertaken with SAINT and XPREP[7] and multi-scan empirical absorption corrections were applied to the data using SADABS.[7] In all cases subsequent computations were carried out using the WinGX-32 graphical user interface.[8] The structures were solved by dual space methods using SHELXT[9] or charge-flipping using SUPERFLIP[10] then refined and extended with SHELXL.[11] In general, non-hydrogen atoms with occupancies greater than 0.5 were refined anisotropically. Carbon-bound hydrogen atoms were included in idealised positions and refined using a riding model. Disorder was modelled using standard crystallographic methods including constraints, restraints and rigid bodies where necessary. Crystallographic data along with specific details pertaining to the refinement follow. Crystallographic data have been deposited with the CCDC (2153619-2153622 and 2163435).

Two data sets were collected for cage **1**, from two different samples, grown in the presence of different counterions. The crystallographic data for both data sets are presented here but only the highest resolution data set, obtained for crystals with composition $[Cu_{12}L_4(MeCN)_{12}]\cdot 11.7OTf\cdot 0.3ReO_4\cdot 2MeCN\cdot 1.5^iPr_2O$, is discussed in detail in the main text. Both data sets are isomorphous with respect to the $Cu_{12}L_4(MeCN)_{12}$ cations (except for minor differences in the disorder of the coordinated acetonitrile molecules).

Cage 1 (triflate salt) - $[Cu_{12}L_4(MeCN)_{12}]\cdot 11.7OTf\cdot 0.3ReO_4\cdot 2MeCN\cdot 1.5^iPr_2O$ [+ solvent]

Formula $C_{336.70}H_{279}Cu_{12}F_{35.10}N_{42}O_{37.80}P_{12}Re_{0.30}S_{11.70}$, *M* 7750.18, Cubic, space group P 2_1 3 (#198), *a* 43.215(5), *b* 43.215(5), *c* 43.215(5) Å, *V* 80705(28) Å^3, D_c 1.276 g cm^{-3}, *Z* 8, crystal size 0.040 by 0.030 by 0.030 mm, colour brown, habit prism, temperature 100(2) Kelvin, λ(Synchrotron) 0.6889 Å, μ(Synchrotron) 0.827 mm^{-1}, T(Analytical)$_{min,max}$ 0.924445967991, 1.0, $2\theta_{max}$ 47.80, *hkl* range -50 50, -50 50, -50 50, *N* 983792, N_{ind} 45877(R_{merge} 0.0551), N_{obs} 43473(I > 2σ(I)), N_{var} 2725, residuals* $R1(F)$ 0.0401, $wR2(F^2)$ 0.1028, GoF(all) 1.016, $\Delta\rho_{min,max}$ -0.244, 0.634 e$^-$ Å^{-3}.

$^*R1 = \Sigma||F_o| - |F_c||/\Sigma|F_o|$ for $F_o > 2\sigma(F_o)$; $wR2 = (\Sigma w(F_o^2 - F_c^2)^2/\Sigma(wF_c^2)^2)^{1/2}$ all reflections

$w=1/[\sigma^2(F_o^2)+(0.0827P)^2]$ where $P=(F_o^2+2F_c^2)/3$

Specific refinement details:

The crystals with composition $[Cu_{12}L_4(MeCN)_{12}]\cdot 11.7OTf\cdot 0.3ReO_4\cdot 2MeCN\cdot 1.5{}^iPr_2O$ [+ solvent] were grown by diffusion of diisopropyl ether into an acetonitrile solution of **1**·12OTf containing Bu_4NReO_4. The crystals employed immediately lost solvent after removal from the mother liquor and rapid handling prior to flash cooling in the cryostream was required to collect data. Data were obtained to 0.85 Å resolution. The asymmetric unit was found to contain one third each of two separate $Cu_{12}L_4$ assemblies and associated counterions and solvent molecules. The structure was refined as both a merohedral twin (twin law 0 1 0 1 0 0 0 0 -1) and a racemic twin with the BASF parameters refining to 0.21493/0.20971/0.29022.

Bond lengths and angles within pairs of chemically identical organic ligand arms were restrained to be similar to each other (SAME) and some aromatic rings were refined with rigid body constraints (AFIX 66). Thermal parameter restraints (SIMU, RIGU) were applied to all atoms except for copper and rhenium to facilitate anisotropic stable refinement. Two of the coordinated acetonitrile molecules were modelled as disordered over two locations and a further one shows evidence of thermal motion or minor unresolved disorder. These acetonitrile molecules were modelled with isotropic thermal parameters and bond length restraints (DFIX) were applied to all coordinated acetonitrile molecules.

The anions within the structure also show evidence of disorder. One triflate anion was modelled as disordered over two locations. One lattice site (on a special position) was modelled as a disordered mixture of triflate and perrhenate. A further triflate anion was modelled as disordered over a special position using a rigid group and another triflate was modelled with partial occupancy. The occupancies of the disordered anions were allowed to refine freely and in some cases were then fixed at the obtained values. Some disordered anions could not be located in the electron density map and were not included in the model resulting in a discrepancy of 4.28 anions per $Cu_{12}L_4$ assembly; these anions are included as triflate in the formula given above. Some lower occupancy disordered atoms were modelled with isotropic thermal parameters and bond length and thermal parameter restraints were applied to facilitate realistic modelling of the disordered anions.

Further reflecting the solvent loss there is a significant amount of void volume in the lattice containing smeared electron density from disordered solvent the unresolved anions. Consequently the SQUEEZE[12] function of PLATON[13] was employed to remove the contribution of the electron density associated with these highly disordered solvents and anions which gave a potential solvent accessible void of 19970 $Å^3$

per unit cell (a total of approximately 5351 electrons). Since the diffuse solvent molecules could not be assigned conclusively to acetonitrile or diisopropyl ether they were not included in the formula. Consequently, the molecular weight and density given above are likely to be slightly underestimated.

CheckCIF gives one B level alert, resulting from isotropic refinement of some of the coordinated acetonitrile molecules.

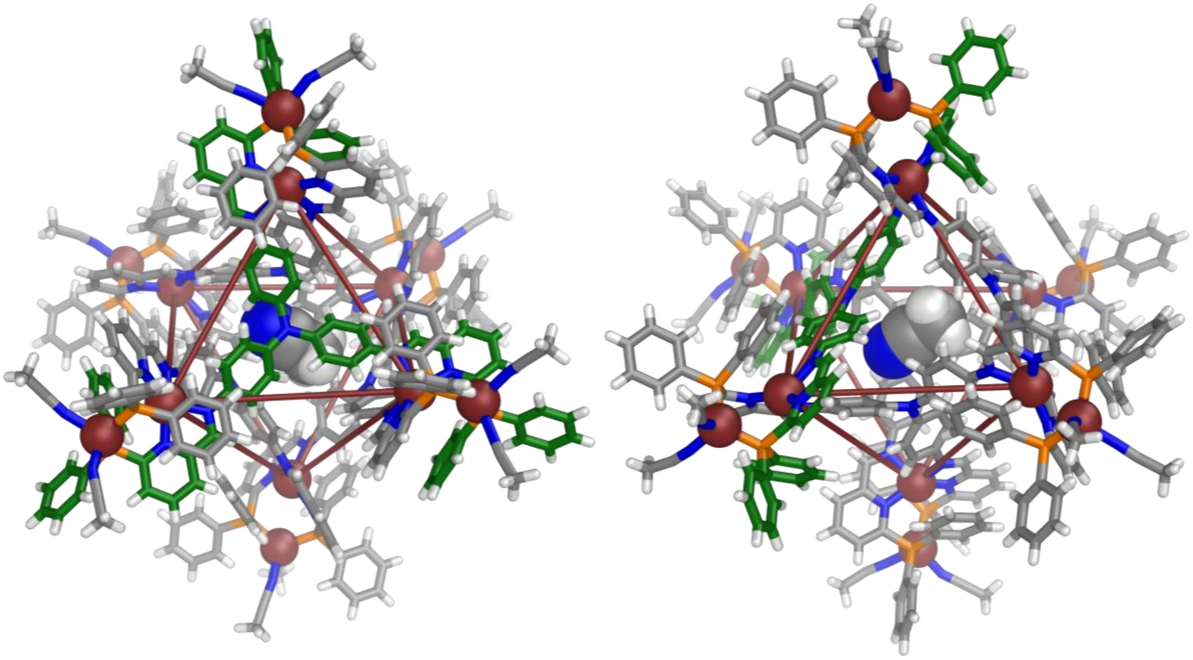

Figure S45: Two views of the cationic part of the crystal structure of **1**, looking down a ligand-occupied face (left) and an empty face (right). In both cases the carbon atoms of one of the ligands are highlighted in green and the acetonitrile bound within the cage cavity in shown in space-filling representation.

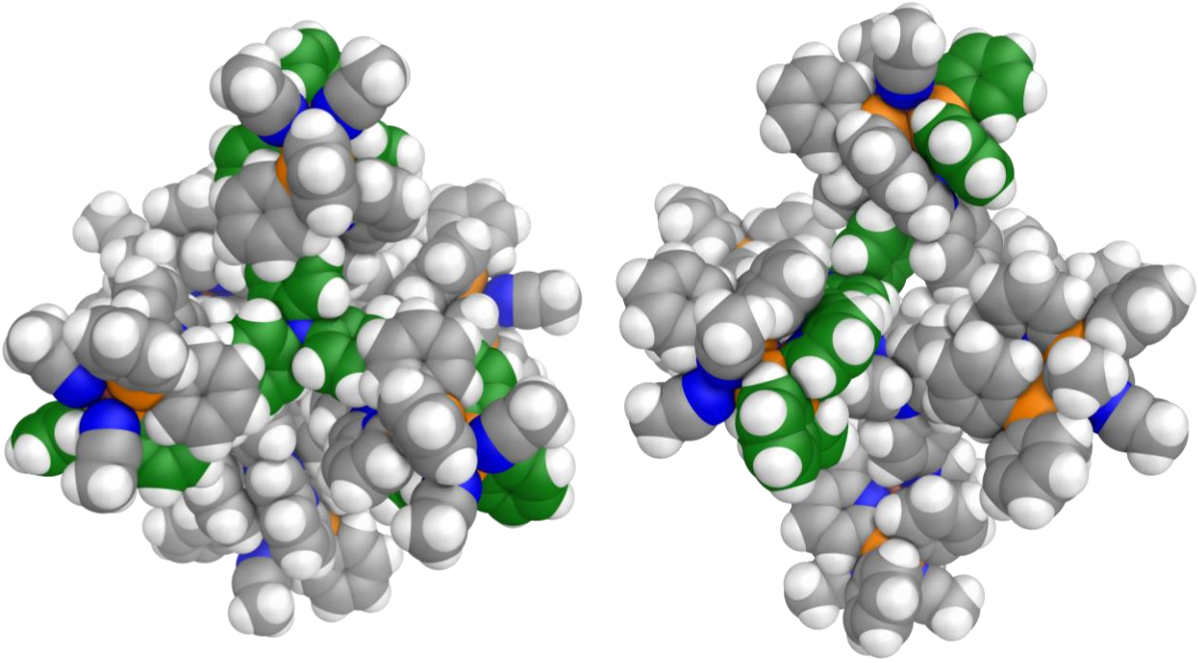

Figure S46: Space-filling representations of the cationic part of the crystal structure of **1** showing a view looking down one of the three-fold symmetric ligands (left) In both cases the carbon atoms of one of the ligands are highlighted in green.

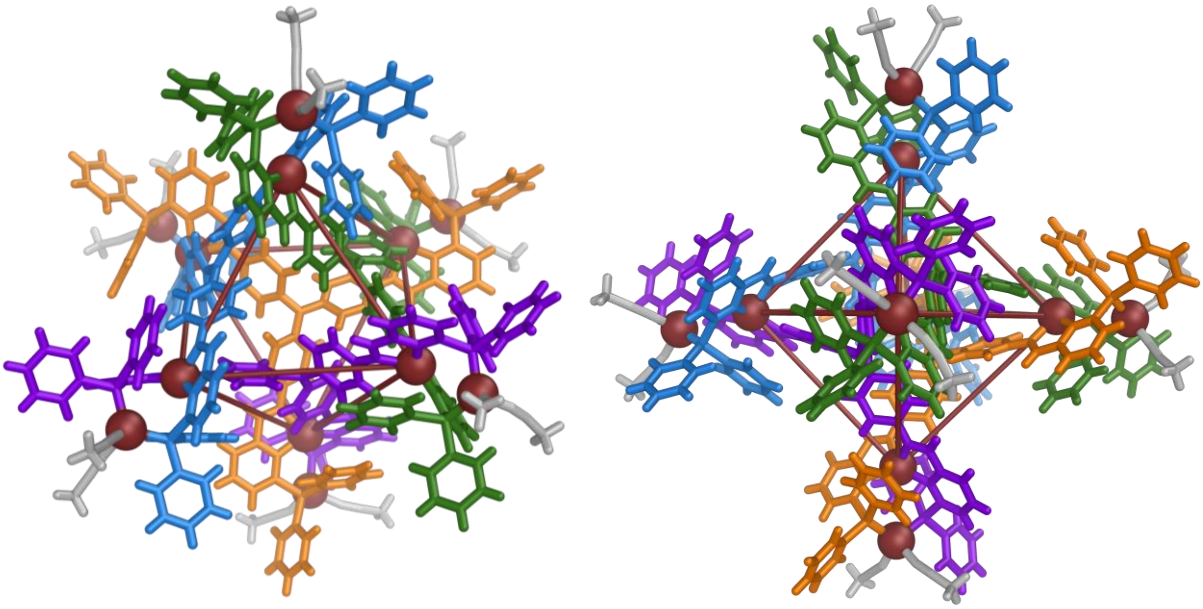

Figure S47: Two views of the cationic part of the crystal structure of **1**, looking down one of the types of C_3 axis (left) and a C_2 axis (right). In both cases each of the four ligands are rendered in a different colour and the coordinated acetonitrile ligands are coloured grey.

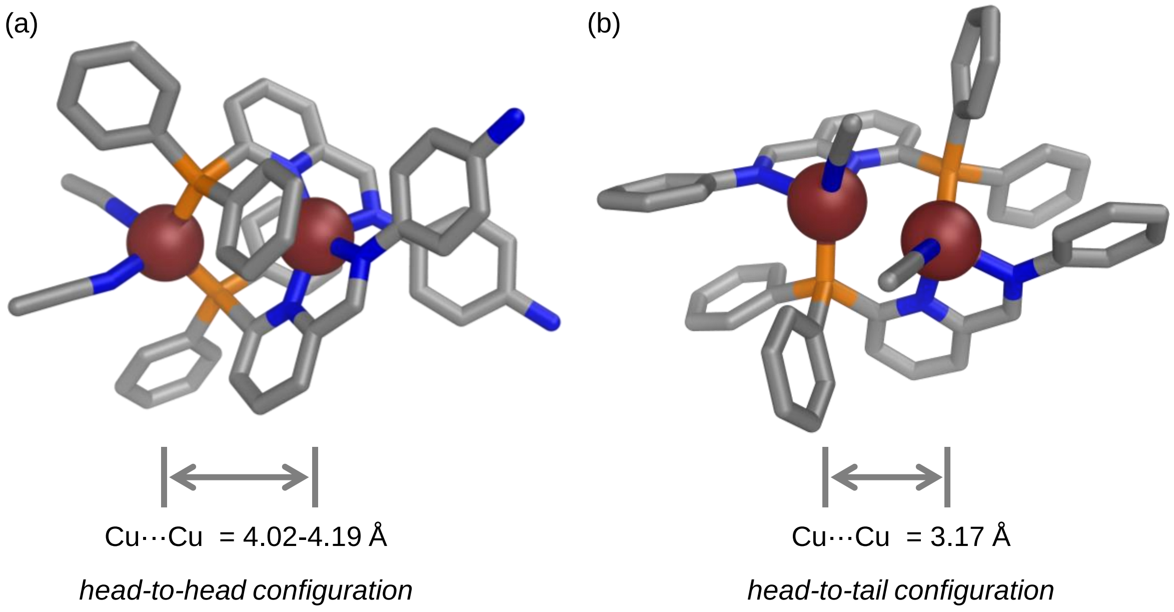

Figure S48: (a) Magnification of one of the vertices of **1** (left) which displays a head-to-head configuration. (b) Structure of the previously reported dicopper(I) complex[1] (right), formed from the self-assembly of **A**, aniline and a copper(I) salt, which displays a head-to-tail configuration.

Cage 1 (perchlorate salt) - $[Cu_{12}L_4(MeCN)_{12}]\cdot OTf\cdot 11ClO_4\cdot 0.5MeCN$ [+ solvent]

Formula $C_{314}H_{253.50}Cl_{11}Cu_{12}F_3N_{40.50}O_{47}P_{12}S$, *M* 6959.19, Cubic, space group P 2_1 3 (#198), *a* 43.2456(12), *b* 43.2456(12), *c* 43.2456(12) Å, *V* 80877(7) Å^3, D_c 1.143 g cm^{-3}, *Z* 8, crystal size 0.260 by 0.220 by 0.130 mm, colour brown, habit prism, temperature 180(2) Kelvin, λ(CuKα) 1.54178 Å, μ(CuKα) 2.344 mm^{-1}, T(SADABS)$_{min,max}$ 0.5366, 0.7488, $2\theta_{max}$ 79.92, *hkl* range -21 36, -22 34, -35 26, *N* 85849, N_{ind} 16141(R_{merge} 0.0480), N_{obs} 13730(I > 2σ(I)), N_{var} 2277, residuals* $R1(F)$ 0.0833, $wR2(F^2)$ 0.2392, GoF(all) 1.091, $\Delta\rho_{min,max}$ -0.372, 0.420 e$^-$ Å^{-3}.

*$R1 = \Sigma||F_o| - |F_c||/\Sigma|F_o|$ for $F_o > 2\sigma(F_o)$; $wR2 = (\Sigma w(F_o^2 - F_c^2)^2/\Sigma(wF_c^2)^2)^{1/2}$ all reflections

$w=1/[\sigma^2(F_o^2)+(0.1444P)^2+266.7279P]$ where $P=(F_o^2+2F_c^2)/3$

Specific refinement details:

The crystals with composition $[Cu_{12}L_4(MeCN)_{12}]\cdot OTf\cdot 11ClO_4\cdot 0.5MeCN$ [+ solvent] were grown by diffusion of diisopropyl ether into an acetonitrile solution of **1**·12OTf containing excess Bu_4NClO_4. The crystals employed immediately lost solvent after removal from the mother liquor and rapid handling prior to flash cooling in the cryostream was required to collect data. Despite these measures and the use

of a high intensity laboratory source few reflections at greater than 1.1 Å resolution were observed and the data were trimmed accordingly. Nevertheless, the quality of the data is far more than sufficient to establish the connectivity of the structure. This structure is isomorphous to $[Cu_{12}L_4(MeCN)_{12}]\cdot 11.7OTf\cdot 0.3ReO_4\cdot 2MeCN\cdot 1.5^{i}Pr_2O$ with respect to the main cation. The structure was refined as both a merohedral twin (twin law 0 1 0 1 0 0 0 0 -1) and a racemic twin with the BASF parameters refining to 0.29033/0.31292/0.20226.

Bond lengths and angles within pairs of chemically identical organic ligand arms were restrained to be similar to each other (SAME) and many aromatic rings were refined with rigid body constraints (AFIX 66). Additional restraints (DFIX, FLAT) were applied to some other sections of the organic ligands. Thermal parameter restraints (SIMU, RIGU) were applied to all atoms except for copper to facilitate anisotropic stable refinement. One of the coordinated acetonitrile molecules were modelled as disordered over two locations and others show evidence of thermal motion or minor unresolved disorder. These acetonitrile molecules were modelled with isotropic thermal parameters and bond length restraints (DFIX) were applied to all coordinated acetonitrile molecules.

Several anions were located on special positions and others were modelled with partial occupancy. In total only 5.25 anions per $Cu_{12}L_4$ assembly could be resolved from the electron density map. The remaining anions (included as perchlorate in the formula) and solvent within the lattice were significantly disordered and despite numerous attempts at modelling, including with rigid bodies no satisfactory model for the electron-density associated with them could be found. Consequently the SQUEEZE[12] function of PLATON[13] was employed to remove the contribution of the electron density associated with these highly disordered solvents and anions which gave a potential solvent accessible void of 29223 Å^3 per unit cell (a total of approximately 6602 electrons). Since the diffuse solvent molecules could not be assigned conclusively to acetonitrile or diisopropyl ether they were not included in the formula. Consequently, the molecular weight and density given above are likely to be underestimated.

CheckCIF gives two A and four B level alerts. These alerts all result from the limited data resolution and thermal motion and the disorder in the coordinated acetonitrile molecules as described above.

Cage 2 - $[Ag_{12}L_4(MeCN)_6]\cdot 12PF_6$ [+ solvent]

Formula $C_{300}H_{234}Ag_{12}F_{72}N_{34}P_{24}$, *M* 7720.92, Cubic, Fd–3 (#203), *a* 43.208(5), *b* 43.208(5), *c* 43.208(5) Å, *V* 80669(28) Å^3, D_c 1.271 g cm^{-3}, *Z* 8, crystal size 0.150 by 0.120 by 0.100 mm, colour purple, habit block, temperature 100(2) Kelvin, λ(Synchrotron) 0.6889 Å, μ(Synchrotron) 0.674 mm^{-1}, T(Analytical)$_{min,max}$ 0.9915, 1.0, $2\theta_{max}$ 36.46, *hkl* range -38 39, -39 39, -39 39, *N* 49148, N_{ind} 2657(R_{merge} 0.1386), N_{obs} 1688(I > 2σ(I)), N_{var} 379, residuals* $R1(F)$ 0.1008, $wR2(F^2)$ 0.3140, GoF(all) 1.088, $\Delta\rho_{min,max}$ -0.605, 1.104 e$^-$ Å^{-3}.

*$R1 = \Sigma||F_o| - |F_c||/\Sigma|F_o|$ for $F_o > 2\sigma(F_o)$; $wR2 = (\Sigma w(F_o^2 - F_c^2)^2/\Sigma(wF_c^2)^2)^{1/2}$ all reflections

$w=1/[\sigma^2(F_o^2)+(0.1500P)^2+2000.0000P]$ where $P=(F_o^2+2F_c^2)/3$

Specific refinement details:

Crystals with composition $[Ag_{12}L_4(MeCN)_6]\cdot 12PF_6$ were grown by slow diffusion of diethyl ether into a CD_3CN solution of **2**·12OTf. The crystals employed in this study rapidly lost solvent after removal from the mother liquor and rapid handling prior to flash cooling in the cryostream was required to collect data. Despite the use of high intensity synchrotron radiation, few reflections at greater than 1.1 Å resolution were observed. Nevertheless, the quality of the data is more than sufficient to establish the connectivity of the structure. The asymmetric unit was found to contain one twelfth of a $Ag_{12}L_4$ assembly (i.e. a single ligand arm) and associated counterions.

Further reflecting the solvent loss there is a significant amount of void volume in the lattice containing smeared electron density which was treated using SQUEEZE[12] function of PLATON.[13] The SQUEEZEd portion of the cell totals 2,672 electrons per unit cell, with a solvent accessible void volume of 16,600 Å^3 per unit cell. This equates to 334 electrons per cationic assembly, where *Z* = 8. As all the anions were identified in the electron density map, we attribute this residual density to unresolved solvent molecules. Since the diffuse solvent molecules could not be assigned conclusively to acetonitrile or diethyl ether they were not included in the formula. Consequently, the molecular weight and density given above are likely to be slightly underestimated.

CheckCIF produces 1 Level A and 3 Level B alerts. All these alerts are due to the high thermal motion of some atoms, leading to the less than ideal resolution of the data.

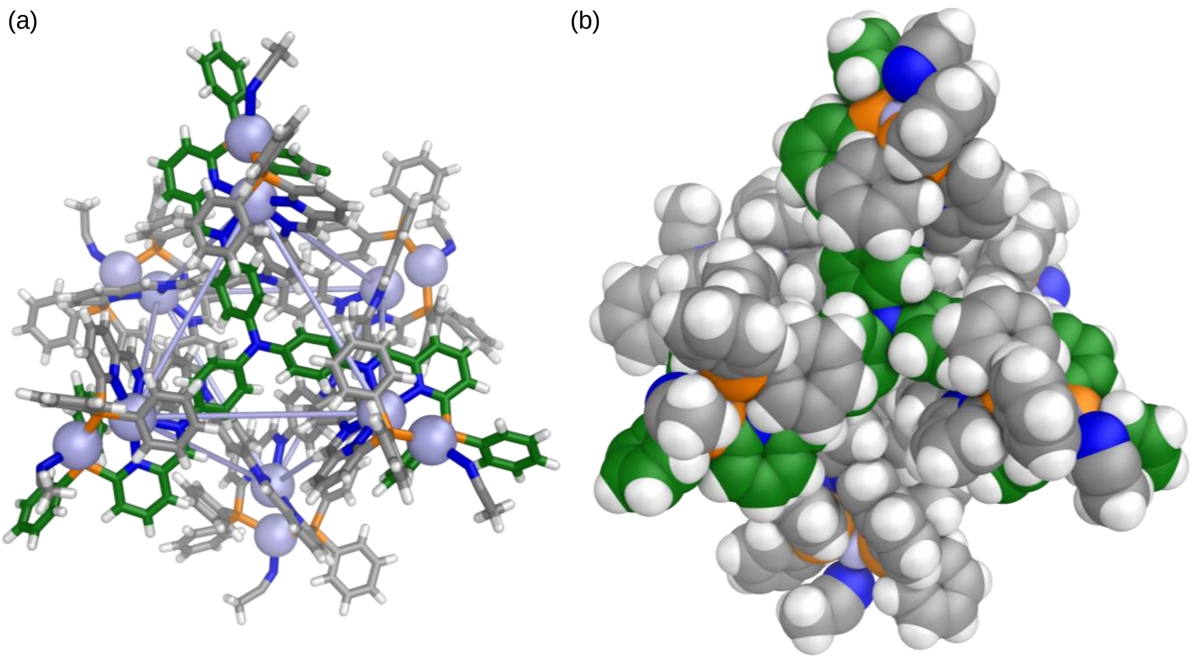

Figure S49: (a) Cationic part of the crystal structure of **2**. (b) Space-filling representation of the cationic part of the crystal structure of **2**. In both cases the carbon atoms of one of the ligands are highlighted in green.

Cage 3 - [$Cu_4Cd_4L_4(MeCN)_4$]·12OTf·4MeCN·0.5C_6H_6 [+ solvent]

Formula $C_{319}H_{243}Cd_4Cu_4F_{36}N_{36}O_{36}P_{12}S_{12}$, *M* 7300.60, Monoclinic, *C*2/*c* (#15), *a* 53.8714(4), *b* 42.1628(3), *c* 41.8428(3) Å, β 126.1960(10), *V* 76697.7(12) Å^3, D_c 1.264 g cm^{-3}, *Z* 8, crystal size 0.040 by 0.015 by 0.010 mm, colour red, habit block, temperature 100(2) Kelvin, λ(Synchrotron) 0.6889 Å, μ(Synchrotron) 0.573 mm^{-1}, T(Analytical)$_{min,max}$ 1.2487, 22.3477, $2\theta_{max}$ 36.50, *hkl* range -48 48, -38 38, -37 37, *N* 99482, N_{ind} 30117(R_{merge} 0.1444), N_{obs} 19038(I > 2σ(I)), N_{var} 4136, residuals* *R*1(*F*) 0.1090, *wR*2(F^2) 0.3429, GoF(all) 1.185, $\Delta\rho_{min,max}$ -1.174, 1.654 e$^-$ Å^{-3}.

*$R1 = \Sigma||F_o| - |F_c||/\Sigma|F_o|$ for $F_o > 2\sigma(F_o)$; $wR2 = (\Sigma w(F_o^2 - F_c^2)^2/\Sigma(wF_c^2)^2)^{1/2}$ all reflections

$w=1/[\sigma^2(F_o^2)+(0.2000P)^2]$ where $P=(F_o^2+2F_c^2)/3$

Specific refinement details:

Crystals with composition [$Cu_4Cd_4L_4(MeCN)_4$]·12OTf·4MeCN·0.5C_6H_6 [+ solvent] were grown by slow diffusion of benzene into a CD_3CN solution of **3**·12OTf. The crystals employed in this study

rapidly lost solvent after removal from the mother liquor and rapid handling prior to flash cooling in the cryostream was required to collect data. Despite the use of high intensity synchrotron radiation, few reflections at greater than 1.1 Å resolution were observed. Nevertheless, the quality of the data is more than sufficient to establish the connectivity of the structure.

Due to the less than ideal resolution, bond lengths and angles within pairs of organic ligands were restrained to be similar to each other (SAME) and thermal parameter restraints (SIMU, DELU) were applied to all non-metal atoms to facilitate anisotropic refinement. Ligand-based atoms that still displayed thermal parameters greater than 0.4 were further refined to approximate isotropic behaviour (ISOR) and the thermal parameters of several anions were restrained with RIGU. Anti-bumping restraints (BUMP) were also applied. Four triflate anions were modelled as disordered over two positions. The benzene molecule was modelled in half occupancy.

The remaining triflate anions present in the asymmetric unit could not be successfully resolved despite numerous attempts at modelling, including the use of rigid bodies. Consequently, the SQUEEZE[12] function of PLATON[13] was employed to remove the contribution of the electron density associated with the remaining anions and further highly disordered solvent molecules. The SQUEEZEd portion of the cell totals 5,858 electrons per unit cell, with a solvent accessible void volume of 20,063 Å^3 per unit cell. This equates to 732 electrons per structure, where $Z = 8$. We attribute this excess density to the 3 counterions that could not be identified in the electron density map and further highly disordered solvent molecules. Since the diffuse solvent molecules could not be assigned conclusively to acetonitrile or benzene they were not included in the formula. Consequently, the molecular weight and density given above are likely to be slightly underestimated.

CheckCIF produces 1 Level A and 3 Level B alerts. All these alerts are due to the highly disordered counterions and high thermal motion of some atoms, leading to the less than ideal resolution of the data. One alert from the large average U_{eq} of a triflate anions results from a high level of thermal motion or minor unresolved disorder that was not modelled due to the limited resolution of the data.

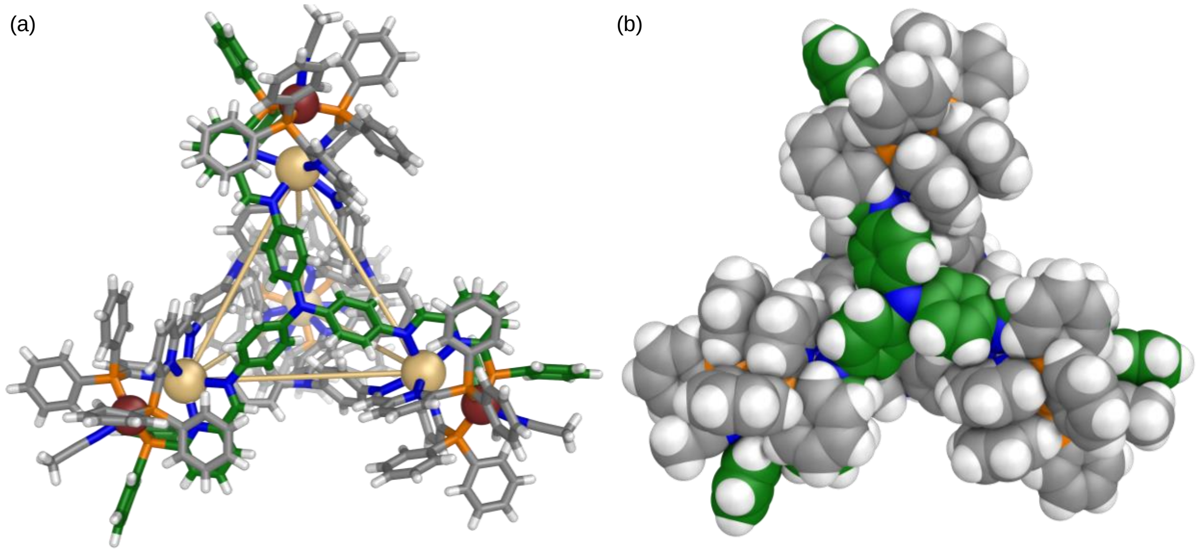

Figure S50: (a) Cationic part of the crystal structure of **3**. (b) Space-filling representations of the cationic part of the crystal structure of **3**. In both cases the carbon atoms of one of the ligands are highlighted in green.

Cage 4 - [C_6H_{12}⊂$Cu_4Cd_4L_4(MeCN)_4$]·12OTf·3.75MeCN [+ solvent]

Formula $C_{345.50}H_{287.25}Cd_4Cu_4F_{36}N_{55.75}O_{36}P_{12}S_{12}$, *M* 7940.16, Monoclinic, space group P 21/n (#14), *a* 20.229(4), *b* 47.904(10), *c* 40.646(8) Å, β 90.58(3), *V* 39386(14) Å^3, D_c 1.339 g cm^{-3}, *Z* 4, crystal size 0.010 by 0.010 by 0.010 mm, colour orange, habit block, temperature 100(2) Kelvin, λ(Synchrotron) 0.6889 Å, μ(Synchrotron) 0.565 mm^{-1}, T(Analytical)$_{min,max}$ 0.942873565376, 1.0, $2\theta_{max}$ 40.30, *hkl* range -20 20, -47 47, -40 40, *N* 194036, N_{ind} 40805(R_{merge} 0.0913), N_{obs} 28502(I > 2σ(I)), N_{var} 4529, residuals* $R1(F)$ 0.1340, $wR2(F^2)$ 0.3948, GoF(all) 1.062, $\Delta\rho_{min,max}$ -1.525, 1.999 e$^-$ Å^{-3}.

*$R1 = \Sigma||F_o| - |F_c||/\Sigma|F_o|$ for $F_o > 2\sigma(F_o)$; $wR2 = (\Sigma w(F_o^2 - F_c^2)^2/\Sigma(wF_c^2)^2)^{1/2}$ all reflections

$w=1/[\sigma^2(F_o^2)+(0.1946P)^2+491.1736P]$ where $P=(F_o^2+2F_c^2)/3$

Specific refinement details:

The crystals with composition [C_6H_{12}⊂$Cu_4Cd_4L_4(MeCN)_4$]·12OTf·3.75MeCN [+ solvent] were grown by diffusion of diisopropyl ether into an acetonitrile solution of **4**·12OTf containing cyclohexane. The crystals employed immediately lost solvent after removal from the mother liquor and rapid handling prior to flash cooling in the cryostream was required to collect data. Despite these measures and the use

of synchrotron radiation few reflections at greater than 1.0 Å resolution were observed and the data were trimmed accordingly. The crystals were small and were subject to beam damage during data collection using synchrotron radiation; consequently the quality of the integration is less than ideal with relatively high residuals. Nevertheless, the quality of the data is far more than sufficient to establish the connectivity of the structure.

Bond lengths and angles within pairs of chemically identical organic ligands were restrained to be similar to each other (SAME) and some aromatic rings were refined with rigid body constraints (AFIX 66). Thermal parameter restraints (SIMU, RIGU) were applied to all atoms except for copper and cadmium to facilitate anisotropic stable refinement. Even with these restraints some thermal parameters remain larger than ideal as a consequence of the high level of thermal motion or minor unresolved disorder present throughout the structure. The encapsulated cyclohexane molecule was modelled as disordered over two locations with opposite orientations of the chair conformation. Bond lengths and angles within the disordered cyclohexane were restrained (DFIX, DANG) in order to obtain a reasonable geometry.

The anions within the structure also show evidence of substantial disorder. Seven triflate anions were modelled as disordered over two or three locations and several others were modelled with partial occupancy. The occupancies of the all the located anions were allowed to refine freely and then fixed at the obtained values. Some additional minor occupancy positions of the anions could not be located in the electron density map and were not included in the model resulting in a discrepancy of 4.55 anions per $Cu_4Cd_4L_4$ assembly; these anions are assigned as triflate in the formula given above. Some lower occupancy disordered atoms were modelled with isotropic thermal parameters and substantial bond length and thermal parameter restraints were required to facilitate realistic modelling of the disordered anions. Some acetonitrile solvent molecules were also modelled as disordered and/or with partial occupancy. The hydrogen atoms on some disordered acetonitrile molecules could not be resolved and were thus not included in the model.

Further reflecting the solvent loss there is a significant amount of void volume in the lattice containing smeared electron density from disordered solvent (and potentially the unresolved anions). Consequently the SQUEEZE[12] function of PLATON[13] was employed to remove the contribution of the electron density associated with this highly disordered solvent which gave a potential solvent accessible void of 6276 Å^3 per unit cell (a total of approximately 1895 electrons). Since the diffuse solvent molecules could not be assigned conclusively to acetonitrile or diisopropyl ether they were not included in the formula. Consequently, the molecular weight and density given above are likely to be underestimated.

CheckCIF gives one A and four B level alerts. These alerts all result from the limited data resolution, beam damage, thermal motion or minor unresolved disorder present throughout the structure and the disordered acetonitrile molecules for which hydrogen atoms were not modelled as described above.

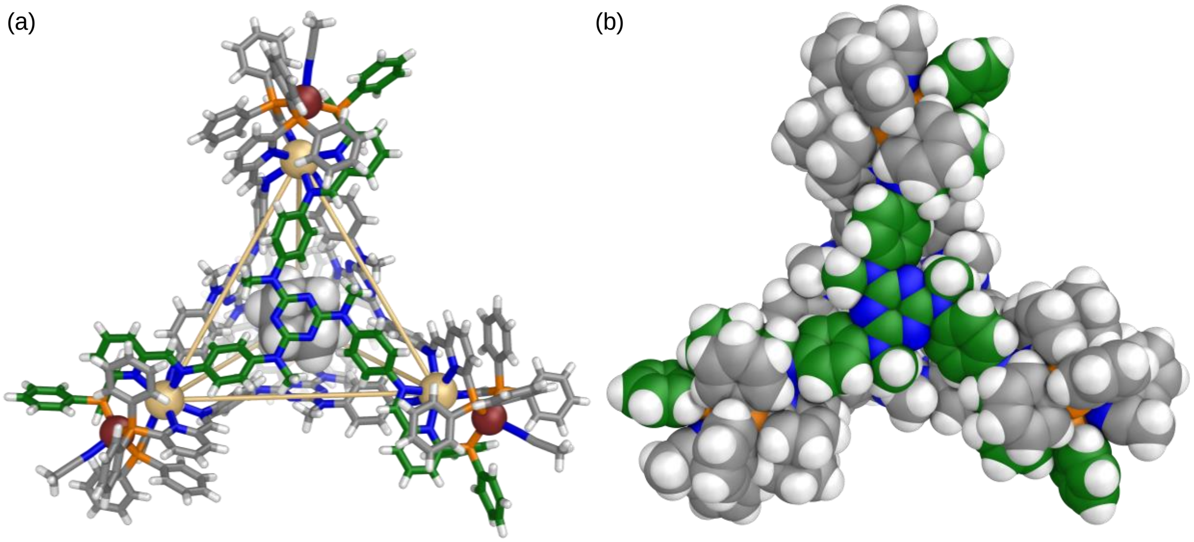

Figure S51: (a) Cationic part of the crystal structure of **4** showing the cyclohexane bound within the cage cavity in space-filling representation. (b) Space-filling representation of the cationic part of the crystal structure of **4**. In both cases the carbon atoms of one of the ligands are highlighted in green.

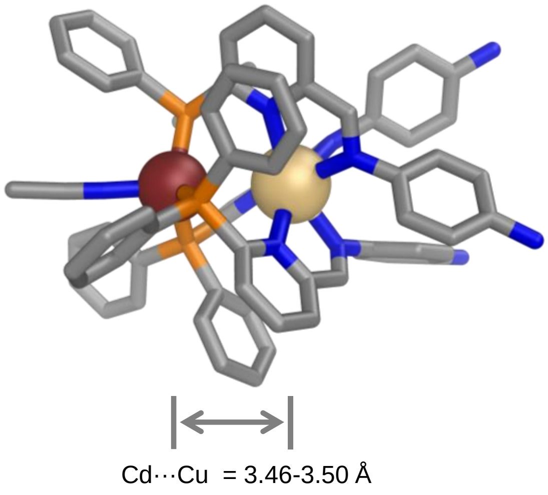

Figure S52: Magnification of one of the vertices of **4**.

3. Molecular modelling

Noting that the head-to-tail configuration was observed in previous structures incorporating **A**[1] and most other related dicopper complexes,[14] we sought to model a hypothetical *pseudo*-octahedral structure **1′** with a head-to-tail arrangement of **A** residues at its vertices.

A molecular mechanics model of the hypothetical structure of **1′** was optimised using the universal force field (UFF) in ArgusLabs Version 4.0.1[15] with search type BFGS. All other parameters were set at their default values.

The resulting model (see Figure S53) suggests that the coordinated acetonitrile ligands required to complete the tetrahedral coordination sphere of the Cu^{I} centers would have to be incorporated into the cavity of the structure requiring substantial expansion of the tritopic ligand panels which must bend outwards. We infer this arrangement would engender steric strain within the structure and is therefore unfavorable compared to the more compact structure of **1** which allows the acetonitrile ligands to coordinate externally to the cavity and minimises the overall cavity size.

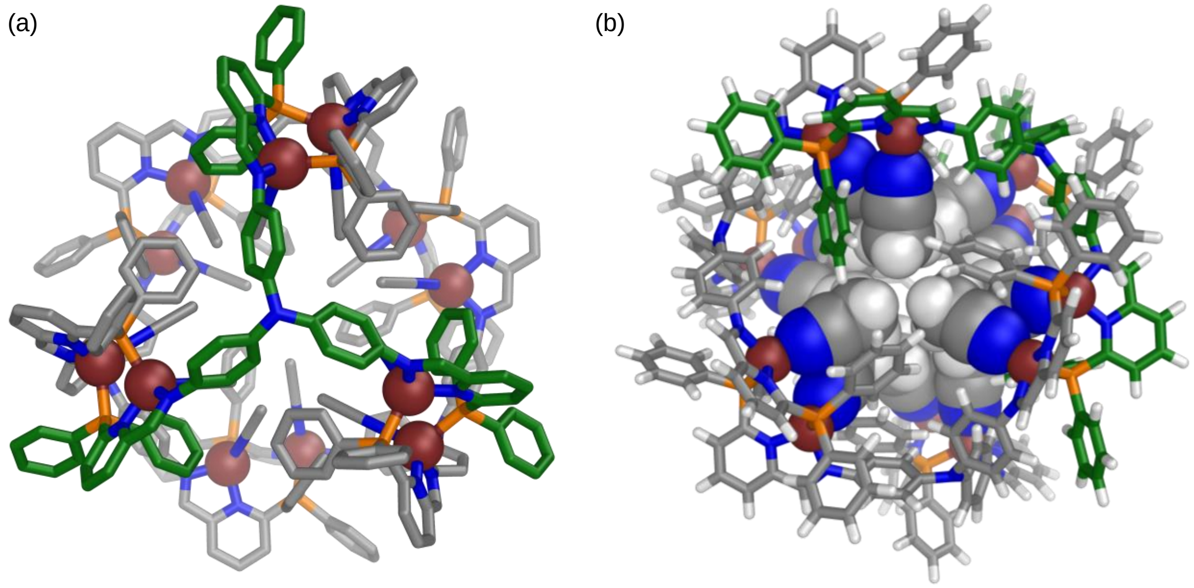

Figure S53: Two views of the UFF optimised molecular model of **1′**. In both cases the carbon atoms of one of the ligands are highlighted in green. (a) View looking down one of the ligand occupied faces with hydrogen atoms omitted for clarity. (b) View looking down one of the empty faces with the coordinated acetonitrile molecules shown in space-filling representation, highlighting the steric crowding within the cavity of **1′**.

4. Volume calculations

In order to determine the available void spaces within the structures of cages **1-4**, Molovol[16] calculations based on the crystal structures obtained in this study were performed with guests, anions, disorder and solvent molecules removed. A virtual probe with a radius of 1.2 Å was employed in all cases with a grid resolution of 0.1 Å and optimization depth of 4. All other parameters were set at their default values. The results are shown in Figures S54.

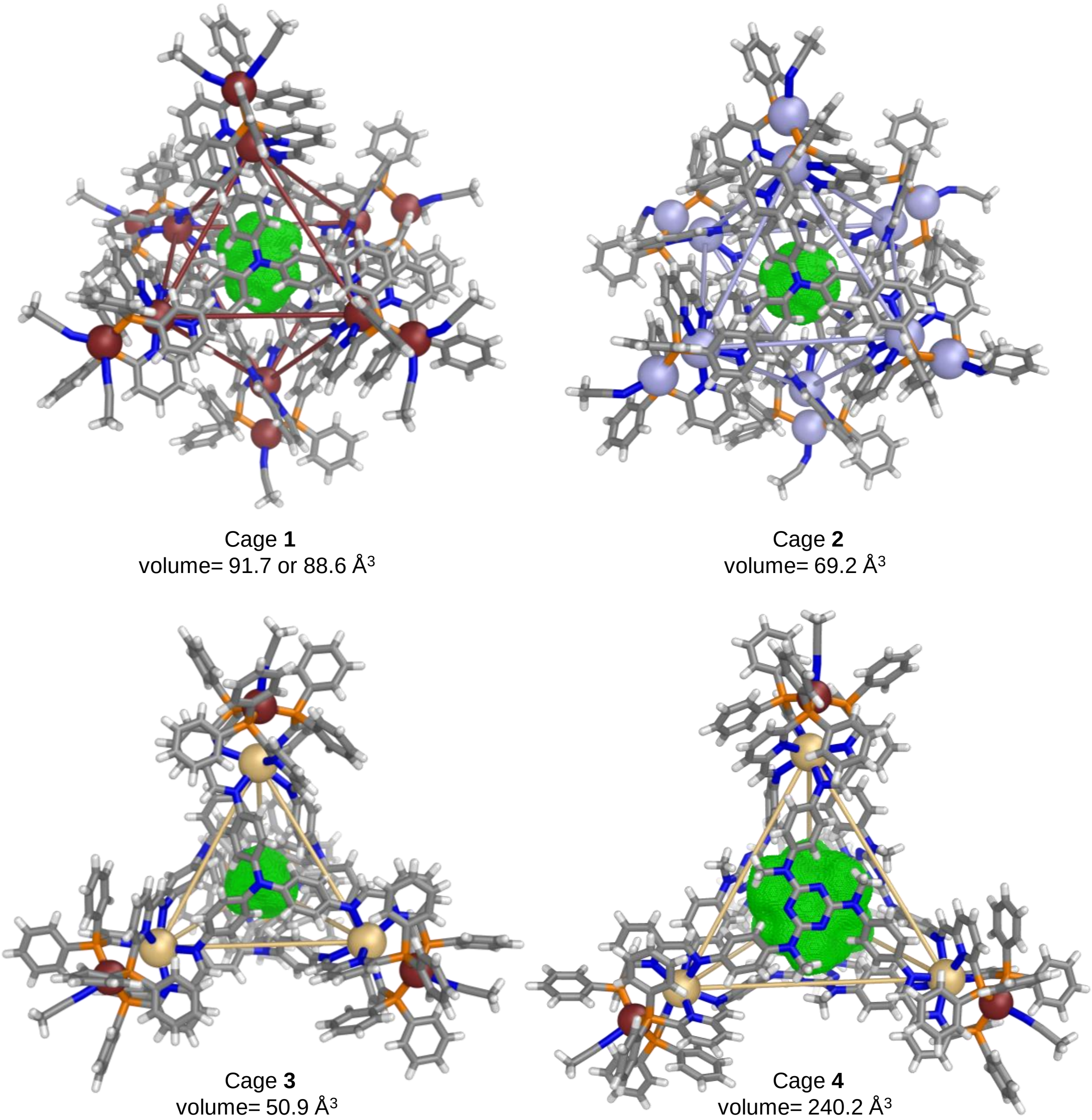

Figure S54: Molovol calculated void spaces (shown in green mesh) within the crystal structures of cages **1-4.** Only one of the two crystallographically unique complexes is shown for **1** but the volumes of both complexes are listed.

5. References

1. Dry, E. F. V.; Clegg, J. K.; Breiner, B.; Whitaker, D. E.; Stefak, R.; Nitschke, J. R., Reversible anion-templated self-assembly of [2+2] and [3+3] metallomacrocycles containing a new dicopper(I) motif. *Chem. Commun.* **2011,** *47*, 6021.
2. (a) Ryan, H. P.; Haynes, C. J. E.; Smith, A.; Grommet, A. B.; Nitschke, J. R., Guest Encapsulation within Surface-Adsorbed Self-Assembled Cages. *Adv. Mater.* **2021,** *33*, 2004192; (b) Bolliger, J. L.; Ronson, T. K.; Ogawa, M.; Nitschke, J. R., Solvent Effects upon Guest Binding and Dynamics of a $Fe^{II}_4L_4$ Cage. *J. Am. Chem. Soc.* **2014,** *136*, 14545.
3. Allan, D.; Nowell, H.; Barnett, S.; Warren, M.; Wilcox, A.; Christensen, J.; Saunders, L.; Peach, A.; Hooper, M.; Zaja, L.; Patel, S.; Cahill, L.; Marshall, R.; Trimnell, S.; Foster, A.; Bates, T.; Lay, S.; Williams, M.; Hathaway, P.; Winter, G.; Gerstel, M.; Wooley, R., A Novel Dual Air-Bearing Fixed-χ Diffractometer for Small-Molecule Single-Crystal X-ray Diffraction on Beamline I19 at Diamond Light Source. *Crystals* **2017,** *7*, 336.
4. (a) Collaborative Computational Project, N., The CCP4 suite: programs for protein crystallography. *Acta Cryst.* **1994,** *D50*, 760; (b) Evans, P., Scaling and assessment of data quality. *Acta Cryst.* **2006,** *D62*, 72; (c) Winter, G., xia2: an expert system for macromolecular crystallography data reduction. *J. Appl. Crystallogr.* **2010,** *43*, 186.
5. Evans, P. R.; Murshudov, G. N., How good are my data and what is the resolution? *Acta Cryst.* **2013,** *D69*, 1204.
6. Winn, M. D.; Ballard, C. C.; Cowtan, K. D.; Dodson, E. J.; Emsley, P.; Evans, P. R.; Keegan, R. M.; Krissinel, E. B.; Leslie, A. G. W.; McCoy, A.; McNicholas, S. J.; Murshudov, G. N.; Pannu, N. S.; Potterton, E. A.; Powell, H. R.; Read, R. J.; Vagin, A.; Wilson, K. S., Overview of the CCP4 suite and current developments. *Acta Cryst.* **2011,** *D67*, 235.
7. Bruker-Nonius, *APEX, SAINT and XPREP*. Bruker AXS Inc.: Madison, Wisconsin, USA, 2013.
8. Farrugia, L., WinGX and ORTEP for Windows: an update. *J. Appl. Crystallogr.* **2012,** *45*, 849.
9. Sheldrick, G., SHELXT - Integrated space-group and crystal-structure determination. *Acta. Cryst.* **2015,** *A71*, 3.
10. Palatinus, L.; Chapuis, G., SUPERFLIP - a computer program for the solution of crystal structures by charge flipping in arbitrary dimensions. *J. Appl. Crystallogr.* **2007,** *40*, 786.
11. Sheldrick, G. M., Crystal structure refinement with SHELXL. *Acta. Cryst.* **2015,** *C71*, 3.
12. van der Sluis, P.; Spek, A. L., BYPASS: an effective method for the refinement of crystal structures containing disordered solvent regions. *Acta Cryst.* **1990,** *A46*, 194.
13. Spek, A. L., *PLATON: A Multipurpose Crystallographic Tool*. Utrecht University: Utrecht, The Netherlands, 2008.
14. (a) Kuang, S.-M.; Zhang, Z.-Z.; Wang, Q.-G.; Mak, T. C. W., Reaction of $[Cu_2(\mu\text{-}Ph_2Ppypz)_2(MeCN)_2](ClO_4)_2$ (Ph_2Ppypz=(2-diphenylphosphino-6-pyrazol-1-yl)pyridine) with $Fe(CO)_4^{2-}$ and X^- (X=Cl, I, $MeCO_2$ and pyrazolate). *J. Organomet. Chem.* **1998,** *558*, 131; (b) Kuang, S.-M.; Zhang, Z.-Z.; Wang, Q.-G.; C. W. Mak, T., Synthesis and reactivity of binuclear copper(I) complexes of 2-(diphenylphosphino)-6-(pyrazol-1-yl)pyridine (L^1). Crystal structures of $[Cu_2(\mu\text{-}L^1)_2(MeCN)_2][ClO_4]_2$, $[Cu_2(\mu\text{-}L^1)_2(\mu\text{-}\eta^1\text{-}C{\equiv}CPh)][ClO_4]\cdot CHCl_3\cdot H_2O$ and $[Cu_2(\mu\text{-}L^1)_2(\mu\text{-}Cl)][ClO_4]\cdot H_2O$. *J. Chem. Soc., Dalton Trans.* **1998**, 1115; (c) Field, J. S.; Haines, R. J.; Parry, C. J.; Sookraj, S. H., Dicopper(I) complexes of the novel phosphorusbipyridyl ligand 6-diphenylphosphino-2,2′-bipyridyl. *Polyhedron* **1993,** *12*, 2425; (d) Lilio, A. M.; Grice, K. A.; Kubiak, C. P., A Series of Dinuclear Copper Complexes Bridged by Phosphanylbipyridine Ligands: Synthesis, Structural Characterization and Electrochemistry. *Eur. J. Inorg. Chem.* **2013,** *2013*, 4016.
15. Thompson, M., *ArgusLab Version 4.0.1*. Planaria Software LLC: Seattle, WA 1997.
16. Maglic, J. B.; Lavendomme, R., MoloVol: an easy-to-use program to calculate various volumes and surface areas of chemical structures and identify cavities. *ChemRxiv* **2021**. DOI: 10.33774/chemrxiv-2021-dss1j.
